# Supplementary material for: Delayed antibiotic prescribing for respiratory tract infections: protocol of an individual patient data meta-analysis
Source: BMJ Open. 2019 Jan 21;9(1):e026925. doi: 10.1136/bmjopen-2018-026925 (PMC6347865; doi:10.1136/bmjopen-2018-026925)
Supplement: Supplementary data [file bmjopen-2018-026925supp001.pdf]

**Supplementary Online Appendix 1. CENTRAL, MEDLINE, Embase, CINAHL, and Web of Science search strategies for randomised controlled trials (November 2017)**

**Cochrane Central Register of Controlled Trials (CENTRAL)**
#1 MeSH descriptor: [Respiratory Tract Infections] explode all trees
#2 ((upper next respiratory next tract next infection*) or URTI):ti,ab,kw
#3 MeSH descriptor: [Otitis Media] explode all trees
#4 (otitis next media):ti,ab,kw
#5 MeSH descriptor: [Pharyngitis] explode all trees
#6 pharyngitis:ti,ab,kw
#7 MeSH descriptor: [Tonsillitis] explode all trees
#8 tonsillitis:ti,ab,kw
#9 MeSH descriptor: [Common Cold] explode all trees
#10 (common next cold*):ti,ab,kw
#11 MeSH descriptor: [Bronchitis] explode all trees
#12 bronchitis:ti,ab,kw
#13 MeSH descriptor: [Sinusitis] explode all trees
#14 sinusitis:ti,ab,kw
#15 (sore next throat*):ti,ab,kw
#16 #1 or #2 or #3 or #4 or #5 or #6 or #7 or #8 or #9 or #10 or #11 or #12 or #13 or #14 or #15
#17 MeSH descriptor: [Anti-Bacterial Agents] explode all trees
#18 antibiotic*:ti,ab,kw
#19 #17 or #18
#20 (delay* near/15 prescri*):ti,ab,kw
#21 #16 and #19 and #20

**Ovid MEDLINE**
1 exp Respiratory Tract Infections/
2 (upper respiratory tract infection$ or urti).mp.
3 exp Otitis Media/
4 otitis media.mp.
5 exp Pharyngitis/
6 pharyngitis.mp.
7 exp Tonsillitis/
8 tonsillitis.mp.
9 exp Common Cold/
10 common cold.mp.
11 exp Bronchitis/
12 bronchitis.mp.
13 exp Sinusitis/
14 sinusitis.mp.
15 sore throat$.mp.
16 or/1-15
17 exp Anti-Bacterial Agents/
18 antibiotic$.mp.
19 or/17-18
20 (delay$ adj15 prescri$).mp.
21 16 and 19 and 20
22 (2013* or 2014* or 2015* or 2016* or 2017*).ed.
23 21 and 22

**Ovid Embase**
1 exp Respiratory Tract Infection/
2 exp Upper Respiratory Tract Infection/
3 (upper respiratory tract infection$ or urti).mp.
4 exp Otitis Media/
5 otitis media.mp.

6 exp Pharyngitis/
7 pharyngitis.mp.
8 exp Tonsillitis/
9 tonsillitis.mp.
10 exp Common Cold/
11 common cold.mp.
12 exp Bronchitis/
13 bronchitis.mp.
14 exp Sinusitis/
15 sinusitis.mp.
16 sore throat$.mp.
17 or/1-16
18 exp antibiotic agent/
19 antibiotic$.mp.
20 or/18-19
21 (delay$ adj15 prescri$).mp.
22 17 and 20 and 21
23 (2013* or 2014* or 2015* or 2016* or 2017*).em.
24 22 and 23

**EBSCO CINAHL Plus**
S15 S10 and S13 and S14
S14 TI delay* N15 prescri* or AB delay* N15 prescri*
S13 S11 or S12
S12 TI antibiotic* or AB antibiotic*
S11 (MH “Antibiotics+”)
S10 S1 or S2 or S3 or S4 or S5 or S6 or S7 or S8 or S9
S9 TI (otitis media or pharyngitis or tonsillitis or common cold* or bronchitis or sinusitis or sore throat*) or AB (otitis media or pharyngitis or tonsillitis or common cold* or bronchitis or sinusitis or sore throat*)
S8 (MH “Sinusitis+”)
S7 (MH “Bronchitis+”)
S6 (MH “Common Cold”)
S5 (MH “Tonsillitis+”)
S4 (MH “Pharyngitis”)
S3 (MH “Otitis Media+”)
S2 TI ( upper respiratory tract infection* or urti ) or AB ( upper respiratory tract infection* or urti )
S1 (MH “Respiratory Tract Infections+”)

**Web of Science**
#15#14 AND #11 AND #10
#14#13 OR #12
#13TS=prescri*
#12TS=delay*
#11TS=antibiotic*
#10#9 OR #8 OR #7 OR #6 OR #5 OR #4 OR #3 OR #2 OR #1
#9TS=“sore throat*”
#8TS=sinusitis
#7TS=bronchitis
#6TS=“common cold”
#5TS=tonsillitis
#4TS=pharyngitis
#3TS=“otitis media”
#2TS=urti
#1TS=“Respiratory Tract Infection*”

**Supplementary Online Appendix 2. CENTRAL, MEDLINE, Embase, CINAHL, and Web of Science search strategies for observational studies (November 2017)**

**Cochrane Central Register of Controlled Trials (CENTRAL)**
#1 MeSH descriptor: [Respiratory Tract Infections] explode all trees
#2 ((upper next respiratory next tract next infection*) or URTI):ti,ab,kw
#3 MeSH descriptor: [Otitis Media] explode all trees
#4 (otitis next media):ti,ab,kw
#5 MeSH descriptor: [Pharyngitis] explode all trees
#6 pharyngitis:ti,ab,kw
#7 MeSH descriptor: [Tonsillitis] explode all trees
#8 tonsillitis:ti,ab,kw
#9 MeSH descriptor: [Common Cold] explode all trees
#10 (common next cold*):ti,ab,kw
#11 MeSH descriptor: [Bronchitis] explode all trees
#12 bronchitis:ti,ab,kw
#13 MeSH descriptor: [Sinusitis] explode all trees
#14 sinusitis:ti,ab,kw
#15 (sore next throat*):ti,ab,kw
#16 #1 or #2 or #3 or #4 or #5 or #6 or #7 or #8 or #9 or #10 or #11 or #12 or #13 or #14 or #15
#17 MeSH descriptor: [Anti-Bacterial Agents] explode all trees
#18 antibiotic*:ti,ab,kw
#19 #17 or #18
#20 (delay* near/15 prescri*):ti,ab,kw
#21 #16 and #19 and #20
#22 Epidemiologic Studies/
#23 exp Case-Control Studies/
#24 exp Cohort Studies/
#25 Cross-Sectional Studies/
#26 (epidemiologic adj (study or studies)).ab,ti.
#27 case control.ab,ti.
#28 (cohort adj (study or studies)).ab,ti.
#29 cross sectional.ab,ti.
#30 cohort analy$.ab,ti.
#31 (follow up adj (study or studies)).ab,ti.
#32 longitudinal.ab,ti.
#33 retrospective$.ab,ti.
#34 prospective$.ab,ti.
#35 (observ$ adj3 (study or studies)).ab,ti.
#36 adverse effect.ab,ti.
#37 #22 or #23 or #24 or #25 or #26 or #27 or #28 or #29 or #30 or #31 or #32 or #33 or #34 or #35 or #36
#38 #21 and #37

**Ovid MEDLINE**
1 exp Respiratory Tract Infections/
2 (upper respiratory tract infection$ or urti).mp.
3 exp Otitis Media/
4 otitis media.mp.
5 exp Pharyngitis/
6 pharyngitis.mp.
7 exp Tonsillitis/
8 tonsillitis.mp.
9 exp Common Cold/
10 common cold.mp.
11 exp Bronchitis/
12 bronchitis.mp.

13 exp Sinusitis/
14 sinusitis.mp.
15 sore throat$.mp.
16 or/1-15
17 exp Anti-Bacterial Agents/
18 antibiotic$.mp.
19 or/17-18
20 (delay$ adj15 prescri$).mp.
21 16 and 19 and 20
22 (2013* or 2014* or 2015* or 2016* or 2017*).ed.
23 21 and 22
24 Epidemiologic studies/
25 Exp case control studies/
26 Exp cohort studies/
27 Cross-sectional studies/
28 Case control.tw.
29 (cohort adj (study or studies or analys*)).tw.
30 Cohort analy$.tw.
31 ((Follow up or observational or uncontrolled or non randomi#ed or nonrandomi#ed or epidemiologic*) adj (study or studies)).tw
32 (longitudinal or retrospective or prospective or cross sectional) adj (study or studies or review or analys* or cohort*)).tw.
33 Longitudinal.tw.
34 Retrospective*.tw.
35 Prospective*.tw
36 Cross sectional.tw.
37 Follow-Up Studies/
38 correlational study.mp
39 Or/24-38
40 23 and 39

**Ovid Embase**
1 exp Respiratory Tract Infection/
2 exp Upper Respiratory Tract Infection/
3 (upper respiratory tract infection$ or urti).mp.
4 exp Otitis Media/
5 otitis media.mp.
6 exp Pharyngitis/
7 pharyngitis.mp.
8 exp Tonsillitis/
9 tonsillitis.mp.
10 exp Common Cold/
11 common cold.mp.
12 exp Bronchitis/
13 bronchitis.mp.
14 exp Sinusitis/
15 sinusitis.mp.
16 sore throat$.mp.
17 or/1-16
18 exp antibiotic agent/
19 antibiotic$.mp.
20 or/18-19
21 (delay$ adj15 prescri$).mp.
22 17 and 20 and 21
23 (2013* or 2014* or 2015* or 2016* or 2017*).em.
24 22 and 23

25 Clinical study/
26 exp Case control study/
27 Family study/
28 Longitudinal study/
29 Retrospective study/
30 Prospective study/
31 Randomized controlled trials/
32 30 not 31
33 Cohort analysis/
34 (Cohort adj (study or studies)).mp.
35 (Case control adj (study or studies)).tw.
36 ((Follow up or observational or uncontrolled or non randomi#ed or nonrandomi#ed or epidemiologic*) adj (study or studies)).tw
37 (longitudinal or retrospective or prospective or cross sectional) adj (study or studies or review or analys* or cohort*)).tw.
38 Follow-up/
39 38 and 33
40 correlational study.mp
41 Or/25-30,32-37,39-40
42 24 and 41

**EBSCO CINAHL Plus**
S24 S15 and S23
S23 or/S16-S22
S22 (observational adj (study or studies)).tw.
S21 (cohort adj (study or studies)).tw.
S20 Cross sectional studies/
S19 Nonconcurrent prospective studies/
S18 Correlational studies/
S17 Exp case control studies/
S16 Prospective studies/
S15 S10 and S13 and S14
S14 TI delay* N15 prescri* or AB delay* N15 prescri*
S13 S11 or S12
S12 TI antibiotic* or AB antibiotic*
S11 (MH "Antibiotics+")
S10 S1 or S2 or S3 or S4 or S5 or S6 or S7 or S8 or S9
S9 TI (otitis media or pharyngitis or tonsillitis or common cold* or bronchitis or sinusitis or sore throat*) or AB (otitis media or pharyngitis or tonsillitis or common cold* or bronchitis or sinusitis or sore throat*)
S8 (MH "Sinusitis+")
S7 (MH "Bronchitis+")
S6 (MH "Common Cold")
S5 (MH "Tonsillitis+")
S4 (MH "Pharyngitis")
S3 (MH "Otitis Media+")
S2 TI ( upper respiratory tract infection* or urti ) or AB ( upper respiratory tract infection* or urti )
S1 (MH "Respiratory Tract Infections+")

**Web of Science**
#15#14 AND #11 AND #10
#14#13 OR #12
#13TS=prescri*
#12TS=delay*
#11TS=antibiotic*
#10#9 OR #8 OR #7 OR #6 OR #5 OR #4 OR #3 OR #2 OR #1
#9TS="sore throat*"

#8TS=sinusitis
#7TS=bronchitis
#6TS="common cold"
#5TS=tonsillitis
#4TS=pharyngitis
#3TS="otitis media"
#2TS=urti
#1TS="Respiratory Tract Infection*"
