# Supplementary material for: Delayed antibiotic prescribing for respiratory tract infections: protocol of an individual patient data meta-analysis
Source: BMJ Open. 2019 Jan 21;9(1):e026925. doi: 10.1136/bmjopen-2018-026925 (PMC6347865; doi:10.1136/bmjopen-2018-026925)
Supplement: Author's manuscript [file bmjopen-2018-026925.draft_revisions.pdf]

BMJ Open is committed to open peer review. As part of this commitment we make the peer review history of every article we publish publicly available.

When an article is published we post the peer reviewers' comments and the authors' responses online. We also post the versions of the paper that were used during peer review. These are the versions that the peer review comments apply to.

The versions of the paper that follow are the versions that were submitted during the peer review process. They are not the versions of record or the final published versions. They should not be cited or distributed as the published version of this manuscript.

BMJ Open is an open access journal and the full, final, typeset and author-corrected version of record of the manuscript is available on our site with no access controls, subscription charges or pay-per-view fees (http://bmjopen.bmj.com).

If you have any questions on BMJ Open's open peer review process please email info.bmjopen@bmj.com

BMJ Open

Delayed antibiotic prescribing for respiratory tract infections: protocol of an individual patient data meta-analysis

Journal:	*BMJ Open*
Manuscript ID	bmjopen-2018-026925
Article Type:	Protocol
Date Submitted by the Author:	03-Oct-2018
Complete List of Authors:	Stuart, Beth; University of Southampton, Academic Unit of Primary Care and Population Sciences, Faculty of Medicine<br>Hounkpatin, Hilda; University of Southampton, Academic Unit of Primary Care and Population Sciences, Faculty of Medicine<br>Becque, Taeko; University of Southampton, Academic Unit of Primary Care and Population Sciences, Faculty of Medicine<br>Yao, Guiqing; University of Leicester, UK, Biostatistics Research Group, Department of Health Sciences, College of Life Sciences<br>Zhu, Shihua; University of Southampton, Academic Unit of Primary Care and Population Sciences, Faculty of Medicine<br>Alonso-Coello, Pablo; Iberoamerican Cochrane Centre, CIBERESP-IIB Sant Pau<br>Altiner, Attila; University of Rostock, Office for Educational Affairs, Department of General Medicine<br>Arroll, Bruce; University of Auckland, Department of General Practice and Primary Health Care<br>Böhning, Dankmar ; University of Southampton, Southampton Statistical Sciences Research Institute<br>Bostock, Jennifer; King's College London, Divison of Health and Social Care Research<br>Bucher, Heiner; University Hospital Basel and University of Basel, Basel Institute for Clinical Epidemiology and Biostatistics (CEB)<br>de la Poza, Mariam; Institut Català de la Salut<br>Francis, Nick A.; Cardiff University, Division of Population Medicine, School of Medicine<br>Gillespie, David<br>Hay, Alastair; University of Bristol, Centre for Academic Primary Care, Population Health Sciences<br>Kenealy, Tim; University of Auckland, General Practice and Primary Health Care<br>Löffler, Christin; Rostock University Medical Center, Institute of General Practice<br>Mas-Dalmau, Gemma; Hospital de la Santa Creu i Sant Pau, Knowledge and research management unit<br>Muñoz, Laura; Agència de Qualitat i Avaluació Sanitàries de Catalunya (AQuAS), Roc Boronat, 81-95, 08005, Barcelona, Spain<br>Samuel, Kirsty; University of Leeds, ASPIRE PPI Panel, Leeds, Institute for Health Sciences<br>Moore, Michael; University of Southampton, Academic Unit of Primary Care and Population Sciences, Faculty of Medicine

Little, Paul; University of Southampton, Academic Unit of Primary Care and Population Sciences, Faculty of Medicine

Keywords: RESPIRATORY MEDICINE (see Thoracic Medicine), STATISTICS & RESEARCH METHODS, HEALTH ECONOMICS, GENERAL MEDICINE (see Internal Medicine)

SCHOLARONE™
Manuscripts

For peer review only

Delayed antibiotic prescribing for respiratory tract infections: protocol of an individual patient data meta-analysis

Beth Stuart[1*], Hilda Hounkpatin[1*], Taeko Becque[1], Guiqing Yao[2], Shihua Zhu[1], Pablo Alonso-Coello[3], Attila Altiner[4], Bruce Arroll[5], Dankmar Böhning[6], Jennifer Bostock[7], Heiner C. Bucher[8], Mariam de la Poza[9], Nick Francis[10], David Gillespie[11], Alastair Hay[12], Timothy Kenealy[13], Christin Loffler[14], Gemma Mas-Dalmau[15], Laura Muñoz[16], Kirsty Samuel[17], Michael Moore[1] and Paul Little[1]

1. Academic Unit of Primary Care and Population Sciences, Faculty of Medicine, University of Southampton, Southampton, UK
2. Biostatistics Research Group, Department of Health Sciences, College of Life Sciences, University of Leicester, Leiceister, UK
3. Iberoamerican Cochrane Centre, CIBERESP-IIB Sant Pau, Barcelona, Spain
4. Office for Educational Affairs, Department of General Medicine, University of Rostock, 18057 Rostock, Germany
5. Department of General Practice and Primary Health Care, University of Auckland, Auckland, New Zealand
6. Southampton Statistical Sciences Research Institute, University of Southampton, Southampton, SO17 1BJ, UK
7. Division of Health and Social Care Research, King's College London, London, UK
8. Basel Institute for Clinical Epidemiology and Biostatistics (CEB), University Hospital Basel and University of Basel, Switzerland
9. Institut Català de la Salut. CAP Doctor Carles Ribas, Foc 112, Barcelona, Spain
10. Division of Population Medicine, School of Medicine, Cardiff University, Cardiff, South Glamorgan, UK
11. South East Wales Trials Unit (SEWTU), Institute for Translation, Innovation, Methodology and Engagement (TIME), School of Medicine, Cardiff University, UK
12. Centre for Academic Primary Care, Population Health Sciences, Bristol Medical School, University of Bristol, Bristol, UK
13. Department of General Practice and Primary Health Care, University of Auckland, Auckland, New Zealand.
14. Institute of General Practice, Rostock University Medical Centre, Rostock, Germany
15. Knowledge and research management unit, Hospital de la Santa Creu i Sant Pau, Barcelona, Spain
16. Agència de Qualitat i Avaluació Sanitàries de Catalunya (AQuAS), Barcelona, Spain
17. ASPIRE PPI Panel, Leeds, Institute for Health Sciences, University of Leeds, Leeds, UK

*Beth Stuart and Hilda Hounkpatin contributed equally to this paper.

**Corresponding author:
Dr Beth Stuart
Primary Care and Population Sciences Faculty of Medicine University of Southampton Aldermoor Health Centre Aldermoor Close Southampton SO16 5ST
Email: bls1@soton.ac.uk
Telephone number: +44 23 8059 1759
Fax: +44 23 8070 1125

Keywords: Respiratory medicine; Statistics and research methods; Health economics; General medicine.

Word count: 3920

For peer review only

Delayed antibiotic prescribing for respiratory tract infections: protocol of an individual patient data meta-analysis

ABSTRACT

Introduction

Delayed prescribing can be a useful strategy to reduce antibiotic prescribing, but it is not clear for whom delayed prescribing might be effective. This protocol outlines an individual patient data (IPD) meta-analysis of randomised control trials (RCTs) and observational cohort studies to explore the overall effect of delayed prescribing and identify key patient characteristics that are associated with efficacy of delayed prescribing.

Methods and analysis

A systematic search of the databases Cochrane Central Register of Controlled Trials (CENTRAL), Ovid MEDLINE, Ovid Embase, EBSCO CINAHL Plus and Web of Science was conducted to identify relevant studies from inception to October 2017. Outcomes of interest include duration of illness, severity of illness, complication, re-consultation and patient satisfaction. Study authors of eligible papers will be contacted and invited to contribute raw IPD data. IPD data will be checked against published data, harmonised and aggregated to create one large IPD database. Multilevel regression will be performed to explore interaction effects between treatment allocation and patient characteristics. The economic evaluation will be conducted based on individual patient data from the combined trial and observational studies to estimate the differences in costs and effectiveness for delayed prescribing compared to normal practice. A decision model will be developed to assess potential savings and cost-effectiveness in terms of reduced antibiotic usage of delayed prescribing and quality adjusted life years.

Ethics and dissemination

Ethical approval was obtained from the University of Southampton Faculty of Medicine Research Ethics Committee (Reference number: 30068). Findings of this study will be published in peer-reviewed academic journals as well as GP trade journals and will be presented at national and international conferences. The results will have important public health implications, shaping the way in which antibiotics are prescribed in the future and to whom delayed prescriptions are issued.

Word count: 3920

ARTICLE SUMMARY

Strengths and limitations of this study

- This study uses individual patient data (IPD) from randomised controlled trials and observational studies to investigate the clinical effectiveness and potential cost savings of a delayed antibiotic prescribing, both overall and for key subgroups of people.
- IPD provides sufficient statistical power to explore interactions between treatment groups and patient characteristics.
- The IPD will only include data from studies for which the data is available.
- Observational studies will use propensity score approach to adjust for confounding by indication on measured covariates.

For peer review only

- Findings from this study may inform GPs decisions on prescribing and increase use of appropriate delayed prescribing, which in turn can help to reduce antibiotic consumption and antimicrobial resistance.

INTRODUCTION

Antimicrobial resistance is a cause for great concern prompting calls for action at the local, national and international level to prevent “overuse, misuse and abuse” of antibiotics (1), particularly in primary care where antibiotics are most prescribed (2). About 60% of antibiotics prescribed in primary care are for respiratory tract infections (RTIs) (3). However, most infections are self-limiting, symptomatic benefit from antibiotics is modest (4-6) and patients prescribed antibiotics for RTIs are more likely to carry antibiotic resistant commensal bacteria and develop resistant infections (7).

The Standing Medical Advisory Committee report recommends that the fewest number of antibiotic courses should be prescribed for the shortest period possible (8). However, in a primary care context, it can be difficult to tell whether antibiotics are appropriate for an individual patient. It is not always clear which patients are at risk of prolonged illness or developing complications nor whether the RTI is bacterial or viral in nature. Point of care tests such as C reactive protein and clinical scores both show promise in helping to guide GP management decisions (9-12). Delayed prescribing is a useful strategy that may be used on its own or in conjunction with clinical scores and point of care testing. It allows the patient to collect a prescription to be taken if their symptoms do not start to improve within a specific duration of their initial consultation, providing a potential aid to negotiating treatment acceptable to the patient and provider whilst reducing inappropriate prescribing (13,14). Delayed prescribing is recognised as part of the toolkit available to GPs to help reduce antibiotic use, especially in the context of consultations where patients expect to receive an antibiotic prescription and is part of the NICE guidelines (3). A recent large prospective primary care cohort (MRC DESCARTE) demonstrated that delayed prescription was likely to be as effective as immediate antibiotic in reducing complications and more effective at reducing re-consultations (4).

Whilst in some situations delayed antibiotic prescribing is appropriate, for other patients it may be unsuitable and controversial (15). It is important to understand which subgroups of patients may require immediate antibiotics to avoid complications and which patients might benefit from a delayed or no prescribing strategy (4). A 2013 Cochrane review (16) of 10 trials found that a delayed prescribing strategy was not significantly different to immediate prescribing in terms of clinical outcomes for cough and cold. In patients with acute otitis media and sore throat immediate antibiotics were more effective than delayed for reducing fever, pain and malaise in some studies. However, the review noted a high level of heterogeneity between studies made combining them in a traditional meta-analysis difficult and did not allow sufficient power for the examination of subgroups or complications either in meta-analysis or in meta-regression.

Where aggregation is possible, a traditional meta-analysis or even meta-regression can be used to determine the overall main effect of delayed antibiotic prescription. Although such techniques can also be used to explore how effect varies across study characteristics, these techniques lack statistical power and are prone to confounding as patient-level characteristics are not taken into account (17). An individual patient data (IPD) meta-analysis is considered the gold standard for meta-analyses and can overcome the limitations of meta-analysis of aggregate data. In an IPD meta-analysis, original data for all participants in each study are obtained and synthesised. This allows sufficient statistical power (18) to explore interactions between treatment groups and patient characteristics. This would allow us to identify key groups in which delayed prescribing or no prescribing might be inadvisable due to longer durations of illness, greater severity of illness or higher risk of complications/re-consultation. These findings may inform GPs decisions on prescribing

For peer review only - http://bmjopen.bmj.com/site/about/guidelines.xhtml

and increase use of appropriate delayed prescribing, which in turn can help to reduce antibiotic consumption and antimicrobial resistance.

This protocol describes an IPD meta-analysis of both randomised controlled trials (RCTs) and observational cohort studies of delayed antibiotic prescribing for acute respiratory infections. Although RCTs represent the 'gold standard' design for assessing the impact of an intervention, the participants in these studies may differ systematically from the patients encountered in every day clinical practice due to the inclusion criteria. By including observational studies in a meta-analysis, with suitable techniques to control for potential confounding by indication, it may be possible to obtain better estimates of the effect of delayed prescribing in routine practice.

METHODS

Aims and objectives

This IPD study will investigate the clinical effectiveness and potential cost savings of a delayed antibiotic prescribing approach, both overall and for key subgroups of patients.

The objectives of this study are as follows:

- To achieve more accurate estimates of the clinical effectiveness of a delayed prescribing strategy on the primary outcome, symptom severity, by harmonising the outcomes from all trials and observational studies which have included this strategy.
- To estimate the clinical effectiveness of delayed prescribing for secondary outcomes: duration of illness, development of complications, re-consultation and patient satisfaction.
- To explore whether there are key subgroups (informed by the literature and described below) for whom delayed antibiotic prescribing may or may not be beneficial. Subgroups will be: prior duration of illness (above/below median for the condition), age (under 16 years, 16-64 years, age over 65 years), fever at baseline consultation (>37.5 C), comorbid conditions including lung comorbidity such as asthma or chronic obstructive pulmonary disease (COPD), and severity of symptoms at baseline consultation. The effectiveness of delayed antibiotic prescribing for subgroups of symptom complexes such as cough (acute bronchitis), pharyngitis, or otitis media will also be explored.
- To investigate whether symptom trajectories are influenced by antibiotic prescribing/ consumption.
- To estimate the costs of treatment of patients with respiratory tract infections (from the UK National Health Service (NHS) and Personal Social Services (PSS) perspective data) and investigate any potential cost savings and cost effectiveness in terms of antibiotic usage of delayed antibiotic prescriptions compared with antibiotic use.
- To identify priorities for future delayed prescribing research.

Study approach

A full systematic review will be conducted to identify and select eligible papers. Study authors of eligible papers will be contacted and invited to contribute raw data.

Systematic review to identify eligible papers

Eligibility criteria

Design - Randomized controlled trials or eligible observational cohort studies

For peer review only

Population – all patients attending primary, ambulatory or acute care settings with a respiratory tract infection

Intervention – delayed antibiotic prescription

Comparator – immediate antibiotic prescription or no antibiotic prescription

Outcomes – severity of illness (symptom severity), duration of illness, complication, re-consultation, patient satisfaction, costs and quality of life.

Search strategy for identification of studies
A systematic search of the databases Cochrane Central Register of Controlled Trials (CENTRAL), Ovid MEDLINE, Ovid Embase, EBSCO CINAHL Plus and Web of Science will be conducted to identify relevant papers published from inception to October 2017. The search strategy will be based on criteria set out by the Cochrane Collaboration in a recently published systematic review and is available in the online supplementary material appendix (19). ISRCTN registry will also be searched for any relevant trials. Literature search results will be exported to EndNote and then uploaded to COVIDENCE, a web-based software that facilitates collaboration among reviewers during the study selection process. Full text articles will be uploaded to COVIDENCE. Two reviewers (HH and TB) will independently assess articles to determine eligibility for inclusion and any discrepancies will be resolved by discussion or referred to a third reviewer (BS). Reference lists of included articles will be reviewed to identify other potential papers not retrieved in the initial search. Contributing authors and content experts will be asked if they have, or are aware of, any additional studies (published or unpublished).

Risk of bias assessment and certainty of evidence assessment
Two reviewers will independently assess the risk of bias of each included study. RCTs will be assessed using the Cochrane risk of bias tool that includes items on allocation bias (random sequence generation, allocation concealment, baseline imbalance), departures from intended interventions (participant and study personnel blinding, deviations from intended interventions and analysis in groups to which they were randomized), attrition bias and appropriate methods to account for missing data, detection bias (blinding of outcome assessors), and selective outcome reporting (20,21). The ROBINS-I tool will be used to assess quality of each observational study. The ROBINS-I tool contains items on bias due to confounding, selection bias, bias due to deviations from intended intervention, bias due to missing data and selective reporting (22). We will use GRADE to rate the overall certainty (quality) of evidence that includes the evaluation of risk of bias, inconsistency, indirectness, imprecision, and publication factors (23).

Data extraction and database creation
Authors of eligible trials and observational studies will be contacted via an email, or where this is not possible by a letter, outlining the study aims. They will be invited to collaborate and share their data in a format of their choice. A data sharing agreement may be provided on request. A complete database (containing data on all available measures) rather than key variables used in the publication will be requested. If resource usage and quality of life data were collected in separate forms, such information should be provided too. This will allow us to recalculate the primary outcomes where necessary to ensure a consistent approach has been used across studies with the aim of reducing the level of between-study heterogeneity. Collaborators will be able to provide the data in a format of their choice. Study data will be considered unavailable in the event that none of the authors have responded to multiple contact attempts or if all contacted study authors indicate they no longer have access to the data.

Data will be checked by comparing key variables (e.g. size of sample, descriptive statistics of demographic and outcome measures) with published data. We will aim to reproduce individual

For peer review only

study results to ensure the current analysis is consistent with what has been done in each study. Collaborators will be contacted if important discrepancies are identified and asked for clarification. The level of missing data within each included study will be assessed and discussed with study collaborators. If less than 5% of data is found to be missing on key baseline characteristics and outcome measures, the data will be analysed on a complete cases basis. However, if there is substantial missing data, the pattern and nature of the missingness will be explored and multiple imputation by chained equations (ICE) will be used if appropriate.

A copy of the data to be included in the IPD meta-analysis will be converted for analysis in STATA version 14 or above. Outcome measures from the different studies will be harmonised once data from all study authors have been received. In the case that coding for an outcome in a study differs greatly from other studies, it may be possible to simplify measures to allow the study to contribute data or exclude the study from certain analyses. Any difficulties with harmonisation will be discussed with the full collaborators group to decide on the most meaningful approach. Harmonised data from the different studies will be aggregated (using STATA) into a single large IPD database, with an indicator variable to identify patients from the same trial. The IPD database will be rechecked for accuracy.

The key resource information on medication, primary care consultation, hospital inpatients, hospital day cases, outpatient visits, accident and emergency (A&E) attendance from individual studies will be recorded if such information were collected and length of usage and frequency of attendance will be adjusted to the same time periods. We will use the UK national published tariff (BNF, National reference cost and PSSRU) to cost associated resource usage.

Analysis

Study and patient level characteristics will be described for all studies that contribute IPD. Characteristics of any studies that declined or were unable to provide data will be considered in order to determine the extent to which the included studies are a representative sample. A traditional meta-analysis may be performed, using data from published papers, to test for differences between studies included in the IPD meta-analysis and those that could not provide data (24).

Heterogeneity of eligible studies will be summarised using an I^2 statistic (tested by Higgins I-squared test). A substantial statistical heterogeneity will be considered if I^2 statistic is > 50% (21). Sources of heterogeneity will be explored and reported and a random effects model will be used for all analyses.

IPD meta-analysis

IPD meta-analysis will be conducted using a one-stage approach. The one-stage approach combines all the data in a single meta-analysis based on a regression model stratified by study (25). One stage analysis is often more appropriate for exploring treatment-covariate interactions as it has increased power and is less likely to suffer from aggregation bias (26). The aggregated IPD will be examined using multilevel regressions clustering on the individual study level to take into account any heterogeneity between studies. Using the aggregated IPD dataset, we will calculate the key outcome measures on a consistent basis. The model selected will be one that is appropriate to the outcome measure of interest. For example, assuming the underlying assumptions of the model are met, a linear regression will be used to model the severity of symptoms, a proportional-hazards model will be used to assess the duration of illness, or a suitable count model where time to event data is not available, and logistic regression models will be used for the complications/re-consultation outcome,

For peer review only

side effects and patient satisfaction. All models will control for baseline severity of illness and all individuals will be included on a modified intention to treat (ITT) basis (i.e. as randomised) (27).

To explore whether there are differences in treatment effects for certain sub-groups, we will consider 5 pre-specified subgroups which have been identified as potential effect modifiers by previous research: prior duration of illness (above/below median for the condition), age (under 16 years, 16-64 years, age over 65 years), fever at baseline consultation (>37.5 C), comorbid condition including lung comorbidity such as asthma or COPD, and severity of symptoms at baseline consultation. We will also explore differences according to the diagnostic group (acute sore throat, cough/chest infection, otalgia/otitis media, upper respiratory tract infection (URTI). The analyses described above will be repeated, testing for interactions between treatment allocation and sub-group characteristic.

Symptom trajectories in patients who do not take antibiotics will also be explored. For symptoms recorded in symptom diaries, we will calculate the median and inter quartile range of the reported time until all symptoms have settled completely, in those patients who did not receive an antibiotic prescription or who received a delayed prescription but did not fill it (where this data is available). As sensitivity analyses, we will also explore duration of moderately severe of symptoms.

Synthesis of observational studies will additionally use inverse probability weighting by propensity score analysis to adjust for confounding by indication on measured covariates. This serves to balance observational studies on key covariates) so that the distribution of baseline covariates is similar between treated and untreated subjects, making observational studies more like those in a RCT (28, 29). Potential covariates to be included in the propensity score include: demographic factors (e.g.: age, sex), comorbid health conditions and signs and symptoms at baseline consultation. Observational studies after this adjustment for potential confounding variables, will be included in the main analysis if appropriate. However, sensitivity analyses will explore whether the estimates from observational cohort studies differ from those found in the RCTs. If there is evidence of substantial differences, data from observational studies and RCTs will not be pooled in a single meta-analysis and reasons for these differences will be explored narratively.

Economic analysis

The economic evaluation will be taken from the NHS and PSS perspective. Outcomes will be differences in cost, costs per antibiotic prescription avoided, and costs per quality of life gained. Individual patient resource usage data such as medication use, GP consultations, outpatient visits, accident & emergency attendance and use of secondary care service from each individual trial or observational study will be costed using UK published data sources (British National Formulary (BNF), national reference costs and Personal Social Services Research Unit (PSSRU)). Total costs for individual patients during study periods will be calculated.

A decision analytic model will be developed to project the potential saving (due to reduced antibiotic usage, and reduced frequency of attending primary care consultation) and cost effectiveness in terms of reduced antibiotic usage, and quality adjusted life year gained of delayed antibiotics. The model will consider re-infection and present the results at different time lengths, up to a maximum of 5 years. The model will be an individual based micro-simulation taking into account individual risk profiles with associated risk equations. The model will simulate individual treatment pathways with defined baseline characteristics and will capture the time spent at different levels of disease severity, time to recovery and recurrence rates. The probabilities of staying or transitioning to another state will be derived from the statistical analyses. The clinical analyses based on all data will

For peer review only

provide more robust estimates to populate the input values for the data. The costs and cost effectiveness of subgroups will be explored in the decision model.

Quality of life questionnaire will be translated into utility scores based on the UK tariff appropriate to the instruments used in each study. The different instruments (such as EQ5D, SF6D or HUI) will be recorded and weighted in the aggregated analyses. Utility scores at each measurement point will be estimated and a mean utility score for symptom severity will be calculated. For studies where no quality of life data were collected, we will apply the utility scores estimated from the other studies according to symptom severity. Quality adjusted life years (QALYs) will be calculated based on an area under the curve approach. Due to the potentially skewed distribution in costs, a generalised linear mixed model will be employed to analyse costs and QALYs, controlling for baseline patient characteristics, length of follow-up and disease severity. The data will be hierarchical, with individual patients nested within studies, with study modelled as a random effect. Incremental costs and costs effectiveness and associated confidence intervals will be estimated by bootstrapping. The economic analyses will follow the same approach as the statistical analyses with calculations undertaken using a one-stage approach.

Sensitivity analyses

All analyses will be repeated using a two-step approach. The two-stage approach involves calculating the effect for each study separately and then combining the results using traditional meta- analysis techniques. Results using the one-stage and two-stage approach will be compared and any discrepancies explored. This will also allow us to include and explore the impact of the aggregate results from trials for which we are unable to obtain data, as aggregate results can only be included in a two-step approach. Aggregated data from observational studies will not be included because in the absence of full data, it will not be possible to control for confounding by indication.

Sensitivity analysis will explore whether there are differences in inferences based upon differences in the approach taken to delayed prescribing and whether there are any differences if studies at high risk of bias are excluded. We will also consider a higher cut off for presence of fever informed by the literature.

Given the potential long-term problem of antibiotic resistance, a sensitivity analysis will performed to incorporate the costs of antibiotic resistance in the economic analyses. However, given the difficulty previous researchers (30, 31) have had in estimating such costs we will vary the assumptions about the likely cost to see whether the inferences are modified.

Patient and Public Involvement (PPI)
The study has two PPI team members who have been actively involved in shaping the research questions and will continue to contribute to all stages of the research. Specifically, they will contribute to data harmonisation, providing advice to ensure the result will yield useful outcomes for patients and members of the public, interpretation of study findings and what they might mean for patients and the public, dissemination at relevant conferences/meetings and how best to disseminate findings to key patient and public groups. In return for their time, PPI representatives will be reimbursed in line with INVOLVE recommendations (32) and will be recognised as co-authors on research outputs.

Ethics and dissemination
Ethical approval for the study was obtained from the University of Southampton Faculty of Medicine Research Ethics Committee (Submission ID: 30068). There will be no identifiable patient data in any of the datasets and data will be stored in a password-restricted folder on the university server. The

For peer review only

protocol of this IPD meta-analysis has been registered with the International Prospective Register of Systematic Reviews (PROSPERO) at the National Health Service Centre for Reviews and Dissemination at the University of York (Registration number: CRD42018079400). This protocol has been prepared in accordance with the PRISMA-P guidelines (32).

The results of this work will provide the best possible evidence regarding when and for whom delayed prescribing is appropriate. The results, disseminated through academic and trade journals as well as conferences, should help GPs in their decision-making about prescribing during consultations. The results will also be shared with patients and members of the public through our Patient Public Involvement collaborations. There is potential for PPI to help address the issue of patients seeking antibiotics inappropriately, which may help reduce unnecessary prescribing and in turn, antimicrobial resistance.

DISCUSSION

IPD meta-analysis is more appropriate and has higher statistical power than traditional meta-analysis to identify which key subgroups or patient characteristics may or may not benefit from delayed prescribing. Another advantage of IPD is that some of the biases in studies can be explored more fully than in aggregate data meta-analysis. For example, it is possible to check the randomization integrity and to perform a full intention to treat analysis to minimize attrition bias, even if this was not performed for the original publication. Furthermore, with the collaboration and communication required for a successful IPD, it should be possible to obtain information from the original study authors about potential sources of bias. A further advantage of IPD meta-analysis is that it allows study outcomes to be calculated in the same way and the models adjusted for similar confounders, which can reduce heterogeneity across studies.

However, there are some challenges associated with IPD meta-analysis (33). The key risk is not obtaining all the relevant data and failure to do so can result in biased results. It may be particularly difficult to obtain data for older studies, as the data may have been lost or destroyed or the lead researcher may have retired or changed field. We therefore plan to undertake a sensitivity analysis using a two-step approach which will allow us to incorporate the published estimates from any trials for which we are unable to obtain IPD into a meta-analysis. Although IPD offers the ability to conduct additional and more accurate and appropriate analyses, in many cases similar results and conclusions can be drawn from IPD and standard meta-analysis (33). It is also possible that the outcome measures may not be sufficiently similar to harmonise. An initial scoping review suggest that almost all studies have collected the key outcome measures in a similar way. However, if it is deemed impossible to perform a meta-analysis with the data, a narrative review will be undertaken instead. We hope the results of the study will help GPs to communicate more effectively with patients about the normal course of illness, which patients are likely to benefit from a delayed prescribing approach, the costs of antibiotic use for RTIs, and help the general public to feel more confident about waiting to see whether their illness settles before visiting their GP.

Acknowledgements: We would like to thank the following collaborators which assisted us by allowing us to use the data from their studies: Dr Jennifer Chao, Dr David McCormick, Professor Ngaire Kerse, Ms Jacqueline Nuttall.

Author contributions: BS is the guarantor. BS conceived the original study concept and design and obtained funding from the NIHR Research for Patient Benefit (RfPB) Programme. BS and HH wrote the first draft of the manuscript. GY and SZ contributed to the section on health economics. All listed authors contributed to the concept and design of the study and manuscript editing. All authors read, provided feedback and approved the final manuscript.

For peer review only

Funding: This work was funded by the NIHR Research for Patient Benefit (RfPB) Programme, grant number: PB-PG-0416-20005. This funding will support the collation of the individual participant data, data management and analyses. The NIHR RfPB is not involved in any other aspect of the project, such as the design of the project's protocol and analysis plan, the collection and analyses. The funder will have no input on the interpretation or publication of the study results.

Data sharing statement: Search strategies can be found in the appendix.

Conflicts of interest: none declared.

References

1. Ridge KW, Hand K, Sharland M, Abubakar I, Livermore DM. Infections and the rise of antimicrobial resistance. London: Department of Health; 2011.
2. Public Health England. English surveillance programme for antimicrobial utilisation and resistance report. 2016.
3. National Institute for Health and Care Excellence. Prescribing of antibiotics for self-limiting respiratory tract infections in adults and children in primary care (Clinical Guideline 69); 2008.
4. Little P, Stuart B, Hobbs FD, Butler CC, Hay AD, Delaney B, et al. Antibiotic prescription strategies for acute sore throat: a prospective observational cohort study. The Lancet Infectious Diseases. 2014;14(3):213-9.
5. Spinks A, Glasziou PP, Del Mar CB. Antibiotics for sore throat. The Cochrane Database of Systematic Reviews. 2013;5(11):CD000023.
6. Kenealy T, Arroll B. Antibiotics for the common cold and acute purulent rhinitis. The Cochrane Database of Systematic Reviews. 2013; 4(6); CD000247
7. Costelloe C, Metcalfe C, Lovering A, Mant D, Hay AD. Effect of antibiotic prescribing in primary care on antimicrobial resistance in individual patients: systematic review and meta-analysis. BMJ (Clinical research ed). 2010;340:c2096.
8. Standing Medical Advisory Committee S-GoAR. The path of least resistance. 1998.
9. Cooke J, Butler C, Hopstaken R, Dryden MS, McNulty C, Hurding S, et al. Narrative review of primary care point-of-care testing (POCT) and antibacterial use in respiratory tract infection (RTI). BMJ open respiratory research. 2015;2(1):e000086.
10. Little P, Hobbs FD, Moore M, Mant D, Williamson I, McNulty C, et al. Clinical score and rapid antigen detection test to guide antibiotic use for sore throats: randomised controlled trial of PRISM (primary care streptococcal management). British Medical Journal (Clinical research ed). 2013;347:f5806.
11. Hay AD, Redmond NM, Turnbull S, Christensen H, Thornton H, Little P, et al. Development and internal validation of a clinical rule to improve antibiotic use in children presenting to primary care with acute respiratory tract infection and cough: a prognostic cohort study. The Lancet Respiratory Medicine. 2016;4(11):902-10.
12. Hay AD, Sterne JA, Hood K, Little P, Delaney B, Hollingworth W, et al. Improving the Diagnosis and Treatment of Urinary Tract Infection in Young Children in Primary Care: Results from the DUTY Prospective Diagnostic Cohort Study. Annals of Family Medicine. 2016;14(4):325-36.
13. Little P. Delayed prescribing of antibiotics for upper respiratory tract infection. British Medical Journal (Clinical research ed). 2005;331(7512):301–2.
14. Peters S, Rowbotham S, Chisholm A, Wearden A, Moschogianis S, Cordingley L, et al. Managing self-limiting respiratory tract infections: a qualitative study of the usefulness of the delayed prescribing strategy. The British Journal of General Practice : the Journal of the Royal College of General Practitioners. 2011;61(590):e579-89.
15. Ryves R, Eyles C, Moore M, McDermott L, Little P, Leydon GM. Understanding the delayed prescribing of antibiotics for respiratory tract infection in primary care: a qualitative analysis. BMJ Open. 2016;6(11):e011882.

For peer review only

16. Spurling GK, Del Mar CB, Dooley L, Foxlee R, Farley R. Delayed antibiotics for respiratory infections. The Cochrane Database of Systematic Reviews. 2013; 30(4):CD004417.
17. Berlin JA, Santanna J, Schmid CH, Szczech LA, Feldman HI. Individual patient- versus group-level data meta-regressions for the investigation of treatment effect modifiers: ecological bias rears its ugly head. Statistics in Medicine. 2002;21(3):371-87.
18. Lambert PC, Sutton AJ, Abrams KR, Jones DR. A comparison of summary patient-level covariates in meta-regression with individual patient data meta-analysis. Journal of Clinical Epidemiology. 2002;55(1):86-94.
19. Spurling GK, Del Mar CB, Dooley L, Foxlee R, Farley R. Delayed antibiotic prescriptions for respiratory infections. The Cochrane Database of Systematic Reviews. 2017;9:CD004417.
20. Savovic J, Page M, Elbers R, Hrobjartsson A, Boutron I, Reeves B, et al. A revised tool for assessing risk of bias in randomized trials (RoB 2.0). Trials Conference: 4th international clinical trials methodology conference , ICTMC and the 38th annual meeting of the society for clinical trials United kingdom. 2017;18(Supplement 1) (no pagination).
21. Higgins JPT, Green S, editors. Assessing risk of bias in included studies: The Cohrane Collaboration; 2011.
22. Sterne JA, Hernan MA, Reeves BC, Savovic J, Berkman ND, Viswanathan M, et al. ROBINS-I: a tool for assessing risk of bias in non-randomised studies of interventions. British Medical Journal (Clinical research ed). 2016;355:i4919.
23. Guyatt GH, Oxman AD, Akl EA, Vist G, Brozek J, Norris S, et al. GRADE guidelines: 1. Introduction-GRADE evidence profiles and summary of findings tables. Journal of Clinical Epidemiology. 2011;64(4): 383-94.
24. Riley RD, Simmonds MC, Look MP. Evidence synthesis combining individual patient data and aggregate data: a systematic review identified current practice and possible methods. Journal of Clinical Epidemiology. 2007;60(5):431-9.
25. Riley RD, Lambert PC, Abo-Zaid G. Meta-analysis of individual participant data: rationale, conduct, and reporting. British Medical Journal (Clinical research ed). 2010;340:c221.
26. Stewart GB, Altman DG, Askie LM, Duley L, Simmonds MC, Stewart LA. Statistical analysis of individual participant data meta-analyses: a comparison of methods and recommendations for practice. PloS One. 2012;7(10):e46042.
27. Akl EA, Kahale LA, Agoritsas T, Brignardello-Petersen R, Busse JW, Carrasco-Labra A, et al. Handling trial participants with missing outcome data when conducting a meta-analysis: a systematic survey of proposed approaches. Systematic Reviews. 2015;4:98.
28. Rosenbaum PR, Rubin DB. The central role of the propensity score in observational studies for causal effects. Biometrika. 1983;70:41-55.
29. D'Agostino RB, Jr. Propensity score methods for bias reduction in the comparison of a treatment to a non-randomized control group. Statistics in Medicine. 1998;17(19):2265-81.
30. Smith R, Coast J. The true cost of antimicrobial resistance. British Medical Journal. 2013;346.
31. Oppong R, Smith RD, Little P, Verheij T, Butler CC, Goossens H, et al. Cost effectiveness of amoxicillin for lower respiratory tract infections in primary care: an economic evaluation accounting for the cost of antimicrobial resistance. The British Journal of General Practice : the Journal of the Royal College of General Practitioners. 2016;66(650):e633-9.
31. Payment- What rates should be offered for involveent in research? National Health Research Involve website. http://www.invo.org.uk/posttypefaq/payment-what-rates-should-be-offered-for-involvement-in-research. Published 2013. Accessed September 11, 2018.
32. Shamseer L, Moher D, Clarke M, Ghersi D, Liberati A, Petticrew M, et al. Preferred reporting items for systematic review and meta-analysis protocols (PRISMA-P) 2015: elaboration and explanation. British Medical Journal (Clinical research ed). 2015;350:g7647.
33. Tudur Smith C, Marcucci M, Nolan SJ, Iorio A, Sudell M, Riley R, et al. Individual participant data meta-analyses compared with meta-analyses based on aggregate data. The Cochrane Database Systematic Reviews. 2016;9: MR000007.

Supplementary Online Appendix 1. CENTRAL, MEDLINE, Embase, CINAHL, and Web of Science search strategies for randomised controlled trials (November 2017)

Cochrane Central Register of Controlled Trials (CENTRAL)
#1 MeSH descriptor: [Respiratory Tract Infections] explode all trees
#2 ((upper next respiratory next tract next infection*) or URTI):ti,ab,kw
#3 MeSH descriptor: [Otitis Media] explode all trees
#4 (otitis next media):ti,ab,kw
#5 MeSH descriptor: [Pharyngitis] explode all trees
#6 pharyngitis:ti,ab,kw
#7 MeSH descriptor: [Tonsillitis] explode all trees
#8 tonsillitis:ti,ab,kw
#9 MeSH descriptor: [Common Cold] explode all trees
#10 (common next cold*):ti,ab,kw
#11 MeSH descriptor: [Bronchitis] explode all trees
#12 bronchitis:ti,ab,kw
#13 MeSH descriptor: [Sinusitis] explode all trees
#14 sinusitis:ti,ab,kw
#15 (sore next throat*):ti,ab,kw
#16 #1 or #2 or #3 or #4 or #5 or #6 or #7 or #8 or #9 or #10 or #11 or #12 or #13 or #14 or #15
#17 MeSH descriptor: [Anti-Bacterial Agents] explode all trees
#18 antibiotic*:ti,ab,kw
#19 #17 or #18
#20 (delay* near/15 prescri*):ti,ab,kw
#21 #16 and #19 and #20

Ovid MEDLINE
1 exp Respiratory Tract Infections/
2 (upper respiratory tract infection$ or urti).mp.
3 exp Otitis Media/
4 otitis media.mp.
5 exp Pharyngitis/
6 pharyngitis.mp.
7 exp Tonsillitis/
8 tonsillitis.mp.
9 exp Common Cold/
10 common cold.mp.
11 exp Bronchitis/
12 bronchitis.mp.
13 exp Sinusitis/
14 sinusitis.mp.
15 sore throat$.mp.
16 or/1-15
17 exp Anti-Bacterial Agents/
18 antibiotic$.mp.
19 or/17-18
20 (delay$ adj15 prescri$).mp.
21 16 and 19 and 20
22 (2013* or 2014* or 2015* or 2016* or 2017*).ed.
23 21 and 22

Ovid Embase
1 exp Respiratory Tract Infection/
2 exp Upper Respiratory Tract Infection/
3 (upper respiratory tract infection$ or urti).mp.
4 exp Otitis Media/
5 otitis media.mp.

For peer review only

6 exp Pharyngitis/
7 pharyngitis.mp.
8 exp Tonsillitis/
9 tonsillitis.mp.
10 exp Common Cold/
11 common cold.mp.
12 exp Bronchitis/
13 bronchitis.mp.
14 exp Sinusitis/
15 sinusitis.mp.
16 sore throat$.mp.
17 or/1-16
18 exp antibiotic agent/
19 antibiotic$.mp.
20 or/18-19
21 (delay$ adj15 prescri$).mp.
22 17 and 20 and 21
23 (2013* or 2014* or 2015* or 2016* or 2017*).em.
24 22 and 23

EBSCO CINAHL Plus
S15 S10 and S13 and S14
S14 TI delay* N15 prescri* or AB delay* N15 prescri*
S13 S11 or S12
S12 TI antibiotic* or AB antibiotic*
S11 (MH "Antibiotics+")
S10 S1 or S2 or S3 or S4 or S5 or S6 or S7 or S8 or S9
S9 TI (otitis media or pharyngitis or tonsillitis or common cold* or bronchitis or sinusitis or sore throat*) or AB (otitis media or pharyngitis or tonsillitis or common cold* or bronchitis or sinusitis or sore throat*)
S8 (MH "Sinusitis+")
S7 (MH "Bronchitis+")
S6 (MH "Common Cold")
S5 (MH "Tonsillitis+")
S4 (MH "Pharyngitis")
S3 (MH "Otitis Media+")
S2 TI (upper respiratory tract infection* or urti) or AB (upper respiratory tract infection* or urti)
S1 (MH "Respiratory Tract Infections+")

Web of Science
#15#14 AND #11 AND #10
#14#13 OR #12
#13TS=prescri*
#12TS=delay*
#11TS=antibiotic*
#10#9 OR #8 OR #7 OR #6 OR #5 OR #4 OR #3 OR #2 OR #1
#9TS="sore throat*"
#8TS=sinusitis
#7TS=bronchitis
#6TS="common cold"
#5TS=tonsillitis
#4TS=pharyngitis
#3TS="otitis media"
#2TS=urti
#1TS="Respiratory Tract Infection*"

For peer review only

Supplementary Online Appendix 2. CENTRAL, MEDLINE, Embase, CINAHL, and Web of Science search strategies for observational studies (November 2017)

Cochrane Central Register of Controlled Trials (CENTRAL)
#1 MeSH descriptor: [Respiratory Tract Infections] explode all trees
#2 ((upper next respiratory next tract next infection*) or URTI):ti,ab,kw
#3 MeSH descriptor: [Otitis Media] explode all trees
#4 (otitis next media):ti,ab,kw
#5 MeSH descriptor: [Pharyngitis] explode all trees
#6 pharyngitis:ti,ab,kw
#7 MeSH descriptor: [Tonsillitis] explode all trees
#8 tonsillitis:ti,ab,kw
#9 MeSH descriptor: [Common Cold] explode all trees
#10 (common next cold*):ti,ab,kw
#11 MeSH descriptor: [Bronchitis] explode all trees
#12 bronchitis:ti,ab,kw
#13 MeSH descriptor: [Sinusitis] explode all trees
#14 sinusitis:ti,ab,kw
#15 (sore next throat*):ti,ab,kw
#16 #1 or #2 or #3 or #4 or #5 or #6 or #7 or #8 or #9 or #10 or #11 or #12 or #13 or #14 or #15
#17 MeSH descriptor: [Anti-Bacterial Agents] explode all trees
#18 antibiotic*:ti,ab,kw
#19 #17 or #18
#20 (delay* near/15 prescri*):ti,ab,kw
#21 #16 and #19 and #20
#22 Epidemiologic Studies/
#23 exp Case-Control Studies/
#24 exp Cohort Studies/
#25 Cross-Sectional Studies/
#26 (epidemiologic adj (study or studies)).ab,ti.
#27 case control.ab,ti.
#28 (cohort adj (study or studies)).ab,ti.
#29 cross sectional.ab,ti.
#30 cohort analy$.ab,ti.
#31 (follow up adj (study or studies)).ab,ti.
#32 longitudinal.ab,ti.
#33 retrospective$.ab,ti.
#34 prospective$.ab,ti.
#35 (observ$ adj3 (study or studies)).ab,ti.
#36 adverse effect.ab,ti.
#37 #22 or #23 or #24 or #25 or #26 or #27 or #28 or #29 or #30 or #31 or #32 or #33 or #34 or #35 or #36
#38 #21 and #37

Ovid MEDLINE
1 exp Respiratory Tract Infections/
2 (upper respiratory tract infection$ or urti).mp.
3 exp Otitis Media/
4 otitis media.mp.
5 exp Pharyngitis/
6 pharyngitis.mp.
7 exp Tonsillitis/
8 tonsillitis.mp.
9 exp Common Cold/
10 common cold.mp.
11 exp Bronchitis/
12 bronchitis.mp.

For peer review only

13 exp Sinusitis/
14 sinusitis.mp.
15 sore throat$.mp.
16 or/1-15
17 exp Anti-Bacterial Agents/
18 antibiotic$.mp.
19 or/17-18
20 (delay$ adj15 prescri$).mp.
21 16 and 19 and 20
22 (2013* or 2014* or 2015* or 2016* or 2017*).ed.
23 21 and 22
24 Epidemiologic studies/
25 Exp case control studies/
26 Exp cohort studies/
27 Cross-sectional studies/
28 Case control.tw.
29 (cohort adj (study or studies or analys*)).tw.
30 Cohort analy$.tw.
31 ((Follow up or observational or uncontrolled or non randomi#ed or nonrandomi#ed or epidemiologic*) adj (study or studies)).tw
32 (longitudinal or retrospective or prospective or cross sectional) adj (study or studies or review or analys* or cohort*)).tw.
33 Longitudinal.tw.
34 Retrospective*.tw.
35 Prospective*.tw
36 Cross sectional.tw.
37 Follow-Up Studies/
38 correlational study.mp
39 Or/24-38
40 23 and 39

Ovid Embase
1 exp Respiratory Tract Infection/
2 exp Upper Respiratory Tract Infection/
3 (upper respiratory tract infection$ or urti).mp.
4 exp Otitis Media/
5 otitis media.mp.
6 exp Pharyngitis/
7 pharyngitis.mp.
8 exp Tonsillitis/
9 tonsillitis.mp.
10 exp Common Cold/
11 common cold.mp.
12 exp Bronchitis/
13 bronchitis.mp.
14 exp Sinusitis/
15 sinusitis.mp.
16 sore throat$.mp.
17 or/1-16
18 exp antibiotic agent/
19 antibiotic$.mp.
20 or/18-19
21 (delay$ adj15 prescri$).mp.
22 17 and 20 and 21
23 (2013* or 2014* or 2015* or 2016* or 2017*).em.
24 22 and 23

For peer review only

25 Clinical study/
26 exp Case control study/
27 Family study/
28 Longitudinal study/
29 Retrospective study/
30 Prospective study/
31 Randomized controlled trials/
32 30 not 31
33 Cohort analysis/
34 (Cohort adj (study or studies)).mp.
35 (Case control adj (study or studies)).tw.
36 ((Follow up or observational or uncontrolled or non randomi#ed or nonrandomi#ed or epidemiologic*) adj (study or studies)).tw
37 (longitudinal or retrospective or prospective or cross sectional) adj (study or studies or review or analys* or cohort*)).tw.
38 Follow-up/
39 38 and 33
40 correlational study.mp
41 Or/25-30,32-37,39-40
42 24 and 41

EBSCO CINAHL Plus
S24 S15 and S23
S23 or/S16-S22
S22 (observational adj (study or studies)).tw.
S21 (cohort adj (study or studies)).tw.
S20 Cross sectional studies/
S19 Nonconcurrent prospective studies/
S18 Correlational studies/
S17 Exp case control studies/
S16 Prospective studies/
S15 S10 and S13 and S14
S14 TI delay* N15 prescri* or AB delay* N15 prescri*
S13 S11 or S12
S12 TI antibiotic* or AB antibiotic*
S11 (MH "Antibiotics+")
S10 S1 or S2 or S3 or S4 or S5 or S6 or S7 or S8 or S9
S9 TI (otitis media or pharyngitis or tonsillitis or common cold* or bronchitis or sinusitis or sore throat*) or AB (otitis media or pharyngitis or tonsillitis or common cold* or bronchitis or sinusitis or sore throat*)
S8 (MH "Sinusitis+")
S7 (MH "Bronchitis+")
S6 (MH "Common Cold")
S5 (MH "Tonsillitis+")
S4 (MH "Pharyngitis")
S3 (MH "Otitis Media+")
S2 TI (upper respiratory tract infection* or urti) or AB (upper respiratory tract infection* or urti)
S1 (MH "Respiratory Tract Infections+")

Web of Science
#15#14 AND #11 AND #10
#14#13 OR #12
#13TS=prescri*
#12TS=delay*
#11TS=antibiotic*
#10#9 OR #8 OR #7 OR #6 OR #5 OR #4 OR #3 OR #2 OR #1
#9TS="sore throat*"

For peer review only

#8TS=sinusitis
#7TS=bronchitis
#6TS="common cold"
#5TS=tonsillitis
#4TS=pharyngitis
#3TS="otitis media"
#2TS=urti
#1TS="Respiratory Tract Infection*"

For peer review only

BMJ 2015;349:g7647 doi: 10.1136/bmj.g7647 (Published 2 January 2015)

Preferred reporting items for systematic review and meta-analysis protocols (PRISMA-P) 2015: elaboration and explanation

OPEN ACCESS

For peer review only

Larissa Shamseer [1], David Moher [1], Mike Clarke [2], Davina Ghersi [3], Alessandro Liberati (deceased) [4], Mark Petticrew [5], Paul Shekelle [6], Lesley A Stewart [7], the PRISMA-P Group

[1]Ottawa Hospital Research Institute and University of Ottawa, Canada; [2]Queen's University Belfast, Ireland; [3]National Health and Medical Research Council, Australia; [4]University of Modena, Italy; [5]London School of Hygiene and Tropical Medicine, UK; [6]Southern California Evidence-based Practice Center, USA; [7]Centre for Reviews and Dissemination, University of York, UK

Dedication: The PRISMA-P 2015 initiative is dedicated to our colleague Alessandro Liberati (1954–2012), who passed away while PRISMA-P 2015 was under development and whose contributions to this work were invaluable.

Abstract

Protocols of systematic reviews and meta-analyses allow for planning and documentation of review methods, act as a guard against arbitrary decision making during review conduct, enable readers to assess for the presence of selective reporting against completed reviews, and, when made publicly available, reduce duplication of efforts and potentially prompt collaboration. Evidence documenting the existence of selective reporting and excessive duplication of reviews on the same or similar topics is accumulating and many calls have been made in support of the documentation and public availability of review protocols. Several efforts have emerged in recent years to rectify these problems, including development of an international register for prospective reviews (PROSPERO) and launch of the first open access journal dedicated to the exclusive publication of systematic review products, including protocols (BioMed Central's *Systematic Reviews*). Furthering these efforts and building on the PRISMA (Preferred Reporting Items for Systematic Reviews and Meta-analyses) guidelines, an international group of experts has created a guideline to improve the transparency, accuracy, completeness, and frequency of documented systematic review and meta-analysis protocols—PRISMA-P (for protocols) 2015. The PRISMA-P checklist contains 17 items considered to be essential and minimum components of a systematic review or meta-analysis protocol.

This PRISMA-P 2015 Explanation and Elaboration paper provides readers with a full understanding of and evidence about the necessity of each item as well as a model example from an existing published protocol. This paper should be read together with the PRISMA-P 2015 statement. Systematic review authors and assessors are strongly encouraged to make use of PRISMA-P when drafting and appraising review protocols.

Introduction

Systematic reviews hold a unique place in healthcare. They help form the basis for developing practice guidelines and they provide information on gaps in knowledge, thus informing future research efforts. This information is relevant to stakeholders across the health system. The rigour and trustworthiness of systematic reviews is, in large part, based on the a priori planning and documentation of a methodical approach to conduct (that is, a protocol).

A systematic review protocol is important for several reasons: (1) it allows systematic reviewers to plan carefully and thereby anticipate potential problems; (2) it allows reviewers to explicitly document what is planned before they start their review, enabling others to compare the protocol and the completed review (that is, to identify selective reporting), to replicate review methods if desired, and to judge the validity of planned methods; (3) it prevents arbitrary decision making with respect to inclusion criteria and extraction of data; and (4) it may reduce duplication of efforts and enhance collaboration, when available.

Various international organizations such as the Cochrane and Campbell Collaborations and the Agency for Healthcare Research and Quality (AHRQ) regularly require and publish protocols. However, outside of such organizations, few protocols are published in traditional journals and most reports of completed reviews (89%) do not mention working from a protocol[1] (2014 update under way). Many experts have called for improved documentation and availability of review protocols. In response, experts (some of whom are authors on this document) launched an international, prospective register for systematic review protocols (PROSPERO, www.crd.york.ac.

Correspondence to: L Shamseer lshamseer@ohri.ca

For peer review only - http://bmjopen.bmj.com/site/about/guidelines.xhtml

No commercial reuse: See rights and reprints http://www.bmj.com/permissions Subscribe: http://www.bmj.com/subscribe

uk/prospero/) through the Centre for Reviews and Dissemination at the University of York (UK) in February 2011, in which more than 5000 systematic review protocols from 69 countries have been registered as of December 2014. In February 2012, the first open access journal to exclusively publish systematic review products including protocols (BioMed Central's *Systematic Reviews*) was launched, in which 142 protocols have been published (June 2014). Outside of select systematic review organizations, little to no general guidance exists for preparing review protocols.

Selective reporting

Arguably one of the most important functions of systematic review protocols is their role as a documentation of planned review methods, outcomes, and analyses that can be compared with completed reviews to detect whether unintended and undocumented changes were made. Bias related to selective reporting of outcomes (that is, when reporting is related to the statistical significance or direction of effect estimate) is a problem in clinical research. This is a well documented phenomenon in clinical trials,[2-7] and similar findings are starting to emerge for systematic reviews (see item 13 for full discussion).[8-10] When reviewers selectively choose which information to include in a report based on the direction and significance of findings, they risk biasing the evidence base on which healthcare decisions and policies are made.

Further to recent efforts to increase the documentation and availability of review protocols, the next logical step is the development of a set of standards that should be included in a review protocol. A well described protocol may facilitate and enhance the detection of undocumented changes to review methodology; it also may allow readers to gauge the potential impact of such changes as well as selective reporting of information on review findings.

To that end, a reporting guideline for systematic review protocols, an extension of the PRISMA (Preferred Items for Reporting Systematic Reviews and Meta-analyses) statement has been developed for protocols (PRISMA-P) and is described in detail in this paper.

Scope of PRISMA-P

PRISMA-P is intended to guide the development of protocols of systematic reviews and meta-analyses evaluating therapeutic efficacy. Even for systematic reviews that are not evaluating efficacy, authors are encouraged to use PRISMA-P because of the lack of existing protocol guidance overall. For the purpose of this guidance, we define a protocol, broadly, as a document written before the start of a systematic review describing the rationale and intended purpose of the review, and the planned methodological and analytical approach (see box 1 for comprehensive definitions).

PRISMA-P is meant to be used primarily by authors preparing systematic review protocols for publication, public consumption, or otherwise. It is also intended for those commissioning and potentially funding reviews as a guide for applicants on what should they should include in their review protocols, and as a tool for peer reviewers to gauge whether a protocol contains essential details. PRISMA-P will also be helpful for journal editors and peer reviewers gauging the adequacy of review protocols for publication. A list of stakeholders to whom we believe PRISMA-P will be useful along with proposed benefits for each group is provided in table 1⇓.

Development of PRISMA-P

The PRISMA-P checklist is based on elements from the PROSPERO register,[11] the PRISMA checklist,[12] SPIRIT (Standard Protocol Items: Recommendations for Interventional Trials) checklist items,[13] and Standard 2.6 from the Institute of Medicine's Standards for Systematic Reviews.[14] A detailed description of the steps undertaken during PRISMA-P development can be found in the PRISMA-P Statement paper.[15] The process follows general recommendations of the EQUATOR (Enhancing the Quality and Transparency of health Research) Network on how to develop a reporting guideline, of which one fundamental part is a consensus process.[16] An in-person consensus meeting of international experts was held in June 2011 in Rockville, MD, USA, to develop and refine PRISMA-P checklist items. All related guidance documents have undergone iterative revision within the PRISMA-P Group listed at the end of this document; members of the PRISMA-P Group contributed to the writing and identifying relevant examples in this document.

PRISMA-P checklist

The final PRISMA-P checklist contains 17 numbered items (26 sub-items) that should be described, at minimum, in protocols of systematic reviews and meta-analyses (table 2⇓). The checklist is divided into three main sections: administrative information, introduction, and methods. Readers familiar with PRISMA will observe that wording of the PRISMA-P checklists has, where possible, been harmonized with PRISMA checklist items, at least 13 of which are overlapping with PRISMA-P. We anticipate this will aid authors in transitioning their systematic review protocols prepared in accordance with PRISMA-P into full text, PRISMA-compliant, systematic review reports.

PRISMA-P Elaboration and Explanation

The format of this document follows that of previously established reporting guidelines such as the PRISMA Explanation and Elaboration document[17]; it aims to provide readers with comprehensive explanations and evidence based rationales for each checklist item. Examples of good reporting for each checklist item have been identified from existing systematic review and meta-analysis protocols and are provided throughout this document to enhance reader understanding of items.

Although PRISMA-P focuses on a minimal list of items to consider when preparing a systematic review protocol, we have indicated instances where additional information may be desirable to improve transparency of the planned review process. The recommendations within PRISMA-P may require more words or space than authors are accustomed to. Providing detailed descriptions for some protocol elements (such as item 8, eligibility criteria; item 13, outcomes and prioritisation) will facilitate transparency and future reproducibility, and allow authors to shorten their methods section in a completed systematic review report, if desired, by providing a brief summary of the methods and referring readers to the completed protocol or PROSPERO record. We believe that providing in depth descriptions of planned methodological details for systematic reviews is in line with emerging journal policies aimed at facilitating reproducibility.[18]

Checklist items are numbered as we envision them appearing in a protocol, and reporting them in this sequential order is a suggestion that may facilitate reader comprehension. Authors

For peer review only - http://bmjopen.bmj.com/site/about/guidelines.xhtml

No commercial reuse: See rights and reprints http://www.bmj.com/permissions
Subscribe: http://www.bmj.com/subscribe

Box 1: PRISMA-P terminology

Systematic review—A systematic review attempts to collate all relevant evidence that fits pre-specified eligibility criteria to answer a specific research question. It uses explicit, systematic methods to minimize bias in the identification, selection, synthesis, and summary of studies. When done well, this provides reliable findings from which conclusions can be drawn and decisions made.[179 180] The key characteristics of a systematic review are: (*a*) a clearly stated set of objectives with an explicit, reproducible methodology; (*b*) a systematic search that attempts to identify all studies that would meet the eligibility criteria; (*c*) an assessment of the validity of the findings of the included studies (such as assessment of risk of bias and confidence in cumulative estimates); and (*d*) systematic presentation, and synthesis, of the characteristics and findings of the included studies.

Meta-analysis—Meta-analysis is the use of statistical techniques to combine and summarize the results of multiple studies; they may or may not be contained within a systematic review. By combining data from several studies, meta-analyses can provide more precise estimates of the effects of healthcare than those derived from the individual studies.

Systematic review protocol—In the context of systematic reviews and meta-analyses, a protocol is a document that presents an explicit scientific "road map" of a planned, uninitiated systematic review. The protocol details the rational and planned methodological and analytical approach of the review.

should amend the order of appearance of checklist items if they deem it to be necessary. Most important is that authors describe each PRISMA-P item somewhere in their protocol.

One point to note is that, while the development of a protocol abstract is not a listed requirement on the PRISMA-P checklist, authors are urged to consult the PRISMA extension for reporting conference and journal abstracts if so desired.[19] The examples and explanations for each checklist item follow; citations contained within examples have been removed to avoid potential confusion with citations in this article.

Section 1: Administrative information

Title

Item 1a: Identification. Identify the report as a protocol of a systematic review

Example

"Postoperative outcomes following preoperative inspiratory muscle training in patients undergoing open cardiothoracic or upper abdominal surgery: protocol for a systematic review"[20]

Explanation

The knowledge in systematic reviews can be harnessed only if readers can easily identify them. Data indicate that systematic reviews are not always described as such in either the title or abstract; only 50% of systematic reviews included in a November 2004 sample used the terms "systematic review" or "meta-analysis" in their title or abstract.[1] Similar results have been reported elsewhere.[21] When this happens, reviews and meta-analyses may not be indexed in databases appropriately and risk not being found by potential users. This can lead to wasted efforts by systematic reviewers when knowledge they produce cannot be identified, one consequence of which may be unnecessary duplication of efforts by future reviewers.

Authors should title their report as a protocol of a systematic review and planned meta-analysis (the latter, only if known at the protocol stage). The term protocol indicates the existence of a plan for an upcoming, ongoing, or existing systematic review. Identification as a protocol may reduce unnecessary redundancy of systematic review efforts[22] and may also be helpful for readers seeking assistance in the design of future reviews. Although sensitive search strategies have been developed to identify systematic reviews,[23] inclusion of the terms systematic review or, if a meta-analysis is planned, meta-analysis in the title of a protocol may improve identification and retrieval.

We advise authors to use informative titles that make key information easily accessible to readers. Ideally, a title reflecting the PICO approach (participants, interventions, comparators, and outcomes) as well as time frame, setting, and study design, if desired (see Item 7), will provide readers with key information about the scope of the planned review.

Item 1b: Update. If the protocol is for an update of a previous systematic review, identify as such

Example

"The association between proximity to animal-feeding operations and community health: a protocol for updating a systematic review"[24]

Explanation

As explained in item 1a, authors can help to ensure awareness of the existence of a systematic review and review protocol by indicating this information in their title. Similar transparency will help readers identify whether the protocol in question is for conducting a new systematic review or an update of an existing one; ideally, this information should be reported within the title. Updates and, sometimes, expansions of an existing systematic review allow for the consideration of new evidence to bring previously published systematic reviews up to date.[25] Updating systematic reviews and identifying methods and signals for when to do so are increasingly being studied,[26-30] given that out of date systematic review evidence can be harmful,[31] particularly when updates yield changes in the direction of effect of one or more outcomes. Although systematic review updates are not always published as full length articles, they warrant an independent publication, the title of which should reflect its purpose.

Registration

Item 2. If registered, provide the name of the registry (such as PROSPERO) and registration number

Example

"In accordance with the guidelines, our systematic review protocol was registered with the International Prospective Register of Systematic Reviews (PROSPERO) on 11 July 2011 and was last updated on 19 January, 2012 (registration number CRD42011001410)."[32]

Explanation

Registration of systematic review protocol details is now recognized as desirable in order to promote and maintain transparency in the systematic review process, to assist in minimizing the risk of bias(es), and help to reduce unnecessary duplication of reviews.[33] At the time of publication, only one registry for prospective systematic review registration exists—the PROSPERO register (www.crd.york.ac.uk/prospero/). The PROSPERO register provides review authors with the

For peer review only - http://bmjopen.bmj.com/site/about/guidelines.xhtml

opportunity to freely register reviews evaluating interventions and strategies to prevent, diagnose, treat, and monitor conditions for which there is a health related outcome.[34 35] Since October 2013, key details from new protocols published in the *Cochrane Library* have been automatically added to PROSPERO on a daily basis. Future plans for PROSPERO include broadening inclusion to all systematic reviews with a health related outcome in the broadest sense (such as reviews of risk factors and genetic associations).

PROSPERO contains 22 mandatory items and 18 optional fields to capture key review attributes. However, it does not capture all information that should be included in a review protocol and does not preclude documentation and publication of a full review protocol. For easy transition from a registry entry into a full review protocol, many PRISMA-P items are based on PROSPERO items.

As with the preparation of a review protocol, the process of review registration forces authors to think through review methods and hopefully avoid future changes which may be associated with reporting biases. Furthermore, the registry entry itself provides readers with a reference to compare against complete reviews, in the absence of an available protocol, to examine for reporting biases. Logically, the planning, conduct, and reporting of reviews should involve efforts to help detect and minimize such bias.[10 36] Registration helps by prospectively recording key features of the planned review when the protocol has been finalized but before any eligibility screening has started, and making this information available publically and freely. This information provides those contemplating commissioning or undertaking a review to identify whether a relevant review is already planned or underway, if not completed. This should help avoid unplanned duplication, ensuring efficient use of resources and offering potential for future collaboration.[37 38] Of 73 randomly selected systematic reviews of randomised trials published in 2010, 49 (67%) had at least one overlapping meta-analysis that did not represent an update (that is, same comparison, type of population or indication, and outcome).[37] This signals a potentially large degree of wasted efforts.

Details and justification of any changes or amendments (see Item 4) made during the review process should be added to the registration record and reported in the final systematic review results report. By registering this information, the opportunity for post hoc manipulation and potential consequent bias are likely minimized. The public record allows comparison of published review results with what was planned so that readers can judge whether any discrepancies are likely to have introduced bias.

Registration information is increasingly being asked for by a number of journals as part of their submission process.[33 39 40] Once reviews are registered on PROSPERO, authors receive a unique identification number that authors should report in a review protocol, and in all publications arising from a review (that is, the protocol and completed review); doing so ensures that they can easily and confidently be identified as related.

Authors

Item 3a: Contact information. Provide name, institutional affiliation, and email address of all protocol authors; provide physical mailing address of corresponding author

Example

"*Corresponding author: Frances C Hillier
frances.hillier@durham.ac.uk

Author Affiliations

1 Department of Geography, Wolfson Research Institute, Durham University Queen's Campus, University Boulevard, Stockton-on-Tees, TS17 6BH, UK

2 Obesity Related Behaviours Research Group, School of Medicine and Health, Wolfson Research Institute, Durham University Queen's Campus, University Boulevard, Stockton-on-Tees, TS17 6BH, UK

Email: Clare L Bambra clare.bambra@durham.ac.uk - Frances C Hillier frances.hillier@durham.ac.uk - Helen J Moore helen.moore@durham.ac.uk - Carolyn D Summerbell carolyn.summerbell@durham.ac.uk"[41]

Explanation

Individuals who have made substantive intellectual contributions to the development of the systematic review protocol should provide their names, affiliations, and contact information even if the protocol is not published or intended to be published. Together with contributorship (Item 3b), this information can help identify competing interests and ghost authorship[42] and enhance the recognition and accountability of protocol authors and transparency of the review.[43] Although ghost authorship itself may not necessarily contribute to scientific bias, it may reflect the undisclosed shaping role played by companies or other groups with vested interests in the design or reporting of a study.[42 44-46]

In some instances, because of the nature of a relationship with a funder or sensitivity of the potential data, reviewers may not wish to have their names on a protocol before the systematic review is completed. In these instances, reviewers should provide contact information for the sponsor (host institution or funder) or for an individual assigned to deal with reader queries.

Item 3b: Contributions. Describe contributions of protocol authors and identify the guarantor of the review

Example

"DF is the guarantor. JE, RR and DM drafted the manuscript. All authors contributed to the development of the selection criteria, the risk of bias assessment strategy and data extraction criteria. SB developed the search strategy. RR provided statistical expertise. DF provided expertise on venous thromboembolism. SJ contributed to the section on health economics. All authors read, provided feedback and approved the final manuscript."[47]

Explanation

Some journals urge that published articles include descriptions of the contributions of each named author.[43 48] Likewise, in review protocols, together with names and contact information, the role(s) of each author should be clearly described. In biomedical publishing, journals require authors to have contributed to an article in at least the following ways: (1) contributed substantially to the conception and design of the study, the acquisition of data, or the analysis and interpretation; (2) drafted or provided critical revision of the article; and (3) provided final approval of the version to be published.[49]

For peer review only - http://bmjopen.bmj.com/site/about/guidelines.xhtml
For peer review only

The guarantor of a research article is the author who assumes the overall responsibility for the scientific integrity of the work as a whole and should be identified as such.[46 49] The term corresponding author typically represents the notion of "guarantor," and is also used to indicate which co-author is responsible for pre- and post-acceptance communication with the publishing journal and for taking queries to all other co-authors. A guarantor should be able to answer queries about the order of authors on the manuscript and about the research itself.[49] The guarantor is often listed as either the first named or most senior (often last) author.

Amendments

Item 4 If the report represents an amendment of a previously completed or published protocol, identify as such and indicate what changes were made; otherwise state plan for documenting important protocol amendments

Example 1

"In the event of protocol amendments, the date of each amendment will be accompanied by a description of the change and the rationale."[50]

Example 2

"If we need to amend this protocol, we will give the date of each amendment, describe the change and give the rationale in this section. Changes will not be incorporated into the protocol."[51]

Explanation

Systematic review protocols are typically iterative documents; modifications to protocols before and during the review process are to be expected. Systematic reviewers should give careful consideration to a review's methodological and analytical approach early on to avoid unnecessary changes after protocol development. A study of trials funded by pharmaceutical companies indicate that at least a third of amendments made to original trial protocols could have been prevented if key issues were given more consideration during protocol development[52]; this is likely true for systematic reviews as well. A 2002 study of 66 Cochrane reviews found that 91% of completed reviews had major changes from the protocol.[36] More recently, at least 20% of Cochrane reviews have been found to make post-protocol modifications to review outcomes (that is, addition, removal, or reprioritization), many of which are based on significance of the outcome in the completed review. Making changes to review outcomes, after knowledge of findings from included studies can introduce bias into the review process, mislead readers and possibly affect patient care. Cochrane reviews have since evolved to provide a dedicated section in which authors should report any changes made from the documented protocol.[53] Likewise, inclusion of a table summarizing protocol amendments is a mandatory requirement for reviews produced by AHRQ's Effective Health Care Program (table 3⇓). The PROSPERO register also allows for and tracks amendments of registered protocols.

Although many amendments do not introduce bias, changes from earlier protocol versions or from the registry entry should be transparently identified as such in each documented version of the protocol so that, at minimum, readers can evaluate the potential for bias. For protocols in which no amendments have yet been made, authors should include a description of the process for dealing with and documenting future amendments (that is, who will ultimately be responsible for approving, documenting, and implementing them). An updated protocol should be identified with a new version number and a list of specific amendments that were made to the previous version (see table 3⇓).

Support

Item 5a: Sources. Indicate sources of financial or other support for the review

Example

"This systematic review is funded by the Institute for Neurosciences, Mental Health and Addition, Canadian Institutes of Health Research (funding reference number KSD-115551; Effectiveness of the Screening, Brief Intervention and Referral to Treatment (SBIRT) Model for Reducing Illicit Drug Use: A Systematic Review)."[54]

Explanation

An updated Cochrane review indicates that drug trials funded by the pharmaceutical industry report significantly greater benefits, fewer harms, and more favourable overall conclusions than those with non-industry funding.[55 56] This issue, termed sponsorship bias, has been characterized less frequently in systematic reviews and meta-analyses. Of note, since 2004 the Cochrane Collaboration has prohibited industry support for its reviews.[57] One study indicates that conclusions from company supported reviews (2003, issue 1) recommended a drug not recommended in a matching, non-industry funded Cochrane review, despite both reviews having similar treatment effects; Cochrane reviews also had greater methodological transparency.[58] Another study of 124 meta-analyses found that meta-analyses with financial ties to one pharmaceutical company (n=49) were associated with more favourable conclusions, yet not more favourable results, than those with other financial ties.[59] Another study failed to replicate these findings, but it did find that industry supported meta-analyses have worse methodological quality than meta-analyses supported by non-profit organizations or unsupported meta-analyses.[60]

Review authors should disclose sources of financial and non-financial support for their review, if known at the protocol stage. If a review is not funded at the time the protocol is first registered and made available, the proposed sources of support should be listed and updated once funding is confirmed. Along with Item 5c (role of funder or sponsor), this information will help readers assess whether any competing interests or potential influences are present. As an example, the evaluation of sugar sweetened beverages and weight gain has recently received much attention for their purported association with negative health outcomes. A systematic review of reviews of sugar sweetened beverages and weight gain found that reviews identified as being affiliated with or supported by the food industry were five times more likely to report no positive, significant association with weight gain than non-industry affiliated reviews.[61] This finding highlights a need for authors to disclose their affiliations and sources of funding. Inclusion of the "financial conflicts of interest checklist 2010" with a protocol is recommended to help readers identify potential conflicts to be aware of; many journals have already instituted its use.[62]

Non-financial sources of support that should be disclosed may include the provision of services by an institution or funder, an information specialist who will help to obtain articles, access to a commercial database not otherwise available to reviewers, or in-kind use of software to manage or analyze review data.

For peer review only - http://bmjopen.bmj.com/site/about/guidelines.xhtml

Item 5b: Sponsor. Provide name of the review funder and/or sponsor

Example 1

"The Chartered Society of Physiotherapy Charitable Trust funded this research."[63]

Example 2

"The Laboratory of Research and Clinical Applications in Ophthalmology (Aristotle University of Thessaloniki) is the Sponsor, meaning that it has overall control of the data. No funding has been received for this study."[64]

Explanation

The term "sponsor" is most often associated with clinical trials in reference to the individual, company, institution, or organization assuming overall responsibility for the initiation and management of the trial.[65] However, because systematic reviews are often commissioned and funded by large agencies or companies, it is important for protocol authors to name both the sponsor and funder (Item 5a) in the review protocol, if applicable. The sponsor may not necessarily refer to the main funder if, for instance, a funder provides monies to a third party (sponsor) to carry out the research. This may happen, for example, if a company provides funds to a university researcher, whereby the university would become the sponsor of the review. Where relevant, the sponsor should be named in a review protocol.

Item 5c: Role of sponsor and/or funder. Describe roles of funder(s), sponsor(s), and/or institution(s), if any, in developing the protocol

Example

"The Nova Scotia Health Research Foundation (NSHRF) is funding the Chronic LBP IPD Meta-analysis project. This funding will support the collection of the individual participant data by the original investigators, data management and analyses. The NSHRF is not involved in any other aspect of the project, such as the design of the project's protocol and analysis plan, the collection and analyses. The funder will have no input on the interpretation or publication of the study results."[66]

Explanation

When the sponsor or funder (sometimes the same entity) with competing interests has a substantial role in the planning, conduct, or dissemination of a systematic review, there is potential for bias if authors do not manage the interests of all parties appropriately. Although both industry and non-industry reviews are subject to potential bias(es), published reports of reviews with commercial sponsorship tend to describe lower quality methods and more favourable conclusions.[58-60 67] Examples exist of unfavourable reviews being suppressed by commercial sponsors.[68 69]

To provide full transparency into the potential relevance of competing interests, review protocols should explicitly describe the roles (if any) of the sponsor and funders in protocol development, review conduct, data analysis and interpretation, and dissemination of the final report. It is important to specify who will make the final decision about these elements of the systematic review, particularly if disagreements arise. Any restrictions on disseminating the final report of the review should also be documented.

Section 2: Introduction

Rationale

Item 6. Describe the rationale for the review in the context of what is already known

Example

[Review title: Trends in child and adolescent obesity prevalence according to socioeconomic position: protocol for a systematic review]

"It is well recognised that childhood obesity is a significant public health issue, with adverse physical and psychological effects that persist beyond childhood into the adult years. After decades of rapid increase, it appears that childhood obesity prevalence in developed countries is starting to plateau. Reviews of international evidence have shown that the prevalence of obesity in children and adolescents is stabilising in countries including Australia, Japan, France, the UK and US. However, evidence also suggests that such progress may not have been shared among children across all socioeconomic groups.

An international systematic review published in 2010 examined obesity prevalence trends and reported levelling off of the obesity epidemic in recent years. Heterogeneity in obesity trends were reported across socioeconomic strata, with levelling of obesity prevalence less apparent for more disadvantaged socioeconomic groups. However, the authors noted that trends by socioeconomic strata were only explored in a small number of their included studies. Individual studies reporting the impact of socioeconomic position (SEP) on obesity prevalence provided mixed results. Studies from Australia and England reported socioeconomic differences in obesity trends among children and adolescents, while evidence from France did not show a difference. With a specific focus on SEP and childhood obesity, this review will capture additional data, including papers published since 2010, to allow greater understanding of trends in the prevalence of obesity by SEP.

Further investigation is warranted, particularly because of the existing excess burden of obesity in children in a lower SEP. Given the health risks associated with excess weight, and the observed socioeconomic patterning in chronic diseases, if trends in obesity prevalence are not improving at the same rate across socioeconomic groups, this will likely lead to further inequalities across a range of health and wellbeing outcomes. Understanding the differences between subgroups of the population is critical to ensuring policy makers can make informed decisions as to where preventive efforts should be focused. This is particularly important in light of evidence that demonstrates differential effectiveness of a number of obesity prevention interventions according to SEP."[70]

Explanation

Readers need to understand the rationale behind the decision to perform the systematic review and what the results may add to what is already known. Authors should explain the impetus for the systematic review (such as to support clinical guideline development, to address uncertainty or variation in practice in approaches to a specific clinical problem, to support policy development, to provide a more precise estimate of effect, to update a previous review) and briefly summarize how the review builds on and could add to prior knowledge. In the case of a protocol to update an existing review, authors should cite the previous or original review and, in the methods section, point out any planned modifications from the original review in the protocol for the update,[71] perhaps with a section heading "updated methods." Where possible, the primary audience for

For peer review only

No commercial reuse: See rights and reprints http://www.bmj.com/permissions Subscribe: http://www.bmj.com/subscribe

BMJ 2015;349:g7647 doi: 10.1136/bmj.g7647 (Published 2 January 2015)

the review and the review perspective (that is, patient or clinician decision making, public health, health policy) should be clear.

Ideally, the rationale section should set the context for both the protocol as well as the systematic review. Background detail on the clinical condition should be sufficient to help the reader establish the overall significance of the proposed systematic review for developing new knowledge of interest and to help clarify key decisions or processes undertaken in the research protocol. These might include the specific focus of the population, intervention, comparator(s), and outcome (with emphasis on specific outcomes), settings, study designs, and time frames. As well, the means by which key perspectives represented in the review were obtained (that is, patient or other stakeholder engagement) should be described.

Objectives

Item 7. Provide an explicit statement of the question(s) the review will address with reference to participants, interventions, comparators, and outcomes (PICO)

Example 1

"The aim of this systematic review is to evaluate the effectiveness and harms of perioperative pregabalin in the management of postoperative pain for the diverse patients undergoing various surgical procedures. To this end, the proposed systematic review will answer the following questions:

1. When compared with standard multimodal analgesia, what are the comparative effectiveness and harms of the co-administration of pregabalin in the perioperative pain management of adult patients?

2. Is there a definitive opioid-sparing advantage of pregabalin (for example, lower risk of nausea, vomiting, somnolence, opioid use, and other opioid-related side effects) when used for perioperative pain management in adults?

3. For questions 1 and 2 above, what clinical and study methodological characteristics explain the heterogeneity in results?"[72]

Example 2

"The objectives of our study are to systematically review the literature for qualitative evidence that explores the factors that influence the decision of individuals aged 50 years or over at average risk for CRC to participate in CRC screening, and how those factors vary by sex, ethnicity and SES. Our secondary aim will be to generate a framework to better understand the perceived benefits and barriers that affect individual decision-making."[73]

Explanation

Among the most crucial pieces of information to include in a review protocol are the question(s) the reviewers plan to investigate, or simply, the review's objectives. Along with the review's rationale (Item 6), this information provides the reader with context and understanding for why the review is being carried out and what the reviewers hope to achieve. Several key components, namely the planned population, intervention, comparator, and outcome (that is, PICO elements) at minimum should form the basis for developing a specific, well designed review question. Additional elements such as setting, study design, and time frame (that is, length of follow-up) may also be included in the review question, but if not, should certainly appear in the review's eligibility criteria (Item 8). Guidance is available to help researchers develop a research question.[74 75]

Reviews may focus on one PICO element more than others given the planned scope of the review; authors should clearly state this emphasis in the protocol.

Section 3: Methods

Eligibility criteria

Item 8. Specify the study characteristics (such as PICO, study design, setting, time frame) and report characteristics (such as years considered, language, publication status) to be used as criteria for eligibility for the review

Example:

"**Eligibility criteria**

"Studies will be selected according to the criteria outlined below.

Study designs

We will include randomized controlled trials (RCTs), including cluster RCTs, controlled (non-randomized) clinical trials (CCTs) or cluster trials, interrupted time series (ITS) studies with at least three data points before and after the intervention, controlled before-after (CBA) studies, prospective and retrospective comparative cohort studies, and case-control or nested case-control studies. Cluster randomized, cluster non-randomized, or CBA studies will be included only if there are at least two intervention sites and two control sites. We will exclude cross-sectional studies, case series, and case reports.

Participants

We will include studies examining the general adult human population or healthy adult humans (18 years or older). We will also include studies on people who are overweight or obese, but will otherwise exclude studies of populations restricted to specific diseases, conditions, or metabolic disorders. We will include studies addressing both adults and children if data provided for adults are reported separately.

Interventions

Of interest are interventions addressing SSB consumption, taking a broad perspective. In addition to direct consumption studies, we would consider interventions that influence consumption, such as those addressing the level of access to SSBs (e.g. university/college policy) and educational interventions addressing consumption as relevant. Non-specific or multi-faceted behavioural, educational, or policy interventions may also be included subject to the level of evidence that exists for the aforementioned interventions/exposures. We will also consider other types of interventions on a case by case basis, subject to what exists in the literature.

In terms of defining an SSB, we view them as akin to a complex intervention because they are composed of several parts. For example, in addition to sugar, some beverages contain caffeine and the by-products of caramel colouring (2-methylimidazole, 4-methylimidazole), which may contribute independently to adverse health outcomes. The scope of the review, therefore, warrants an examination of SSB consumption as a whole, rather than the specific constituents as exposure variables. Otherwise, such evaluations would have necessarily required the inclusion of studies addressing those constituents and in foods and drinks other than SSBs.

We will use the Centers for Disease Control and Prevention (CDC) definition of SSB for drinks that should be included. According to the CDC, SSBs contain added caloric sweeteners, which would include natural sweeteners such as honey and concentrated fruit juice. We have developed a classification

For peer review only - http://bmjopen.bmj.com/site/about/guidelines.xhtml

No commercial reuse: See rights and reprints http://www.bmj.com/permissions Subscribe: http://www.bmj.com/subscribe

scheme based on the CDC definition for use during the review (see classification scheme for SSBs below). For beverages such as coffee, tea, and homemade lemonade, studies will be included in the review if they explicitly state that sugar was added. We will exclude artificially sweetened (e.g. with aspartame or sucralose) beverages, alcoholic beverages, and 100% fruit or vegetable juices as exposures/interventions.

We will classify SSBs described in studies according to the following broad categories:

- Sodas-caffeinated/non-caffeinated (soft drinks, soda, pop, soda pop)
- Other non-carbonated sweetened beverages (fruitades, fruit drinks, fruit punches, [iced] teas, coffees, non-dairy fruit smoothies)-caffeinated/non-caffeinated
- Fortified sweetened beverages (energy drinks, fortified waters, sports drinks)-caffeinated/non-caffeinated and containing vitamins, amino acids, herbal stimulants, or other ingredients
- Flavored/sweetened milk or milk alternative beverages (dairy, soy, almond, milkshakes, dairy based fruit smoothies)-caffeinated/non-caffeinated

Comparators

Given the broad perspective for interventions of interest, several comparisons will be relevant to include. Some may be more likely to come from observational designs and others from experimental studies.

Direct consumption studies:

1. SSB consumption compared with consumption of non-SSB drink (e.g. 100% fruit juice, artificially sweetened beverage, water)
2. Higher level of SSB consumption versus lower level of SSB consumption for the same drink type (e.g. carbonated cola beverages)
3. Comparisons among different categories of SSBs (e.g. soft drinks compared with fruit drinks; see classification scheme for SSBs) consumed in similar amounts

Interventions that influence consumption:

4. One level of access to SSB compared with another level of access (e.g. university/college policy on beverages in vending machines)
5. Educational intervention to specifically promote lower or no SSB consumption compared with no educational intervention/regular curriculum coverage/general health-focussed intervention
6. Non-specific or multi-faceted educational, behavioural, or policy dietary intervention (may include component of SSB consumption) compared with no intervention
7. Other comparisons involving interventions that address our research question (interventions assessed on a case by case basis, as encountered in the literature)

For comparator groups 2 and 3, we anticipate that volume will be the most feasible to analyse; however, we will extract all measures in which consumption is reported (e.g. volume, caloric intake from sugar) in studies to see what analysis is possible.

For feasibility, category 6 comparisons (non-specific, multi-faceted interventions) will be coded at title/abstract screening and not put through to full text screening. If sparse evidence exists in the other potential comparison types, we will revisit eligibility for comparison 6.

Outcomes

Endpoints important for decision making are of primary interest. If reported on, these will be analysed and graded. If a given clinical endpoint is not reported on, we will analyse and grade their relevant surrogate outcome(s).

- Endpoints important for decision making:
  - Adverse cardiovascular (including cerebrovascular) events
  - Cancer (excluding basal cell and squamous cell carcinoma)
  - Chronic kidney disease
  - Mortality
  - Overweight/obesity
  - Type 2 diabetes
  - Dental caries
  - Quality of life (generic, validated tools only, such as those in Additional file 2)
  - Gout
- Surrogate outcomes:
  - Pre-diabetes
  - Metabolic syndrome
  - Change in cardiovascular disease (CVD) risk
  - Progression of obesity
  - Dyslipidemia
  - Hypertension

As some outcomes may be reported as a composite measure, we will extract all composite and individual outcomes as reported in the studies.

Outcomes will be collected as reported, with the exception of quality of life, which will be collected only if assessed with generic (not disease specific), validated tools. Due to possible variation in disease definitions over time, we will extract definitions of outcomes as reported in individual studies. We will extract outcomes in all data forms (e.g. dichotomous, continuous) as reported in the included studies.

Timing

Studies will be selected for inclusion based on the length of follow-up of outcomes. The following will be used as a guide for all study designs:

- For all decision making endpoint outcomes, studies should have a follow-up time of at least 1 year.
- For all surrogate outcomes, studies should be at least 6 months duration for follow-up.
- For cancer, studies should be at least 1 year duration for follow-up. Some types of cancer may need longer than a 1 year follow-up, but this will be evaluated on a case by case basis.

Setting

There will be no restrictions by type of setting.

Language

We will include articles reported in the English and French languages. A list of possibly relevant titles in other languages will be provided as an appendix.”[76]

Explanation

The requirement and ability to pre-specify eligibility criteria (sometimes denoted inclusion or exclusion criteria) that reviewers will use to identify relevant studies for inclusion is a defining feature of a systematic review.[77] Making this information available to readers of protocols, as in completed

For peer review only - http://bmjopen.bmj.com/site/about/guidelines.xhtml
No commercial reuse: See rights and reprints http://www.bmj.com/permissions
Subscribe: http://www.bmj.com/subscribe

BMJ 2015;349:g7647 doi: 10.1136/bmj.g7647 (Published 2 January 2015)

reviews, is essential in appraising the validity, applicability, and comprehensiveness of a review.[74] Thus, authors should provide an unambiguous description of planned eligibility criteria for the impending review; such descriptions are a fundamental component upon which later stages of the review process are conducted. For instance, eligibility criteria often influence the terminology used to develop the search strategy and work to prevent the introduction of bias into the study selection process of a systematic review.

As in PRISMA, there are two general categories of eligibility criteria: study characteristics and report characteristics.[17] Authors should describe both. As in the example above, authors can anticipate that these details will require substantial space in the methods section of a review protocol while at the same time facilitating review transparency and future reproducibility.

Study eligibility criteria are the typical PICO elements that form the basis of clinical questions. These include populations, interventions, comparators, outcomes, time frames for follow-up, settings in which the interventions are delivered, and study designs of interest; they also can include other study specific elements, such as specifying a minimum length of follow-up or a minimum sample size for certain types of studies. Authors should state whether they will exclude studies because the studies do not include (or report) specific outcomes; doing so will help readers ascertain whether the eventual review may be biased as a consequence of selective reporting.[4]

Review eligibility criteria are likely to include geographical location, languages of publication, publication status (such as inclusion of unpublished material or abstracts), and years of publication. Inclusion or not of literature in multiple languages,[78 79] unpublished data, or older data can influence the effect estimates in meta-analyses.[80 81] If it is planned to filter out (via search filter, see Item 10) or exclude specific types of records (such as commentaries, letters, editorials, etc) during screening, this should be stated.

Information sources

Item 9. Describe all intended information sources (such as electronic databases, contact with study authors, trial registers or other grey literature sources) with planned dates of coverage

Example

"Literature search strategies will be developed using medical subject headings (MeSH) and text words related to influenza vaccination. We will search MEDLINE (OVID interface, 1948 onwards), EMBASE (OVID interface, 1980 onwards), and the Cochrane Central Register of Controlled Trials (Wiley interface, current issue). The electronic database search will be supplemented by searching for trial protocols through metaRegister (http://www.controlled-trials.com/mrct/). The literature search will be limited to the English language and human subjects.

To ensure literature saturation, we will scan the reference lists of included studies or relevant reviews identified through the search. We will also search the authors' personal files to make sure that all relevant material has been captured. Finally, we will circulate a bibliography of the included articles to the systematic review team, as well as to influenza experts identified by the team."[82]

Explanation

A systematic review search typically includes a variety of information sources including electronic bibliographic databases (such as Medline, Embase), reference lists, contact with authors of included studies, study registries, and grey literature. Most biomedical topics will include a Medline search, plus additional electronic databases. Searching additional electronic databases helps ensure more complete coverage of the topic by accounting for variability between the indexing in each database. In situations in which identifying all relevant studies through hand searching and database searching is difficult, if any other searching, such as reference lists, is planned to supplement searching, authors should report this.[83] Documentation of the planned information sources should include the name of each source, the date range that was searched (that is, start and end dates, and, for electronic database searches, the search platform or provider such, as Ovid or PubMed). This information will be important to the person developing and conducting the search if an update to the review is carried out. Authors should also report who developed and carried out the search.[83 84]

The Cochrane Collaboration,[85] AHRQ's Effective Health Care Program,[86] and the Institute of Medicine (Standard 3.1),[14] among others, offer guidance on developing a rigorous systematic review search strategy. If these sources are used, authors should report this information.

Search strategy

Item 10. Present draft of search strategy to be used for at least one electronic database, including planned limits, such that it could be repeated

Example

"Both qualitative and quantitative studies will be sought. No study design, date or language limits will be imposed on the search, although only studies in languages other than English that can be translated adequately using Google translate1 will be included, due to resource limits. Medline, EMBASE, PsycINFO, and the CENTRAL trials registry of the Cochrane Collaboration will be searched. The specific search strategies will be created by a Health Sciences Librarian with expertise in systematic review searching. The MEDLINE strategy will be developed with input from the project team, then peer reviewed by a second librarian, not otherwise associated with the project, using the PRESS standard.2 A draft MEDLINE search strategy is included in Appendix 1. After the MEDLINE strategy is finalized, it will be adapted to the syntax and subject headings of the other databases.

As well, the International Clinical Trials Registry Platform Search Portal and ClinicalTrials.gov will be searched for ongoing or recently completed trials, and PROSPERO will be searched for ongoing or recently completed systematic reviews. As relevant studies are identified, reviewers will check for additional relevant cited and citing articles.

"The search will be updated toward the end of the review, after being validated to ensure that the MEDLINE strategy retrieves a high proportion of eligible studies found through any means but indexed in MEDLINE.

…

Appendix 1

Draft MEDLINE search - Ovid interface

1. Infant, Extremely Premature/
2. Infant, Extremely Low Birth Weight/
3. Infant, Very Low Birth Weight/
4. (extreme* adj2 preterm).mp.

For peer review only - http://bmjopen.bmj.com/site/about/guidelines.xhtml

No commercial reuse: See rights and reprints http://www.bmj.com/permissions Subscribe: http://www.bmj.com/subscribe

5. (extreme* adj2 prematur*).mp.

6. extreme* low birth weight.mp.

7. (low gestational age neonate* or ELGAN*).mp.

8. very preterm.mp.

9. very premature.mp.

10. ELBW.mp.

11. ((limit* adj2 viability) or (margin* adj2 viability)).tw. or (22 week* or 23 week* or 24 week* or 25 week* or 26 week* or (26* adj5 week*) or (27* adj5 week*) or (28* adj5 week*) or (29* adj5 week*) or (30* adj5 week*) or (31* adj5 week*) or 32* week* or (32* adj2 fewer week*) or (32* adj2 less week*)).mp.

12. resuscit*.mp.

13. exp Obstetric Labor, Premature/

14. or/1-13

15. exp Parents/ or parent*.tw. or mother*.tw. or father*.tw.

16. Decision Making/

17. Counseling/

18. Advance Care Planning/ or Advance Directives/

19. (counsel* and decision*).mp.

20. or/16-19

21. (deliver* or predeliver* or prenatal* or antenatal* or perinatal*).mp.

22. 14 and 15 and 20 and 21"[87]

Explanation

The comprehensiveness and completeness of a literature search is extremely important in systematic reviews. High quality searches of information resources are essential components in the efforts toward accuracy and completeness of the evidence base.[88]

At a minimum, authors should provide the transcript of a draft search strategy for one major database (such as Medline) for each search question (if different searches were run for each question). In the documented strategy, it should be evident which indexing terms reviewers selected and what limits (such as language and date restrictions) were (or will be) applied to the search. If authors plan to use any search filters, information about their validity and performance metrics should be provided. Authors should also describe the planned search strategy approach for other databases, including planned modifications to indexing terms, free text terms, and limits, which may vary across databases.

If limits were used to restrict the search to particular study type (that is, trials, human, or clinical studies) or date range, authors should report what these were and how they were achieved. Simply stating, for example, that all publications in the form of letters will be excluded from the search can be problematic given that the publication of randomised trials as "letters to the editor," is a documented problem,[89] and authors may be intending to make an exception for such reports. Authors should report the logical construction of text used to create such limits within the draft search strategy (such as "NOT (letter.pt NOT randomized controlled trial.pt")).[90] Doing so can help readers assess the appropriateness of intended limits within a search strategy.

Most searches have constraints—for example, relating to limited time or financial resources, inaccessible or inadequately indexed reports and databases, unavailability of experts with particular language or database searching skills, or review questions for which pertinent evidence is not easy to find. Authors should be straightforward in describing their search constraints.[17]

Authors should also report the approach that was or will be taken in the development of a search strategy, including qualifications of the searcher (such as a health information specialist with systematic review experience), planned databases to be searched (see Item 9), limits to be imposed (to demonstrate alignment with review eligibility criteria), and whether the search was or will be peer reviewed and by whom.[91] Having a search strategy peer reviewed may help to increase its comprehensiveness or decrease yield where search terminology is unnecessarily broad.

The draft search strategy can be presented in the body of the text or as a table. If the protocol is being published in a journal, the journal may advise on this issue (that is, in their instructions to authors). If space is a concern, authors should ask the editor whether it can be included it as a web based appendix or whether an electronic link to where it can be found can be provided in the manuscript.

Providing details of the planned search strategy will allow readers of systematic review protocols to appraise and avoid potential duplication of efforts, as well as possibly enhance the development of their own searches. Including at least one main search strategy can also specifically facilitate updating.

Study records

Item 11a: Data management. Describe the mechanism(s) that will be used to manage records and data throughout the review

Example

"Literature search results will be uploaded to Distiller Systematic Review (DSR) Software, an Internet based software program that facilitates collaboration among reviewers during the study selection process. The team will develop and test screening questions and forms for level 1 and 2 assessments based on the inclusion and exclusion criteria. Citation abstracts and full text articles will be uploaded with screening questions to DSR. Prior to the formal screening process, a calibration exercise will be undertaken to pilot and refine the screening questions. Further, we will provide training to new members of the review team not familiar with the DSR software and the content area prior to the start of the review."[54]

Explanation

Systematic review data management software is becoming increasingly common. Examples of web based software are Distiller SR and Eppi-Reviewer. These web based software management programs are helpful in managing small or large scale datasets by allowing importation of citations and PDFs to be screened and included. They may reduce data entry errors during the data extraction process by allowing direct entry into pre-created data extraction forms and export of data directly into statistical analysis software. They may also facilitate the creation of a PRISMA flow diagram once the screening process is completed. Whether use of such software is planned to manage records in the review should be described in the protocol. Several other tools may be used during the review process to de-duplicate references (such as reference management software) and to extract or manage data (such as electronic software).[92] Reviewers using more traditional forms of data management should also describe their process.

For peer review only - http://bmjopen.bmj.com/site/about/guidelines.xhtml

No commercial reuse: See rights and reprints http://www.bmj.com/permissions Subscribe: http://www.bmj.com/subscribe

Whatever process is used, it should be described in sufficient detail so that interested readers can replicate the process.

Some studies are published more than once. Duplicate publications may be difficult to ascertain, and their inclusion may introduce bias.[93 94] We ask authors to describe any steps they are proposing to use to avoid double counting and to piece together data from multiple reports of the same study (such as juxtaposing author names, treatment comparisons, sample sizes, or outcomes). We also recommend that authors indicate whether all reports on a study were considered, as inconsistencies may reveal important limitations. For example, a review of multiple publications of drug trials showed that reported study characteristics may differ from report to report, including the description of the design, number of patients analyzed, chosen significance level, and outcomes.[95] See Item 12 (data items) for more information.

Item 11b: Selection process. State the process that will be used for selecting studies (such as two independent reviewers) through each phase of the review (screening, eligibility, and inclusion in meta-analysis)

Example

"The review authors will independently screen the titles and abstracts yielded by the search against the inclusion criteria. We will obtain full reports for all titles that appear to meet the inclusion criteria or where there is any uncertainty. Review author pairs will then screen the full text reports and decide whether these meet the inclusion criteria. We will seek additional information from study authors where necessary to resolve questions about eligibility. We will resolve disagreement through discussion. We will record the reasons for excluding trials. Neither of the review authors will be blind to the journal titles or to the study authors or institutions."[96]

Explanation

Reviewers will often identify a large number of studies from electronic database searches, and then use pre-defined eligibility criteria (Item 8) to determine which records are relevant and should be included in the review. There is currently no agreed process for how studies should be selected for inclusion in a systematic review. For example, it is unclear whether all records identified by the search should be initially screened for potential inclusion by two independent reviewers, or if only those noted as excluded by one reviewer should be. Protocol authors should therefore describe their specific approach for identifying potentially eligible records (that is, by title and abstract screening) and for selecting studies for final inclusion (that is, by full text screening). Typical methodology for study selection is aimed at enhancing objectivity and preventing mistakes. Often, screening is carried out in duplicate by independent reviewers at each stage of the review to reduce the possibility of excluding relevant reports.[97] The benefit may be greatest for topics where selection or rejection of an article requires difficult judgments.[98]

Authors should report whether one or several persons will be involved in each stage of screening and name those who will be involved, if known. If independent screening is planned, authors should describe the process for dealing with discrepancies (such as third party arbitration or contacting authors of original studies) and whether inter-rater agreement will be calculated.

Item 11c: Data collection process. Describe planned method of extracting data from reports (such as piloting forms, done independently, in duplicate), any processes for obtaining and confirming data from investigators

Example

"Using standardized forms … and a detailed instruction manual that will be used to inform specific tailoring of an online data abstraction program (DistillerSR), ten teams of reviewers will extract data independently and in duplicate from each eligible study. To ensure consistency across reviewers, we will conduct calibration exercises before starting the review. Data abstracted will include demographic information, methodology, intervention details, and all reported patient-important outcomes. Reviewers will resolve disagreements by discussion, and one of two arbitrators (JWB or GHG) will adjudicate unresolved disagreements. We will contact study authors to resolve any uncertainties."[99]

Explanation

Reviewers should plan and document the approach they plan to use to extract data from included studies in the review along with which data items (Item 12) and types of data. Data extraction forms should be developed a priori and included in the published or otherwise available review protocol as an appendix or as online supplementary materials.

As with screening, data extraction is often carried out in duplicate by independent reviewers or by one reviewer with verification by another in order to reduce bias and reduce errors in data extraction. The planned approach for resolving discrepancies should be stated. Although single data extraction has not been shown to substantially affect treatment effect estimates, reviewers should explicitly indicate whether single extraction will be employed to allow reviewers and readers to be more mindful of the possibility for errors in the completed review.[100]

Data extraction can be complicated, especially with more complex topics, and level of reviewer experience has not been shown to affect extraction error rates.[101 102] As such, additional strategies planned to reduce errors, such as training of reviewers and piloting of extraction forms should be described. In addition, if reviewers plan to make use of data extraction techniques to obtain outcome data not reported in a usable format, such as translating graphically presented data into a usable (that is, numeric) format,[103] they should plan for this during the protocol stage and report details of proposed software and its sensitivity and specificity.

If an individual patient data (IPD) meta-analysis is planned, authors should also tell readers when and how they sought individual patient data from the original researchers.[104] Data extraction for IPD reviews will often involve collection and scrutiny of detailed raw databases; authors should describe their planned approach clearly. The description might include how they attempted to contact researchers, what they asked for (that is, using a reply form with pre-specified data items), and their plan if they are unable to obtain all requested information. For IPD meta-analyses or otherwise, reviewers should also state whether they intend to confirm the accuracy of the extracted information to be included in their review with original researchers, for example, by sending them a copy of the draft review when available.[105]

Data in primary studies may not always be presented in a format that is useful to systematic reviewers. Contacting authors for

For peer review only - http://bmjopen.bmj.com/site/about/guidelines.xhtml

missing information about treatments, for example, has been shown to improve the completeness of treatment descriptions by at least 27%.[106] Ideally, authors of primary studies should be urged to report all aspects of their studies more clearly.[107] However, in the absence of complete descriptions of treatments, outcomes, effect estimates, or other important information, reviewers may consider asking authors for this information. Whether reviewers plan to contact authors of included studies and how this will be done (such as a maximum of three email attempts) to obtain missing information should be documented in the protocol.

Knowledge of duplicate, overlapping, or companion studies (that is, multiple reports of a single study) may come to light only during the data extraction process.[94] The inclusion of data from multiple reports as separate studies may lead to biased treatment effects[93] and should be anticipated by reviewers. Methods for identifying and dealing with multiple reports of a single study have been described.[108 109] Authors should present the algorithm they will follow to select data from overlapping reports and the planned approach for solving logical inconsistencies across reports.

Data items

Item 12. List and define all variables for which data will be sought (such as PICO items, funding sources) and any pre-planned data assumptions and simplifications

Example 1

"We will extract the generic and the trade name of the experimental intervention, the type of control used, dosage, frequency and duration of treatment, patient characteristics (average age, gender, mean duration of symptoms, type of joints affected), type of pain or function related outcome extracted, trial design, trial size, duration of follow-up, type and source of financial support and publication status from trial reports. For non-pharmacological interventions, we will extract type, modes of application and intensity, if appropriate. When necessary, means and measures of dispersion will be approximated from figures in the reports. For cross-over trials, we will extract data from the first period only because of possible carry-over effects. Whenever possible, we will use results from an intention to treat analysis. If effect sizes cannot be calculated, we will contact the authors for additional data."[110]

Example 2 (data simplifications)

"It is possible that individual studies may consist of multiple treatment groups, such as different types of depression interventions or different doses of medication. In order to avoid the possibility of introducing bias caused by multiple statistical comparisons with one control group, we will combine the groups from multiple arm studies into a single group."[111]

Explanation

Readers need to know what information review authors plan to obtain from the included studies. Data items and pre-specified time points are essential to document in a review protocol because this information allows readers to refer back to the protocol when the review is complete to determine whether changes occurred. Extraction forms should include definitions of variables, with particular details about the planned outcomes, and their measurement duration and frequency (Item 13).

The selective reporting of information in reviews is a documented concern.[8 36] Providing readers with the opportunity to identify and make their own judgments about selective reporting is crucial.[112] If the review is limited to reporting only those variables that were obtained, rather than those that were deemed important a priori but could not be obtained, bias might be introduced and the reader might be misled. In protocol amendments and completed reviews, authors should clearly outline whether any data items were added after the protocol was developed or after the review began and give the reasons why. Such variables might include aspects of treatments or outcomes identified as important because they recur during the review process (such as important outcome measures that the reviewers initially overlooked). A more complete discussion of selective outcome reporting in systematic reviews and related bias is found in Item 13.

Authors should describe assumptions they intend to make if they encounter missing or unclear information and explain how they plan to deal with such data or lack thereof, in addition to contacting authors (Item 11c). For example, in studies of women aged 50 or older it may be reasonable to assume that none was pregnant even if this is not reported. Ideally, authors should anticipate as many uncertainties as possible before they arise and have a documented, agreed approach for dealing with such data. Likewise, review authors might make assumptions about the route of administration of drugs assessed. However, a more prudent approach is required when dealing with qualitative information. For example, the upper age limit for "children" can vary from 15 years to 21 years, or the level of severity of an outcome (such as an adverse effect) might be poorly described in primary research and mean very different things to different researchers at different times and for different patients.

If simplifications such as combining treatment arms (for multiple treatment trials) or using first period data for cross over trials are planned, these should be described.

Outcomes and prioritisation

Item 13. List and define all outcomes for which data will be sought, including prioritisation of main and additional outcomes, with rationale

Example

"*Primary outcomes*

"The primary outcome will be the number of patients who responded to treatment, defined as a reduction of at least 50% on the Hamilton Depression Rating Scale (HAM-D), the Montgomery-Asberg Depression Rating Scale (MADRS) or any other depression scale, or 'much or very much improved' (score 1 or 2) on the Clinical Global Impression (CGI) Improvement Scale. All response rates will be calculated from the total number of randomised patients. Where more than one criterion is provided, we will use the HAM-D for judging the response and then follow the sequence described above. Despite the problems surrounding scale-derived response cutoffs, dichotomous outcomes can be understood more intuitively by clinicians than the mean values of rating scales and are therefore preferred.

When studies report response rates at various time points of the trial, we have decided a priori to subdivide the treatment indices as follows.

1. Early response, between one and four weeks, the time point closest to two weeks will be given preference.

2. Acute phase treatment response, between six and 12 weeks, the time point given in the original study as the study endpoint will be given preference.

For peer review only - http://bmjopen.bmj.com/site/about/guidelines.xhtml

BMJ 2015;349:g7647 doi: 10.1136/bmj.g7647 (Published 2 January 2015)

3. Follow-up response, between four and six months, the time point closest to 24 weeks will be given preference.

The acute phase treatment response, that is between six and 12 weeks, was our primary outcome of interest.

Secondary outcomes

1. The number of participants in remission, as defined by either: (a) at 7 or less on the 17-item HAM-D and at 8 or less for all the other longer versions of HAM-D; (b) at 10 or less on the MADRS; (c) 'not ill or borderline mentally ill' (score 1 or 2) on the CGI-Severity; or (d) other criteria as defined by the trial authors. All remission rates will be calculated out of the total number of randomised patients. Where two or more scales are provided, we prefer the first criteria for judging remission.

'Remission' is a state of relative absence of symptoms. This outcome adds to the primary outcome 'response' to treatment. The disadvantage of 'remission' is that its frequency depends on the initial severity of the participants. If they were only relatively mildly ill, many will be classified as in remission while only few will be in the case of high average severity at baseline. Therefore, studies and meta-analyses usually apply response and not remission as the primary outcome.

2. Change scores from baseline or endpoint score at the time point in question (early response, acute phase response, or follow-up response as defined above) on the HAM-D or MADRS, or any other validated depression scale. The results of mean values of depression rating scales can be more sensitive than dichotomous response data. Therefore, they should also be presented even though their interpretation is less intuitive than with dichotomous response data. Change data will be preferred to endpoint data but both will have to be presented separately because we will use the standardised mean difference as an effect size measure for which pooling of endpoint and change data is not appropriate. We prefer change scores to endpoint scores because they, to a certain extent, take into account small baseline imbalances.

3. Social adjustment, social functioning including the Global Assessment of Function scores.

4. Health-related quality of life as measured by validated disease specific and generic scales such as the Short Form (SF)-36 or the Health of the Nation Outcome Scales (HoNOS).

5. Various reasons for dropping out of the studies:

a) due to any reason, as a measure of the overall acceptability of treatment;

b) due to inefficacy of treatment, as a global efficacy measure;

c) due to adverse events, as a global measure of tolerability.

6. Death:

a) natural causes;

b) suicide;

c) suicide attempts.

7. Side-effects:

a) number of participants experiencing at least one side-effect, b) agitation or anxiety, c) blurred vision, d) constipation, e) urination problems, f) delirium, g) diarrhoea, h) dry mouth, i) fits, j) insomnia, k) hypotension, l) nausea, m) sedation or somnolence, n) vomiting, o) vertigo.

We anticipate including the following main outcomes in a summary of findings table using GRADEpro: response to treatment, acceptability of treatment (dropout due to any reason), quality of life, death due to suicide and overall tolerability (dropout due to adverse events)."[113]

Explanation

Systematic reviews must include a description of all outcomes (endpoints) of interest,[74] and by extension the same applies to protocols. Systematic reviews that aim to inform decision making should summarize both benefits and harms of interventions,[114] and specifying what those are during the planning phases of a review is, at minimum, a reminder or a commitment to do so. Review protocols should distinguish between which outcomes are considered the main outcome(s), also known as primary outcome(s), of a review and those that are additional (secondary) outcomes; these may differ from the prioritisation assigned to outcomes in primary studies.

Listing all outcomes for which data will be sought in a review and providing sufficient details and definitions are essential in a review protocol. Some outcomes may warrant additional details in their definitions such as distinctions between surrogate versus clinical, composite versus non-composite, and objective measurement versus subjective assessment. If, for example, a surrogate outcome is specified in lieu of a clinical outcome, a rationale as to why this was done and how the surrogate outcome is an indicator (associated) of a clinically important outcome should be stated. Consider, for example, a systematic review that focuses primarily on whether continuous positive airway pressure treatment reduces symptoms of somnolence and fatigue in patients with obstructive sleep apnoea (an abnormality of breathing patterns during sleep). The outcomes of interest should include instruments measuring symptoms (such as the Epworth Sleepiness Scale)[115] but not necessarily neurophysiological signals such as the frequency of apnoeas (no breathing) or hypopnoeas (reduced breathing), muscle tone, and heart rate variability, which are commonly reported but do not correlate well with symptoms.[116] Authors should do sufficient investigation during the planning stage to ensure that selected outcomes are relevant. Given increasing efforts to involve patients in the selection and assessment of outcomes,[117] reviewers should indicate whether planned outcomes are patient centred, and further, whether they are patient reported, and how such outcomes will be treated.[118]

The reporting of composite outcomes within a completed systematic review has been found to be variable across the abstract, methods, and results sections of the report.[119] Because the various components of a composite outcome have the potential to be combined in different ways, yielding differences in the direction, strength, and significance of an outcome, it is essential in a review protocol to state and define each component of a composite outcome explicitly, and, further, state how components within a composite outcome will be analysed, whether independently, all together, or in specific combinations (Item 15b).

Meta-analyses within systematic reviews are often limited by information available in included study reports. As such, discrete descriptions of the endpoints are not always possible at the protocol stage. The minimum and often only information one can practically specify is a broad description of the "outcome concept"—for example, what is the effect of an intervention on "survival or mortality." Such a description is too generic, and authors will need to refine it when they conduct their systematic review. Examples of more refined descriptions are "mortality at 12 months" or "mortality at 5 years" (for example, as odds ratios from cross tabulated counts of deaths at these follow-up durations) and "survival" (typically hazard ratios from time-to-event analyses). Reviewers should state their plans to refine outcome definitions based on definitions used in included studies.

For peer review only

For peer review only - http://bmjopen.bmj.com/site/about/guidelines.xhtml

No commercial reuse: See rights and reprints http://www.bmj.com/permissions

Subscribe: http://www.bmj.com/subscribe

Careful consideration of outcomes during the planning stages of a review can also improve efficiency in the review process. For example, if authors make a decision to add an outcome(s) at some point during data extraction, they will need to revisit all included papers to extract the additional information; this is a waste of reviewers' time. Minimizing such back and forth economizes time and resources and reduces the likelihood of mistakes.

The main outcome(s) of a review should be distinguished from additional outcomes and specific definitions of each should be provided. The scientific question or the decisional problem that motivates the systematic review typically dictates the main outcome(s) of interest. Thus for systematic reviews that aim to inform healthcare decisions or policy, the main outcomes are likely to be patient relevant outcomes (such as risk of stroke) or validated surrogate outcomes (for example, change in cholesterol levels is a valid surrogate for the risk of cardiovascular events for statin based interventions). In contrast, systematic reviews that aim to summarize the state of the science in the pathophysiology of a disease might appropriately choose biochemical or other measurements as main outcomes. All other outcomes are considered additional and are reviewed to provide complementary information and for completeness.

Listing and defining outcomes in a review protocol, as well as the prioritization of each as a main or additional outcome, will facilitate the ability of future readers of completed reviews to investigate selective reporting. Selective reporting of outcomes—that is, the addition, removal, or change in the priority of review outcomes between the protocol, methods section, and results of a review—is well recognized.[10 120] A 2010 study comparing Cochrane protocols with the completed reviews found that 22% of Cochrane reviews had a discrepancy in at least one outcome measure compared with their protocols, at least 75% of which were attributable to changes in the primary outcome, some after knowledge of review findings.[10] This is described as outcome reporting bias and occurs when the reporting of an outcome is associated with its significance. Whether in a completed review, outcomes are prioritized as main or additional should not be dependent on their prioritization or statistical significance in included studies.

Readers will note that the contents of this item are overlapping with Item 8 (eligibility criteria). Given the importance of outcomes in the review process, issues in the selection of relevant outcomes, and their potential to be manipulated during the review process, we felt that an item specifically dedicated to the reporting of outcomes would greatly facilitate complete and transparent reporting around this item. Readers should also note that complete definition and description of planned review outcomes, as proposed above, will occupy substantial space in a review protocol.

Risk of bias individual studies

Item 14. Describe anticipated methods for assessing risk of bias of individual studies, including whether this will be done at the outcome or study level, or both; state how this information will be used in data synthesis

Example 1

"To facilitate the assessment of possible risk of bias for each study, we will collect information using the Cochrane Collaboration tool for assessing the risk of bias (Table 8.5.a in the Cochrane Handbook for Systematic Reviews of Interventions), which covers: sequence generation, allocation concealment, blinding, incomplete outcome data (e.g. dropouts and withdrawals) and selective outcome reporting. For each domain in the tool, we will describe the procedures undertaken for each study, including verbatim quotes. A judgement as to the possible risk of bias on each of the six domains will be made from the extracted information, rated as 'high risk' or 'low risk'. If there is insufficient detail reported in the study we will judge the risk of bias as 'unclear' and the original study investigators will be contacted for more information. These judgements will be made independently by two review authors based on the criteria for judging the risk of bias (Table 8.5.c in the Cochrane Handbook Higgins 2011). Disagreements will be resolved first by discussion and then by consulting a third author for arbitration. We will compute graphic representations of potential bias within and across studies using RevMan 5.1 (Review Manager 5.1). We will consider each item in the risk of bias assessment independently without an attempt to collate and assign an overall score."[121]

Example 2

"Included non-randomised studies may or may not have a comparison group. To assess the risk of bias within included … studies, the methodological quality of potential studies will be assessed by using the Newcastle-Ottawa scale (NOS) for assessing the quality of non-randomised studies in meta-analyses. The NOS for case-control and cohort studies will be adapted (Table 1) to meet the specific needs of this systematic review. The cohort scale will be modified for use in case series. Using the NOS, studies will be awarded a maximum of nine points on items related to the selection of the study groups, the comparability of the groups, and the ascertainment of outcome of interest. Using this modified score, case series will be eligible for a maximum of six points. This will be undertaken by two separate reviewers. Where there is disagreement, a third reviewer will be used as an arbitrator."[122]

Explanation

An assessment of the risk of bias (or "quality") of studies included in a review is an important component of any well planned or conducted systematic review. Such an assessment contributes to the evaluation of the overall strength of evidence of the review (Item 17). Established methods for assessing risk of bias in reviews have been documented.[123 124] Descriptions of the planned approach to assessing risk of bias should include the constructs being assessed and a definition for each, reviewer judgment options (high, low, unclear), the number of assessors, experience of assessors (training, piloting, previous risk of bias assessment experience), as well as method(s) of assessment (independent or in duplicate).[125] Whether reviewers are going to be blinded to studies should also be reported,[126 127] as well as whether agreement between reviewers will be evaluated and, if so, how.

Details of planned methods to summarise risk of bias assessments across studies or outcomes should be provided. Although authors may spend a large proportion of time assessing risk of bias in included studies, they are often silent on how the results might influence their review findings.[128 129] Thus, we encourage reviewers to think about this at the development stage and document their plans in the protocol. Authors should also describe how risk of bias assessments will be incorporated into data synthesis (that is, subgroup or sensitivity analyses) and their potential influence on findings of the review (Item 15c)[129] in the protocol.

The likelihood that the treatment effect reported in a systematic review represents the true effect depends on the validity of the

For peer review only - http://bmjopen.bmj.com/site/about/guidelines.xhtml

included studies, namely, the internal validity. Certain methodological characteristics of primary studies may be associated with their resulting effect sizes.[129-131] For example, trials describing inadequate methods of allocation concealment or with unclear concealment exaggerate treatment effects on average compared with trials reporting adequately concealed allocation[132] Therefore, authors should not only describe risk of bias methods and constructs to be assessed for each included study, but also describe how results of the assessment contribute to the overall findings of the review.[128] Additionally, authors should provide a rationale if they do not intend to assess risk of bias.

Many methods exist to assess the overall risk of bias in included studies, including scales, checklists, and individual components.[133 134] As summarized in the PRISMA elaboration document,[17] scales that numerically summarize multiple components into a single number are misleading and unhelpful.[135] Rather, authors should specify the methodological components that they plan to assess and how they plan to assess said components. Common markers of validity for randomised trials, in the Cochrane Risk of Bias tool,[123] include appropriate generation of random allocation sequence[136]; concealment of the allocation sequence[132]; blinding of participants, healthcare providers, data collectors, and outcome adjudicators[137 138]; and proportion of patients lost to follow-up.[139] Reviewers may also anticipate assessing other items that do not necessarily indicate bias, such as the impact of early stopping of trials for benefit,[140 141] industry sponsorship,[55 142] single trial centres,[143] and improper analyses or fabrication of primary study data.[144 145] If authors plan such assessments they should explain this information in the protocol.

Authors should give careful consideration to assessments for reviews that expect to include non-parallel group randomised controlled trials and studies of non-randomised design, for which methodological standards are currently under development.[146] The ultimate decision regarding which methodological features should be evaluated requires consideration of the strength of the empirical data, theoretical rationale, and the unique circumstances of the included studies within the context of the review question.

Data synthesis

Item 15a. Describe criteria under which study data will be quantitatively synthesised

Example 1

"If studies are sufficiently homogeneous in terms of design and comparator, we will conduct meta-analyses using a random-effects model."[121]

Explanation

Diversity in study populations, interventions, outcomes, or trial conduct may mean that including some studies in a meta-analysis, or even conducting meta-analyses at all, will be impossible. Authors should describe, with reference to the PICO criteria, the conditions that should be present before they will proceed with statistical synthesis (Item 15b). Thus authors might consider whether to include trials with differing formulations or doses of the experimental treatment, studies using differing versions of a technology (such as a device), studies with different age profiles in the sample population, or studies with different follow-up times.

Item 15b. If data are appropriate for synthesis, describe planned summary measures, methods of handling data, and methods of combining data from studies, including any planned exploration of consistency (such as I^2, Kendall's τ)

Example

"**Measures of treatment effect**

• For dichotomous outcomes

Dichotomous data (occurrence of angiographic restenosis, mortality; recurrence of myocardial infarction, heart failure, angina; adverse events and the major adverse cardiac effects) will be determined by using risk ratio (RR) with 95% confidence interval (CI). It has been shown that RR is more intuitive than the odds ratio (OR) and that OR tend to be interpreted as RR by clinicians, which leads to an overestimate of the effect.

• For continuous outcomes

Continuous outcomes will be analysed using weighted mean differences (with 95% CI) or standardized mean differences (95% CI) if different measurement scales are used. Skewed data and non-quantitative data will be presented descriptively.

Unit of analysis issues

The primary analysis will be per individual randomised; however, all included trials will be assessed in order to determine the unit of randomization and whether or not this unit of randomization is consistent with the unit of analysis. Special issues in the analysis of studies with non-standard design, like cluster randomised trials, cross-over trials, and studies with multiple treatment groups, will be addressed. For cluster randomised trials we will extract an interclass correlation co-efficient to modify the results according to the methods described in the *Cochrane Handbook for Systematic Reviews of Interventions*. For cross-over trials, a major concern is carry-over effect. We will only use the data from the first phase, guided by the Cochrane Heart Group. When a study has more than two treatment groups, we will present the additional treatment arms. Where the additional treatment arms are not relevant, they will not be taken into account. We will also acknowledge heterogeneity in the randomization unit and perform a sensitivity analysis.

Dealing with missing data

When there are missing data, we will attempt to contact the original authors of the study to obtain the relevant missing data. Important numerical data will be carefully evaluated. If missing data cannot be obtained, an imputation method will be used. We will use sensitivity analysis to assess the impact on the overall treatment effects of inclusion of trials which do not report an intention to treat analysis, have high rates of participant attrition, or with other missing data.

Assessment of heterogeneity

We will test the clinical heterogeneity by considering the variability in participant factors among trials (for example age) and trial factors (randomization concealment, blinding of outcome assessment, losses to follow-up, treatment type, co-interventions). Statistical heterogeneity will be tested using the Chi^2 test (significance level: 0.1) and I^2 statistic (0% to 40%: might not be important; 30% to 60%: may represent moderate heterogeneity; 50% to 90%: may represent substantial heterogeneity; 75% to 100%: considerable heterogeneity). If high levels of heterogeneity among the trials exist (I^2 >=50% or P <0.1) the study design and characteristics in the included

For peer review only - http://bmjopen.bmj.com/site/about/guidelines.xhtml

No commercial reuse: See rights and reprints http://www.bmj.com/permissions Subscribe: http://www.bmj.com/subscribe

studies will be analysed. We will try to explain the source of heterogeneity by subgroup analysis or sensitivity analysis.

Data synthesis

Each outcome will be combined and calculated using the statistical software RevMan 5.1, according to the statistical guidelines referenced in the current version of the *Cochrane Handbook for Systematic Reviews of Interventions*. The Mantel-Haenszel method will be used for the fixed effect model if tests of heterogeneity are not significant. If statistical heterogeneity is observed (I^2 >=50% or P <0.1), the random effects model will be chosen. If heterogeneity is substantial, we will not perform a meta-analysis; a narrative, qualitative summary will be done."[147]

Explanation

When authors intend to perform meta-analyses, they should specify the effect measure (such as relative risk or mean difference) (Item 13) and the statistical method (such as inverse variance, DerSimonian-Laird, Mantel-Haenszel, Bayesian) to be used and whether they plan to apply a fixed or random effects approach.[148] Although experts debate this topic, fixed effects meta-analyses have been shown to overestimate confidence in treatment effects; thus, reviewers may wish to use this approach conservatively.[149 150] If estimates of heterogeneity are to be used to decide between fixed and random effects approaches, authors should state the threshold of heterogeneity required.[151] If possible, authors should explain the reasons for these choices.

Reviewers should anticipate that data from included studies may not be in a suitable format for analysis or presentation in the review. For that reason, authors may need to take various steps to process the data, even if they do not plan meta-analyses. Authors should describe their plans for data processing, focusing on anticipated problems specific to their review. In trials with more than two intervention groups (for example, receiving similar but non-identical interventions), combining or splitting results across groups may be necessary.[152] If individual patient data (IPD) meta-analyses are planned, reviewers should consult the (forthcoming) PRISMA extension for IPD meta-analyses.[153]

For analyses of dichotomous data (that is, event data), authors should consider how best to handle rare events or when events are absent from some studies. Outcomes reported as measurement scales (such as for depression) may use different scales in different studies; results may need to be adjusted so that all scales are aligned (for example, so that low values represent good health on all scales).

Reviewers should also anticipate that some desired data will not be reported in included studies at all. In particular, standard deviations and standard errors may have to be reconstructed from other statistics such as P values and *t* statistics[154 155]; occasionally they may be imputed from the standard deviations observed in other studies.[156 157] In analyses of time-to-event data, reviewers should anticipate spending more time and caution during data extraction (for example, from Kaplan-Meier survival curves) and report how conversion to a consistent format is planned.[158]

Statistical combination of data from two or more separate studies in a meta-analysis may not always be necessary, feasible, or desirable. Regardless of the decision to combine individual study results, authors should report how they plan to evaluate between-study variability (heterogeneity or inconsistency), such as by using I^2 or Cochran's Q test. The consistency of results across studies may influence the decision whether to combine individual study data in a meta-analysis. If reviewers plan to use statistical estimates of consistency (such as I^2 or Kendall's τ) to determine whether to perform a meta-analysis, they should state this explicitly (Item 15a) and specify the required number.

Finally, the name (and version) of any software planned for completing meta-analyses should be reported.

Item 15c. Describe any proposed additional analyses (e.g., sensitivity or subgroup analyses, meta-regression)

Example

"**Subgroup analysis and investigation of heterogeneity**

Subgroup analyses will be used to explore possible sources of heterogeneity, based on the following.

- Patient characteristic (age, sex).
- Types of treatment (western medicine alone, western medicine plus Tong-xin-luo).
- Follow-up period (three, six, and 12 months).
- Type of stent (drug-eluting and non-drug eluting stent).

Sensitivity analysis

Sensitivity analysis will be performed in order to explore the source of heterogeneity as follows.

- Quality components, including full-text publications versus abstracts, preliminary results versus mature results, published versus unpublished data.
- Risk of bias (by omitting studies that are judged to be at high risk of bias)."[147]

Explanation

Investigating possible causes of between-study variability or exploring the robustness of meta-analyses by using subgroup analysis or meta-regression may be desirable. If authors plan such analyses, they should state this and specify the covariates anticipated for the analyses (such as disease type or severity, or treatment dose). For subgroup analyses, authors should describe how they will partition the covariate into subgroups (for example, what will constitute mild or severe disease, low or high treatment dose). Whether they plan a fixed or random effects approach and how they will evaluate residual heterogeneity should also be stated.

If any sensitivity analyses are intended—such as including or excluding small studies, studies with high risk of bias,[159] industry funded studies, or outlier studies—authors should describe their plan for doing so.

Item 15d. If quantitative synthesis is not appropriate, describe the type of summary planned

Example

"A systematic narrative synthesis will be provided with information presented in the text and tables to summarise and explain the characteristics and findings of the included studies. The narrative synthesis will explore the relationship and findings both within and between the included studies, in line with the guidance from the Centre for Reviews and Dissemination."[160]

Explanation

In nearly all cases, reviews will include a qualitative (narrative) synthesis or summary even if meta-analyses or other quantitative analyses have been done. If, in addressing items 15a, 15b, and 15c, authors have concluded that some or all of the expected data will not be suitable for combining quantitatively, they

For peer review only
For peer review only - http://bmjopen.bmj.com/site/about/guidelines.xhtml

should explicitly say so in the protocol and provide the rationale for such decisions. Then for item 15d they should describe the way they propose to present results in narrative form.

Established methods for narrative syntheses are available.[161 162] Authors should, to the extent possible at the protocol stage, highlight the order in which they will present information and what they will give in text or (only) in tables. They should describe what priority they will give to information about participant populations (such as overall patient groups before subgroups, subgroups defined by sociodemographics before those defined by coexisting conditions) and about interventions and comparisons of interventions (such as head to head trials before trials with placebo or usual care controls, ultimate health outcomes before intermediate outcomes, patient related outcomes before utilization outcomes, and so forth). For example, authors may say that they will present results in order by key question and, within key questions, in order of main then additional outcomes. In other cases, they might specify that results will be reported first by key questions but then by important comparisons and outcomes within comparisons.

In addition, authors should say whether they plan to report only on studies for which risk of bias was either low or moderate and omit studies with high risk of bias, or whether they expect to retain studies of any level of risk of bias in their analyses. They should note that levels of risk of bias for a given study may differ depending on the outcome of interest, so that some studies may be retained for certain key questions or outcomes but not for others. In some cases, authors might note that they will report on studies at high risk of bias only when they provide the available information or a critical outcome or population of interest.

Authors should describe how they plan to present information by type of study design (for example, report results only for randomised controlled trials, and then supplement the results with information drawn from non-randomised trials or non-experimental studies). In some cases authors may want to stratify how they present information based on key aspects of how studies were conducted (such as whether investigators, patients, and outcome assessors were all masked to intervention). If authors will focus on specific types of outcome measures, such as demonstrably reliable and valid instruments to measure depression or pain, they should report this information.

Regardless of how many quantitative analyses authors expect to present, they should indicate the extent to which they plan to use tables to summarize (*a*) the characteristics of studies (perhaps only those of low or moderate risk of bias) and (*b*) the principal comparisons or outcomes of concern.

In some cases, review authors may plan to do types of analyses other than meta-analyses. These may include cost of illness, cost of treatment, or cost effectiveness analyses, decision modelling analyses, or various types of subgroup analyses (independent of any required by a key question). In all these cases, authors should be as specific as possible about what they will attempt to do.

Meta-bias(es)

Item 16. Specify any planned assessment of meta-bias(es) (such as publication bias across studies, selective reporting within studies)

Example

"In order to determine whether reporting bias is present, we will determine whether the protocol of the RCT was published before recruitment of patients of the study was started. For studies published after July 1st 2005, we will screen the Clinical Trial Register at the International Clinical Trials Registry Platform of the World Health Organisation (http://apps.who.int/trialssearch). We will evaluate whether selective reporting of outcomes is present (outcome reporting bias). We will compare the fixed effect estimate against the random effects model to assess the possible presence of small sample bias in the published literature (i.e. in which the intervention effect is more beneficial in smaller studies). In the presence of small sample bias, the random effects estimate of the intervention is more beneficial than the fixed effect estimate. The potential for reporting bias will be further explored by funnel plots if ≥10 studies are available."[163]

Explanation

Authors should pre-specify any methods used to explore the possibility that the data identified are biased due to non-study related processes.[164] Such bias may result from non-publication of studies (publication or dissemination bias) and the reporting of a subset of measured outcomes and analyses within studies (outcome reporting bias) (see box 2).

Detecting or correcting for publication bias in a systematic review is difficult. The results of available studies may provide clues that some studies may be missing (such as when smaller studies have systematically different effect estimates than larger studies ("small study effects")).[165] Recommendations regarding appropriate graphical methods (such as funnel plots) and statistical methods (such as Egger's test) to assess small study effects have been proposed.[166] However, publication bias is only one of several possible explanations for small study effects, and the interpretation of such tests can be problematic.[166-168] Authors should report their planned testing strategy to assess publication bias in detail. The risk of publication bias was formally assessed in only 21% of 100 intervention reviews published in 2006, and only 32% considered this type of bias.[169] A review of antidepressant trials found that effect estimates of meta-analyses of only the published trials were 32% larger on average than effect estimates of meta-analyses including published and unpublished trials.[170] The corresponding magnitude of publication bias in antipsychotic trials was smaller (8%).[171]

Several methods to detect selective outcome reporting exist. If a study protocol is available, reviewers can compare outcomes reported in the protocol and the published report.[7 172] Comparing the outcomes reported in the methods and results sections of the published report is an option when a protocol is unavailable.[173] For some trials, reviewers might assume that it is likely that an outcome was measured even if it was not reported, based on knowledge of the clinical area (such as when systolic, but not diastolic, blood pressure is reported).[112] Authors may use the Outcome Reporting Bias in Trials (ORBIT) classification system.[4] A sensitivity analysis to assess the impact of selective reporting on meta-analytic results may also be considered.[174] In eight of 28 Cochrane reviews published in March 2010, authors did not assess outcome reporting bias; in 16 reviews, authors did assess this bias using the published report; and in the remaining reviews, trial protocols were used.[175] In another study, after investigators applied sensitivity analyses to adjust for outcome reporting bias in 81 Cochrane reviews, the treatment effect estimate was reduced by 20% or more in 19 (23%) of the meta-analyses.[4]

Both publication bias and outcome reporting bias may affect meta-analyses, and the effect can be unpredictable. Adding unreported data from both published and unpublished drug trials to 41 meta-analyses caused 46% of the meta-analytic effect

For peer review only - http://bmjopen.bmj.com/site/about/guidelines.xhtml

No commercial reuse: See rights and reprints http://www.bmj.com/permissions Subscribe: http://www.bmj.com/subscribe

Box 2: Meta-bias caused by selective publication of studies and selective reporting within studies

Systematic reviews aim to synthesise the results of all relevant studies. However, some studies may not be published, and a subset of outcomes and analyses may be incompletely, inadequately, or selectively reported in a published article, based on the results (such as statistical significance, magnitude, or direction of effect). The validity of systematic reviews may be threatened if the outcome data available to reviewers comprise a biased selection of all data that actually exists.[181,182] Such biases are termed meta-biases, meaning that they occur independent of procedural problems during the conduct of a primary study as do typical methodological biases (such as inappropriate method of random sequence generation in randomized trials).[164]

Publication or dissemination bias—Several systematic reviews of empirical studies have found that clinical trials with statistically significant (P<0.05) or positive results are more likely to be published than those with non-significant or negative results.[2,165,183] Investigators' decisions not to submit papers with negative results for publication, rather than editors' rejection of such papers, tend to be the main source of publication bias.[184] However, the decision to write up a study for publication may be influenced by pressure from study sponsors and journal editor.[185] Studies with statistically significant results also tend to be published earlier than studies with non-significant results.[165] If studies are missing from a systematic review for these reasons, exaggerated results may be produced.

Outcome reporting bias—The selective reporting of outcomes due to their significance, magnitude, or direction is termed outcome reporting bias and has been widely documented across the trial literature.[2] Outcomes specified in the protocol may be completely omitted from the published report. When an outcome is measured using multiple scales or at multiple time points, and analysed in various ways (such as intention-to-treat and per-protocol analysis, unadjusted and adjusted for covariates), the choice of which data to present may be influenced by the results. Non-significant results may be partially reported (such as reporting an effect estimate with no measure of variation), resulting in insufficient data to include in a meta-analysis. All of these examples of selectively reported outcome data in primary studies can bias (and sometimes, overestimate) the results of systematic reviews.[2,7,186]

Empirical evidence of selective outcome reporting bias in trials exists. A systematic review of 16 cohorts of clinical trials comparing outcomes reported in trial protocols with the published reports found that at least one primary outcome was omitted, introduced, or changed in 4-50% of reports.[3] In a landmark study, Chan and colleagues found that statistically significant outcomes had higher odds of being fully reported in trial publications compared with non-significant outcomes for efficacy (pooled odds ratio 2.4 (95% confidence interval 1.4 to 4.0)) and safety (pooled odds ratio 4.7 (1.8 to 12)).[164]

estimates to show lower efficacy of the drug, 7% to show identical efficacy, and 46% to show greater efficacy.[176]

Confidence in cumulative estimate

Item 17. Describe how the strength of the body of evidence will be assessed (such as GRADE)

Example

"The quality of evidence for all outcomes will be judged using the Grading of Recommendations Assessment, Development and Evaluation working group methodology. The quality of evidence will be assessed across the domains of risk of bias, consistency, directness, precision and publication bias. Additional domains may be considered where appropriate. Quality will be adjudicated as high (further research is very unlikely to change our confidence in the estimate of effect), moderate (further research is likely to have an important impact on our confidence in the estimate of effect and may change the estimate), low (further research is very likely to have an important impact on our confidence in the estimate of effect and is likely to change the estimate), or very low (very uncertain about the estimate of effect)."[54]

Explanation

Authors should describe which approach they plan on using to summarize the confidence they have in the resulting body of evidence, ideally using an established and validated approach. The description should include a plan for assessing the risk of bias across studies, inconsistency, imprecision, indirectness, publication bias, and factors that increase the confidence in an effect (such as large effects, dose effect relations, and issues around opposing bias and confounding not explaining an effect or lack thereof) for each outcome that is included in the PICO. The Grading of Recommendations Assessment, Development and Evaluation (GRADE) approach is increasingly recommended.[168]

If no such assessments are planned, the authors should state this with a rationale for why not. Authors should describe whether and how they assess the directness related only to populations (including applicability) who are included in the evidence that is assessed (such as if they extrapolated and for what reasons), so that users of the systematic review can make these judgments later for other populations.[177,178] Authors should specify whether the assessment of the strength of evidence will include studies that are excluded from meta-analysis (if applicable).

"Strength of evidence" and "quality of evidence" have been previously been used interchangeably.

Discussion

We hope this detailed explanatory paper will become a pedagogical document that the entire systematic review community can use. Similarly, we have strived to ensure that the paper is useful to authors seeking guidance in what to include in a protocol of their systematic review. We recommend that authors use this paper when seeking a more complete explanation of each item included in the PRISMA-P checklist. We developed this protocol extension to PRISMA in the hopes that it will improve the reporting of protocols and also simplify the process of reporting a protocol, and registering it with PROSPERO. The development of the PRISMA-P 2015 checklist borrowed heavily from the mandatory items included in PROSPERO. When authors register their protocol on PROSPERO, much of this information is the same as what is recommended when completely reporting a protocol using the PRISMA-P checklist.

Similarly, the intent of using PRISMA-P is to make reporting completed systematic reviews easier for authors. For example, once reviewers have described the methods in detail in their protocol, they may not need to repeat them when reporting the final systematic review results, particularly if there have been no protocol amendments. Providing explicit details about planned review methods in a protocol is essential for clarity, transparency, and future reproducibility, and is in line with emerging journal policies.[18] Authors may also wish to develop a protocol to expand on information reported in PROSPERO. For journals that require a more detailed methods section in completed review articles, authors can easily cut and paste information already in their protocol, change the tense of the wording, and add any necessary documentation about protocol modifications or post-review changes where relevant (more likely in complex reviews such as network meta-analyses).

Protocols are important and provide readers with information about the rationale, question(s), and methods proposed by the systematic reviewers. They should always be made available in the public domain. However, for a variety of reasons, they are not always reported or published. Systematic reviewers may,

For peer review only - http://bmjopen.bmj.com/site/about/guidelines.xhtml

for instance, be unsure of what information should be included in a review protocol—a problem PRISMA-P 2015 aims to solve.

We hope PRISMA-P will help increase the proportion of systematic review protocols being reported and published. Peer reviewers, editors, and other interested readers might also find protocols helpful in their assessment of completed reviews. Comparing protocols with completed reviews enables users to assess possible selective reporting and other possible deviations from the proposed systematic review plan. Investigators completing systematic reviews of systematic reviews (that is, overviews) might also find protocols useful for similar reasons.

We hope that journal editors will encourage authors submitting systematic review protocols for publication to comply with PRISMA-P. We hope funders and sponsors of systematic reviews will do likewise. We also invite readers to let us know what they think of PRISMA-P and ways we can improve it and keep it up to date.

The PRISMA-P steering committee thank the following staff from DM's research group at OHRI: Jodi Peters for her efforts organizing the PRISMA-P consensus meeting; Michael Zhao for his assistance in preparing documents for the PRISMA-P meeting; Mohammed Ansari for valuable input and feedback throughout the process; and Justin Thielman for his assistance collating group comments during preparation of the PRISMA-P manuscripts.

Members of the PRISMA-P group (listed alphabetically): Douglas G Altman, Centre for Statistics in Medicine (CSM), University of Oxford, Oxford, UK; Alison Booth, Centre for Reviews and Dissemination (CRD), University of York, York, UK; An-Wen Chan, Women's College Research Institute, University of Toronto, Toronto, Canada; Stephanie Chang, Agency for Healthcare Research and Quality, Rockville, USA; Mike Clarke, Queen's University of Belfast, Belfast, Ireland; Tammy Clifford, Canadian Agency for Drugs and Technologies in Health (CADTH), Ottawa, Canada; Kay Dickersin, Johns Hopkins Bloomberg School of Public Health; Matthias Egger, Institut für Sozial-und Präventivmedizin; Davina Ghersi, National Health and Medical Research Council, Canberra, Australia; Peter C Gøtzsche, Nordic Cochrane Centre, Copenhagen, Denmark; Jeremy M Grimshaw, Canadian Cochrane Centre and Ottawa Hospital Research Institute (OHRI), Ottawa, Canada; Trish Groves, *The BMJ*, London, UK; Mark Helfand, AHRQ EPC Scientific Resource Center, Portland VA Research Foundation, Portland, USA; Julian Higgins, School of Social and Community Medicine, Bristol, UK; Toby Lasserson, Cochrane Editorial Unit, London, UK; Joseph Lau, Center for Evidence-based Medicine, Brown University, Providence, USA; Alessandro Liberati, University of Modena, Modena, Italy; Kathleen Lohr, Research Triangle Institute-University of North Carolina EPC, Research Triangle Park, USA; Jessie McGowan, University of Ottawa, Ottawa, Canada; David Moher, Clinical Epidemiology Program, OHRI and University of Ottawa, Ottawa, Canada; Cynthia Mulrow, *Annals of Internal Medicine*, San Antonio, USA; Melissa Norton, *PLoS Medicine*, London, UK; Matthew Page, Monash University, Australia; Mark Petticrew, London School of Hygiene and Tropical Medicine, London, UK; Margaret Sampson. Children's Hospital of Eastern Ontario, Ottawa, Canada; Holger Schünemann, McMaster University, Hamilton, Canada; Larissa Shamseer, Clinical Epidemiology Program, OHRI and University of Ottawa, Ottawa, Canada; Paul Shekelle, Southern California EPC, Los Angeles, USA; Iveta Simera, CSM, University of Oxford, Oxford, UK; Lesley A Stewart, CRD, University of York, York, UK; William Summerskill, *The Lancet*, London, UK; Jennifer Tetzlaff, Clinical Epidemiology Program, OHRI, Ottawa, Canada; Thomas A Trikalinos, Center for Evidence-based Medicine, Brown University, Providence, USA; David Tovey, *The Cochrane Library*, London, UK; Lucy Turner, Clinical Epidemiology Program, OHRI, Ottawa, Canada; Evelyn Whitlock, Kaiser Permanente Research Affiliates EPC, Portland, Oregon, USA.

Contributors: DM, LS, MC, DG, AL, MP, PS, and LAS conceived of this paper. DM and LS drafted the article and all authors critically revised it for important intellectual content. All authors approved the final version of this article. DM is the guarantor of this work.

Competing interests: All authors have completed the ICMJE uniform disclosure form at www.icmje.org/coi_disclosure.pdf and declare: (1) support from the Agency for Healthcare Research and Quality, USA (Contract No HHSA 290 2007 10059 I) and the Canadian Institutes for Health Research (Reference No 114369) for this work; this manuscript does not reflect the opinions of either agency; one author, SC, is an employee of AHRQ. (2) No financial relationships with any organisations that might have an interest in the submitted work in the previous three years (3) MC, DG, DM, MP, and LAS are members of the Advisory Board for PROSPERO. TG is the deputy editor of *The BMJ* and was not involved in the handling of or the decision to publish this manuscript.

1 Moher D, Tetzlaff J, Tricco AC, Sampson M, Altman DG. Epidemiology and reporting characteristics of systematic reviews. *PLoS Med* 2007;4:e78.
2 Dwan K, Gamble C, Williamson PR, Kirkham JJ. Systematic review of the empirical evidence of study publication bias and outcome reporting bias—An updated review. *PloS One* 2013;8:e66844.
3 Dwan K, Altman DG, Cresswell L, Blundell M, Gamble CL, Williamson PR. Comparison of protocols and registry entries to published reports for randomised controlled trials. *Cochrane Database Syst Rev* 2011;(1):MR000031.
4 Kirkham JJ, Dwan KM, Altman DG, Gamble C, Dodd S, Smyth R, et al. The impact of outcome reporting bias in randomised controlled trials on a cohort of systematic reviews. *BMJ* 2010;340:c365.
5 Dwan K, Altman DG, Arnaiz JA, Bloom J, Chan AW, Cronin E, et al. Systematic review of the empirical evidence of study publication bias and outcome reporting bias. *PLoS One* 2008;3:e3081.
6 Chan AW, Krleza-Jeric K, Schmid I, Altman DG. Outcome reporting bias in randomized trials funded by the canadian institutes of health research. *Can Med Assoc J* 2004;171:735.
7 Chan AW, Hróbjartsson A, Haahr MT, Gøtzsche PC, Altman DG. Empirical evidence for selective reporting of outcomes in randomized trials: Comparison of protocols to published articles. *JAMA* 2004;291:2457-65.
8 Page MJ, McKenzie JE, Kirkham J, Dwan K, Kramer S, Green S, et al. *Bias due to selective inclusion and reporting of outcomes and analyses in systematic reviews of randomised trials of healthcare interventions* . Cochrane Library, 2014.
9 Page MJ, McKenzie JE, Forbes A. Many scenarios exist for selective inclusion and reporting of results in randomized trials and systematic reviews. *J Clin Epidemiol* 2013;66:524-37.
10 Kirkham JJ, Altman DG, Williamson PR. Bias due to changes in specified outcomes during the systematic review process. *PLoS One* 2010;5:e9810.
11 Booth A, Clarke M, Ghersi D, Moher D, Petticrew M, Stewart L. Establishing a minimum dataset for prospective registration of systematic reviews: An international consultation. *PLoS One* 2011;6:e27319.
12 Moher D, Liberati A, Tetzlaff J, Altman DG, PRISMA Group. Preferred reporting items for systematic reviews and meta-analyses: The PRISMA statement. *BMJ* 2009;339:b2535.
13 Chan A, Tetzlaff JM, Gøtzsche PS, Altman DG, Mann H, Berlin JA, et al. SPIRIT 2013 explanation and elaboration: Guidance for protocols of clinical trials. *BMJ* 2013;346:e7586.
14 Institute of Medicine. *Finding what works in health care: standards for systematic reviews* . National Academies Press, 2011.
15 Moher D, Shamseer L, Clarke M, Ghersi D, Liberati A, Petticrew M, et al. Preferred reporting items for systematic review and meta-analysis protocols (PRISMA-P) 2015 statement. *Syst Rev* 2015;4:1.
16 Moher D, Schulz KF, Simera I, Altman DG. Guidance for developers of health research reporting guidelines. *PLoS Med* 2010;7:e1000217.
17 Liberati A, Altman DG, Tetzlaff J, Mulrow C, Gotzsche PC, Ioannidis JP, et al. The PRISMA statement for reporting systematic reviews and meta-analyses of studies that evaluate health care interventions: explanation and elaboration. *PLoS Med* 2009;6:e1000100.
18 Enhancing reproducibility. *Nature Methods* 2013;10:367.
19 Beller EM, Glasziou PP, Altman DG, Hopewell S, Bastian H, Chalmers I, et al. PRISMA for abstracts: reporting systematic reviews in journal and conference abstracts. *PLoS Med* 2013;10:e1001419.
20 Mans CM, Reeve JC, Gasparini CA, Elkins MR. Postoperative outcomes following preoperative inspiratory muscle training in patients undergoing open cardiothoracic or upper abdominal surgery: protocol for a systematic review. *Syst Rev* 2012;1:63.
21 Wen J, Ren Y, Wang L, Li Y, Liu Y, Zhou M, et al. The reporting quality of meta-analyses improves: a random sampling study. *J Clin Epidemiol* 2008;61:770.
22 Moher D, Booth A, Stewart L. How to reduce unnecessary duplication: Use PROSPERO. *BJOG* 2014;121:784-6.
23 Montori VM, Wilczynski NL, Morgan D, Haynes RB. Optimal search strategies for retrieving systematic reviews from medline: analytical survey. *BMJ* 2005;330:68.
24 O'Connor AM, Auvermann BW, Higgins JP, Kirychuk SP, Sargeant JM, Von Essen SG, et al. The association between proximity to animal-feeding operations and community health: a protocol for updating a systematic review. *Syst Rev* 2014;3:99.
25 Moher D, Tsertsvadze A. Systematic reviews: When is an update an update? *Lancet* 2006;367:881-3.
26 Shojania KG, Sampson M, Ansari MT, Ji J, Doucette S, Moher D. How quickly do systematic reviews go out of date? A survival analysis. *Ann Intern Med* 2007;147:224-33.
27 Moher D, Tsertsvadze A, Tricco AC, Eccles M, Grimshaw J, Sampson M, et al. A systematic review identified few methods and strategies describing when and how to update systematic reviews. *J Clin Epidemiol* 2007;60:1095.
28 Garritty C, Tsertsvadze A, Tricco AC, Sampson M, Moher D. Updating systematic reviews: an international survey. *PloS One* 2010;5:e9914.
29 Chung M, Newberry SJ, Ansari MT, Yu WW, Wu H, Lee J, et al. Two methods provide similar signals for the need to update systematic reviews. *J Clin Epidemiol* 2012;65:660-8.

For peer review only - http://bmjopen.bmj.com/site/about/guidelines.xhtml

30 Ahmadzai N, Newberry SJ, Maglione MA, Tsertsvadze A, Ansari MT, Hempel S, et al. A surveillance system to assess the need for updating systematic reviews. *Syst Rev* 2013;2:1-16.
31 Chalmers I, Enkin M, Keirse MJ. Preparing and updating systematic reviews of randomized controlled trials of health care. *Milbank Q* 1993:411-37.
32 Cancelliere C, Cassidy JD, Côté P, Hincapié CA, Hartvigsen J, Carroll LJ, et al. Protocol for a systematic review of prognosis after mild traumatic brain injury: an update of the WHO collaborating centre task force findings. *Syst Rev* 2012;1:17.
33 Stewart L, Moher D, Shekelle P. Why prospective registration of systematic reviews makes sense. *Syst Rev* 2012;1:7.
34 Booth A, Clarke M, Ghersi D, Moher D, Petticrew M, Stewart L. An international registry of systematic-review protocols. *Lancet* 2011;377:108-9.
35 Booth A, Clarke M, Dooley G, Ghersi D, Moher D, Petticrew M, et al. The nuts and bolts of PROSPERO: An international prospective register of systematic reviews. *Syst Rev* 2012;1:2.
36 Silagy CA, Middleton P, Hopewell S. Publishing protocols of systematic reviews: comparing what was done to what was planned. *JAMA* 2002;287:2831-4.
37 Siontis KC, Hernandez-Boussard T, Ioannidis JP. Overlapping meta-analyses on the same topic: survey of published studies. *BMJ* 2013;347:f4501.
38 Moher D. The problem of duplicate systematic reviews. *BMJ* 2013;347:f5040.
39 The PLoS ME. Best practice in systematic reviews: the importance of protocols and registration. *PLoS Med* 2011;8:e1001009.
40 Chien PFW, Khan KS, Siassakos D. Registration of systematic reviews: PROSPERO. *BJOG* 2012;119:903-5.
41 Bambra C, Hillier F, Moore H, Summerbell C. Tackling inequalities in obesity: a protocol for a systematic review of the effectiveness of public health interventions at reducing socioeconomic inequalities in obesity amongst children. *Syst Rev* 2012;1:16.
42 Mowatt G, Shirran L, Grimshaw JM, Rennie D, Flanagin A, Yank V, et al. Prevalence of honorary and ghost authorship in cochrane reviews. *JAMA* 2002;287:2769-71.
43 Rennie D, Yank V, Emanuel L. When authorship fails. *JAMA* 1997;278:579-85.
44 Sismondo S. Ghost management: How much of the medical literature is shaped behind the scenes by the pharmaceutical industry? *PLoS Med* 2007;4:e286.
45 Ross JS, Hill KP, Egilman DS, Krumholz HM. Guest authorship and ghostwriting in publications related to rofecoxib. *JAMA* 2008;299:1800-12.
46 Smith R. Maintaining the integrity of the scientific record. *BMJ* 2001;323:588.
47 Ensor J, Riley RD, Moore D, Bayliss S, Jowett S, Fitzmaurice DA. Protocol for a systematic review of prognostic models for the recurrence of venous thromboembolism (VTE) following treatment for a first unprovoked VTE. *Syst Rev* 2013;2:91.
48 Smith R. Authorship is dying: long live contributorship. *BMJ* 1997;315:696.
49 Uniform requirements for manuscripts submitted to biomedical journals: writing and editing for biomedical publication. 2010. www.ICMJE.org.
50 AHRQ Agency for Healthcare Research and Quality, Effective Health Care Program. Research protocol–Apr 3, 2014: Treatments for fibromyalgia in adult subgroups. http://effectivehealthcare.ahrq.gov/search-for-guides-reviews-and-reports/?pageaction=displayproduct&productid=1887#8696.
51 AHRQ Agency for Healthcare Research and Quality, Effective Health Care Program. Research Protocol–Jul 21, 2014: Treatment of non-metastatic muscle-invasive bladder cancer. http://effectivehealthcare.ahrq.gov/search-for-guides-reviews-and-reports/?pageaction=displayproduct&productid=1940#9004.
52 Getz KA, Zuckerman R, Cropp AB, Hindle AL, Krauss R, Kaitin KI. Measuring the incidence, causes, and repercussions of protocol amendments. *Drug Inf J* 2011;45:265-75.
53 Higgins JPT, Green S, eds. Guide to the contents of a Cochrane protocol and review. In: *The Cochrane handbook for systematic reviews of interventions* . Wiley-Blackwell, 2011.
54 Young M, Stevens A, Porath-Waller A, Pirie T, Garritty C, Skidmore B, et al. Effectiveness of brief interventions as part of the screening, brief intervention and referral to treatment (SBIRT) model for reducing the non-medical use of psychoactive substances: a systematic review protocol. *Syst Rev* 2012;1:22.
55 Lundh A, Sismondo S, Lexchin J, Busuioc OA, Bero L. Industry sponsorship and research outcome. *Cochrane Database Syst Rev* 2012;(12):MR000033.
56 Bero L. Industry sponsorship and research outcome: a cochrane review. *JAMA Intern Med* 2013;173:580-1.
57 Roseman M, Turner EH, Lexchin J, Coyne JC, Bero LA, Thombs BD. Reporting of conflicts of interest from drug trials in Cochrane reviews: cross sectional study. *BMJ* 2012;345:e5155.
58 Jorgensen AW, Hilden J, Gotzsche PC. Cochrane reviews compared with industry supported meta-analyses and other meta-analyses of the same drugs: systematic review. *BMJ* 2006;333:782.
59 Yank V, Rennie D, Bero LA. Financial ties and concordance between results and conclusions in meta-analyses: retrospective cohort study. *BMJ* 2007;335:1202-5.
60 Jorgensen AW, Maric KL, Tendal B, Faurschou A, Gotzsche PC. Industry-supported meta-analyses compared with meta-analyses with non-profit or no support: differences in methodological quality and conclusions. *BMC Med Res Methodol* 2008;8:60.
61 Bes-Rastrollo M, Schulze MB, Ruiz-Canela M, Martinez-Gonzalez MA. Financial conflicts of interest and reporting bias regarding the association between sugar-sweetened beverages and weight gain: A systematic review of systematic reviews. *PLoS Med* 2013;10:e1001578.
62 Rochon PA, Hoey J, Chan A, Ferris LE, Lexchin J, Kalkar SR, et al. Financial conflicts of interest checklist 2010 for clinical research studies. *Open Med* 2010;4:e69.
63 Holden MA, Haywood KL, Potia TA, Gee M, McLean S. Recommendations for exercise adherence measures in musculoskeletal settings: a systematic review and consensus meeting (protocol). *Syst Rev* 2014;3:1-6.
64 Founti P, Topouzis F, Anastasopoulos E, Pappas T, Lambropoulos A, Chatzikyriakidou A, et al. Association of LOXL1 polymorphisms with pseudoexfoliation syndrome and pseudoexfoliative glaucoma: systematic review and meta-analysis. PROSPERO:CRD42014009228. www.crd.york.ac.uk/PROSPERO/display_record.asp?ID=CRD42014009228.
65 CDISC Clinical Data Interchange Standards Consortium. *CDISC Clinical Research Glossary* . 2011. www.cdisc.org/stuff/contentmgr/files/0/08a36984bc61034baed3b019f3a87139/misc/act1211_011_043_gr_glossary.pdf.
66 Hayden J, Cartwright J, Riley R, vanTulder M. Exercise therapy for chronic low back pain: Protocol for an individual participant data meta-analysis. *Syst Rev* 2012;1:64.
67 Moseley AM, Elkins MR, Herbert RD, Maher CG, Sherrington C. Cochrane reviews used more rigorous methods than non-cochrane reviews: survey of systematic reviews in physiotherapy. *J Clin Epidemiol* 2009;62:1021-30.
68 Psaty BM, Kronmal RA. Reporting mortality findings in trials of rofecoxib for alzheimer disease or cognitive impairment: A case study based on documents from rofecoxib litigation. *JAMA* 2008;299:1813-7.
69 Cohen D. Rosiglitazone: What went wrong? *BMJ* 2010;341:c4848.
70 Chung A, Backholer K, Wong E, Palermo C, Keating C, Peeters A. Trends in child and adolescent obesity prevalence according to socioeconomic position: Protocol for a systematic review. *Syst Rev* 2014;3:1-4.
71 Moher D, Tsertsvadze A, Tricco AC, Eccles MG, J., Sampson M, Barrowman N. A systematic review identified few methods and strategies describing when and how to update systematic reviews. *J Clin Epidemiol* 2007;60:1095.
72 Eipe N, Penning J, Ansari M, Yazdi F, Ahmadzai N. A protocol for a systematic review for perioperative pregabalin use. *Syst Rev* 2012;1:40.
73 Honein-Abouhaidar GN, Kastner M, Vuong V, Perrier L, Rabeneck L, Tinmouth J, et al. Benefits and barriers to participation in colorectal cancer screening: A protocol for a systematic review and synthesis of qualitative studies. *BMJ Open* 2014;4:e004508.
74 Counsell C. Formulating questions and locating primary studies for inclusion in systematic reviews. *Ann Intern Med* 1997;127:380-7.
75 Whitlock EP, Lopez SA, Chang S, Helfand M, Eder M, Floyd N. AHRQ series paper 3: Identifying, selecting, and refining topics for comparative effectiveness systematic reviews: AHRQ and the effective health-care program. *J Clin Epidemiol* 2010;63:491.
76 Hamel C, Stevens A, Singh K, Ansari MT, Myers E, Ziegler P, et al. Do sugar-sweetened beverages cause adverse health outcomes in adults? A systematic review protocol. *Syst Rev* 2014;3:108.
77 Higgins JPT and Green S, eds. *Cochrane handbook for systematic reviews of interventions* . version 5.0.2. Cochrane Collaboration, 2009.
78 Guyatt GH, Oxman AD, Montori V, Vist G, Kunz R, Brozek J, et al. GRADE guidelines: 5. rating the quality of evidence-publication bias. *J Clin Epidemiol* 2011;64:1277-82.
79 Adams D, Wu T, Yasui Y, Aung S, Vohra S. Systematic reviews of TCM trials: How does inclusion of Chinese trials affect outcome? *J Evid Based Med* 2012;5:89-97.
80 Hopewell S, McDonald S, Clarke Mike J, Egger M. *Grey literature in meta-analyses of randomized trials of health care interventions* . John Wiley & Sons, 2007.
81 Van Enst WA, Scholten RJPM, Hooft L. Identification of additional trials in prospective trial registers for cochrane systematic reviews. *PloS One* 2012;7:e42812.
82 Tricco A, Chit A, Hallett D, Soobiah C, Meier G, Chen M, et al. Effect of influenza vaccines against mismatched strains: A systematic review protocol. *Syst Rev* 2012;1:35.
83 Horsley T, Dingwall O, Sampson M. Checking reference lists to find additional studies for systematic reviews. *Cochrane Database Syst Rev* 2011;(8):MR000026.
84 Zhang L, Sampson M, McGowan J. Reporting of the role of the expert searcher in cochrane reviews. *Evid Based Libr Inf Pract* 2006;1:3-16.
85 Lefebvre C, Manheimer E, Glanville J, Cochrane Information Retrieval Methods Group. Searching for studies. In: Higgins JPT, Green S, eds. *Cochrane handbook for systematic reviews of interventions* . 5.1.0 ed. Cochrane Collaboration, 2011.
86 Relevo R, Balshem H. Finding evidence for comparing medical interventions. In: *Methods guide for effectiveness and comparative effectiveness reviews* . AHRQ Publication No 10(12)-EHC063-EF. Agency for Healthcare Research and Quality, 2012.
87 Daboval T, Ward N, Sampson M, Kharrat A. Antenatal counseling about the perinatal care plan at the limit of viability: What do parents want? A systematic review protocol. (unpublished).
88 McGowan J, Sampson M, Lefebvre C. An evidence based checklist for the peer review of electronic search strategies (PRESS EBC). *Evid Based Libr Inf Pract* 2010;5:149-54.
89 Deeks JJ, Altman DG. Inadequate reporting of controlled trials as short reports. *Lancet* 1998;352:1908.
90 Iansavichene AE, Sampson M, McGowan J, Ajiferuke ISY. Should systematic reviewers search for randomized, controlled trials published as letters? *Ann Intern Med* 2008;148:714-5.
91 Sampson M, McGowan J, Cogo E, Grimshaw J, Moher D, Lefebvre C. An evidence-based practice guideline for the peer review of electronic search strategies. *J Clin Epidemiol* 2009;62:944-52.
92 Elamin MB, Flynn DN, Bassler D, Briel M, Alonso-Coello P, Karanicolas PJ, et al. Choice of data extraction tools for systematic reviews depends on resources and review complexity. *J Clin Epidemiol* 2009;62:506-10.
93 Tramèr MR, Reynolds DJM, Moore RA, McQuay HJ. Impact of covert duplicate publication on meta-analysis: a case study. *BMJ* 1997;315:635-40.
94 Von Elm E, Poglia G, Walder B, Tramer MR. Different patterns of duplicate publication. *JAMA* 2004;291:974-80.
95 Gøtzsche P. Multiple publication of reports of drug trials. *Eur J Clin Pharmacol* 1989;36:429-32.
96 Macdonald G, McCartan CJ. Centre-based early education interventions for improving school readiness. *Cochrane Database Syst Rev* 2014;(1):CD010913.
97 Edwards P, Clarke M, DiGuiseppi C, Pratap S, Roberts I, Wentz R. Identification of randomized controlled trials in systematic reviews: Accuracy and reliability of screening records. *Stat Med* 2002;21:1635-40.
98 Cooper H, Ribble RG. Influences on the outcome of literature searches for integrative research reviews. *Sci Commun* 1989;10:179-201.
99 Busse JW, Ebrahim S, Connell G, Coomes EA, Bruno P, Malik K, et al. Systematic review and network meta-analysis of interventions for fibromyalgia: A protocol. *Syst Rev* 2013;2:18.
100 Buscemi N, Hartling L, Vandermeer B, Tjosvold L, Klassen TP. Single data extraction generated more errors than double data extraction in systematic reviews. *J Clin Epidemiol* 2006;59:697-703.
101 Horton J, Vandermeer B, Hartling L, Tjosvold L, Klassen TP, Buscemi N. Systematic review data extraction: Cross-sectional study showed that experience did not increase accuracy. *J Clin Epidemiol* 2010;63:289-98.
102 Jones AP, Remmington T, Williamson PR, Ashby D, Smyth RL. High prevalence but low impact of data extraction and reporting errors were found in cochrane systematic reviews. *J Clin Epidemiol* 2005;58:741-2.
103 Shadish WR, Brasil ICC, Illingworth DA, White KD, Galindo R, Nagler ED, et al. Using UnGraph to extract data from image files: Verification of reliability and validity. *Behav Res Methods* 2009;41:177-83.
104 Stewart LA. Practical methodology of meta?analyses (overviews) using updated individual patient data. *Stat Med* 2007;14:2057-79.
105 Clarke M, Hopewell S, Juszczak E, Eisinga A, Kjeldstrom M. Compression stockings for preventing deep vein thrombosis in airline passengers. *Cochrane Database Syst Rev* 2006;(2):CD004002.

For peer review only

No commercial reuse: See rights and reprints http://www.bmj.com/permissions
Subscribe: http://www.bmj.com/subscribe

106 Glasziou P, Meats E, Heneghan C, Shepperd S. What is missing from descriptions of treatment in trials and reviews? *BMJ* 2008;336:1472.
107 Schulz KF, Altman DG, Moher D. CONSORT 2010 statement: Updated guidelines for reporting parallel group randomised trials. *BMC Med* 2010;8:18.
108 Higgins JPT, Deeks JJ. Selecting studies and collecting data. In: Higgins JPT, Green S, eds. *The Cochrane handbook for systematic reviews of interventions* . 5.1.0 ed. Cochrane Collaboration, 2011.
109 Errami M, Sun Z, Long TC, George AC, Garner HR. Deja vu: A database of highly similar citations in the scientific literature. *Nucleic Acids Res* 2009;37(suppl 1):D921-4.
110 Reichenbach S, Rutjes AWS, Nüesch E, Trelle S, Jüni P. Arthroscopic lavage for osteoarthritis of the knee. Cochrane Library. 2008, doi:10.1002/14651858.CD007320.
111 Tully PJ, Baumeister H. Collaborative care for the treatment of comorbid depression and coronary heart disease: A systematic review and meta-analysis protocol. *Syst Rev* 2014;3:127.
112 Dwan K, Gamble C, Kolamunnage-Dona R, Mohammed S, Powell C, Williamson P. Assessing the potential for outcome reporting bias in a review: A tutorial. *Trials* 2010;11:52.
113 Leucht C, Huhn M, Leucht S. Amitriptyline versus placebo for major depressive disorder (protocol). *Cochrane Database Syst Rev* 2011;(5):CD009138.
114 Helfand M, Balshem H. AHRQ series paper 2: Principles for developing guidance: AHRQ and the effective health-care program. *J Clin Epidemiol* 2010;63:484.
115 Johns MW. A new method for measuring daytime sleepiness: The Epworth sleepiness scale. *Sleep* 1991;14:540-5.
116 Johns MW. Reliability and factor analysis of the Epworth sleepiness scale. *Sleep* 1992;15:376-81.
117 Selby JV, Beal AC, Frank L. The Patient-Centered Outcomes Research Institute (PCORI) national priorities for research and initial research agenda. *JAMA* 2012;307:1583-4.
118 Methodology Committee of the Patient-Centered Outcomes Research Institute (PCORI). Methodological standards and patient-centeredness in comparative effectiveness research: The PCORI perspective. *JAMA* 2012;307:1636-40.
119 Cordoba G, Schwartz L, Woloshin S, Bae H, Gotzsche PC. Definition, reporting, and interpretation of composite outcomes in clinical trials: systematic review. *BMJ* 2010;341:c3920.
120 Page MJ, McKenzie JE, Forbes A. Many scenarios exist for selective inclusion and reporting of results in randomized trials and systematic reviews. *J Clin Epidemiol* 2013;66:524-37.
121 Hosseini Araghi M, Chen YF, Jagielski A, Mannan Choudhury S, Banerjee D, Thomas GN, et al. Weight loss intervention through lifestyle modification or pharmacotherapy for obstructive sleep apnoea in adults (protocol). *Cochrane Database Syst Rev* 2012;(12):CD010281.
122 Lawley CM, Lain SJ, Algert CS, Ford JB, Figtree GA, Roberts CL. Prosthetic heart valves in pregnancy: A systematic review and meta-analysis protocol. *Syst Rev* 2014;3:8.
123 Higgins JPT, Altman DG, Gøtzsche PC, Jüni P, Moher D, Oxman AD, et al. The Cochrane Collaboration's tool for assessing risk of bias in randomised trials. *BMJ* 2011;343:d5928.
124 Viswanathan M, Ansari MT, Berkman ND, Chang S, Hartling L, McPheeters M, et al. Assessing the risk of bias of individual studies in systematic reviews of health care interventions. *AHRQ Methods for Effective Health Care* 2012 Mar 08.
125 Hartling L, Hamm MP, Milne A, Vandermeer B, Santaguida PL, Ansari M, et al. Testing the risk of bias tool showed low reliability between individual reviewers and across consensus assessments of reviewer pairs. *J Clin Epidemiol* 2013;66:973-81.
126 Jadad AR, Moore RA, Carroll D, Jenkinson C, Reynolds DJ, Gavaghan DJ, et al. Assessing the quality of reports of randomized clinical trials: Is blinding necessary? *Control Clin Trials* 1996;17:1-12.
127 Berlin JA. Does blinding of readers affect the results of meta-analyses? *Lancet* 1997;350:185-6.
128 Moja LP, Telaro E, D'Amico R, Moschetti I, Coe L, Liberati A. Assessment of methodological quality of primary studies by systematic reviews: Results of the meta-quality cross sectional study. *BMJ* 2005;330:1053.
129 Wood L, Egger M, Gluud LL, Schulz KF, Jüni P, Altman DG, et al. Empirical evidence of bias in treatment effect estimates in controlled trials with different interventions and outcomes: Meta-epidemiological study. *BMJ* 2008;336:601-5.
130 Savović J, Harris RJ, Wood L, Beynon R, Altman D, Als-Nielsen B, et al. Development of a combined database for meta-epidemiological research. *Res Synth Methods* 2011;2:78.
131 Dechartres A, Boutron I, Trinquart L, Charles P, Ravaud P. Single-center trials show larger treatment effects than multicenter trials: Evidence from a meta-epidemiologic study. *Ann Intern Med* 2011;155:39.
132 Odgaard-Jensen J, Vist G, Timmer A, Kunz R, Akl E, Schünemann H, et al. Randomised controlled trials as a safeguard against biased estimates of treatment effects. *Cochrane Database Syst Rev* 2011;(4):MR000012.
133 Sanderson S, Tatt ID, Higgins JPT. Tools for assessing quality and susceptibility to bias in observational studies in epidemiology: A systematic review and annotated bibliography. *Int J Epidemiol* 2007;36:666-76.
134 Dechartres, A. Charles, P. Hopewell, S. Ravaud, P. Altman, D.G. Reviews assessing the quality or the reporting of randomized controlled trials are increasing over time but raised questions about how quality is assessed. *J Clin Epidemiol* 2011;64:136-44.
135 Jüni P, Witschi A, Bloch R, Egger M. The hazards of scoring the quality of clinical trials for meta-analysis. *JAMA* 1999;282:1054-60.
136 Ioannidis JPA, Haidich AB, Pappa M, Pantazis N, Kokori SI, Tektonidou MG, et al. Comparison of evidence of treatment effects in randomized and nonrandomized studies. *JAMA* 2001;286:821-30.
137 Savovic J, Jones HE, Altman DG, Harris RJ, Juni P, Pildal J, et al. Influence of reported study design characteristics on intervention effect estimates from randomized, controlled trials. *Ann Intern Med* 2012;157:429-38.
138 Hróbjartsson A, Thomsen ASS, Emanuelsson F, Tendal B, Hilden J, Boutron I, et al. Observer bias in randomised clinical trials with binary outcomes: Systematic review of trials with both blinded and non-blinded outcome assessors. *BMJ* 2012;344:e1119.
139 Bell ML, Kenward MG, Fairclough DL, Horton NJ. Differential dropout and bias in randomised controlled trials: When it matters and when it may not. *BMJ* 2013;346:e8668.
140 Guyatt GH, Briel M, Glasziou P, Bassler D, Montori VM. Problems of stopping trials early. *BMJ* 2012;344:e3863.
141 Briel M, Bassler D, Wang AT, Guyatt GH, Montori VM. The dangers of stopping a trial too early. *J Bone Joint Surg* 2012;94(suppl 1):56-60.
142 DeAngelis CD FP. Impugning the integrity of medical science: The adverse effects of industry influence. *JAMA* 2008;299:1833-5.
143 Kahan BC, Morris TP. Analysis of multicentre trials with continuous outcomes: When and how should we account for centre effects? *Stat Med* 2013;32:1136-49.
144 Shephard RJ. Ethics in exercise science research. *Sports Med* 2002;32:169-83.
145 Fanelli D. How many scientists fabricate and falsify research? A systematic review and meta-analysis of survey data. *PLoS One* 2009;4:e5738.
146 Higgins J, Ramsay C, Reeves BC, Deeks JJ, Shea B, Valentine JC, et al. Issues relating to study design and risk of bias when including non-randomized studies in systematic reviews on the effects of interventions. *Res Synth Methods* 2012.
147 Mao C, Yang ZY, Chung VCH, Qin Y, Tam W, Kwong JSW, et al. Tong-xin-luo capsule for patients with coronary heart disease after percutaneous coronary intervention. *Cochrane Library* 2012.
148 Villar J, Mackey ME, Carroli G, Donner A. Meta-analyses in systematic reviews of randomized controlled trials in perinatal medicine: Comparison of fixed and random effects models. *Stat Med* 2001;20:3635-47.
149 Hunter JE, Schmidt FL. Fixed effects vs. random effects meta-analysis models: implications for cumulative research knowledge. *Int J Select Assess* 2002;8:275-92.
150 Schmidt FL, Oh IS, Hayes TL. Fixed- versus random-effects models in meta-analysis: model properties and an empirical comparison of differences in results. *Br J Math Stat Psychol* 2009;62:97-128.
151 Higgins J, Thompson SG, Deeks JJ, Altman DG. Measuring inconsistency in meta-analyses. *BMJ* 2003;327:557-60.
152 Higgins JPT, Deeks JJ, Altman DG. Special topics in statistics. In: Higgins JPT, Green S, eds. *Cochrane handbook for systematic reviews of interventions* . 5.1.0 ed. Cochrane Collaboration, 2011.
153 Stewart LA, Clarke M, Rovers M, Riley RD, Simmonds M, Stewart G, et al. PRISMA-IPD: an extension to the PRISMA statement guidance for reporting systematic review and meta-analysis of individual participant data. (submitted).
154 Thiessen PH, Barrowman N, Garg A. Imputing variance estimates do not alter the conclusions of a meta-analysis with continuous outcomes: A case study of changes in renal function after living kidney donation. *J Clin Epidemiol* 2007;60:228.
155 Stevens JW. A note on dealing with missing standard errors in meta-analyses of continuous outcome measures in WinBUGS. *Pharmaceut Stat* 2011;10:374-8.
156 Wiebe N, Vandermeer B, Platt RW, Klassen TP, Moher D, Barrowman NJ. A systematic review identifies a lack of standardization in methods for handling missing variance data. *J Clin Epidemiol* 2006;59:342-53.
157 Furukawa TA, Barbui C, Cipriani A, Brambilla P, Watanabe N. Imputing missing standard deviations in meta-analyses can provide accurate results. *J Clin Epidemiol* 2006;59:7.
158 Tierney JF, Stewart LA, Ghersi D, Burdett S, Sydes MR. Practical methods for incorporating summary time-to-event data into meta-analysis. *Trials* 2007;8:16.
159 Wood L, Egger M, Gluud LL, Schulz KF, Jüni P, Altman DG, et al. Empirical evidence of bias in treatment effect estimates in controlled trials with different interventions and outcomes: meta-epidemiological study. *BMJ* 2008;336:601-5.
160 Whitehead PJ, Drummond AE, Walker MF, Parry RH. Interventions to reduce dependency in personal activities of daily living in community-dwelling adults who use homecare services: protocol for a systematic review. *Syst Rev* 2013;2:1-7.
161 Greenhalgh T, Robert G, Macfarlane F, Bate P, Kyriakidou O, Peacock R. Storylines of research in diffusion of innovation: a meta-narrative approach to systematic review. *Soc Sci Med* 2005;61:417-30.
162 Popay J, Roberts H, Sowden A, Petticrew M, Arai L, Rodgers M, et al. Guidance on the conduct of narrative synthesis in systematic reviews. a product from the ESRC methods programme. Version 1. ESRC, 2006.
163 Richards BL, Whittle SL, Buchbinder R. Neuromodulators for pain management in rheumatoid arthritis (protocol). *Cochrane Database Syst Rev* 2011;(1):CD008921.
164 Goodman S, Dickersin K. Metabias: A challenge for comparative effectiveness research. *Ann Intern Med* 2011;155:61-2.
165 Song F, Parekh S, Hooper L, Loke Y, Ryder J, Sutton A, et al. *Dissemination and publication of research findings: an updated review of related biases* . Prepress Projects, 2010.
166 Sterne JAC, Sutton AJ, Ioannidis JPA, Terrin N, Jones DR, Lau J, et al. Recommendations for examining and interpreting funnel plot asymmetry in meta-analyses of randomised controlled trials. *BMJ* 2011;343:302.
167 Terrin N, Schmid CH, Lau J. In an empirical evaluation of the funnel plot, researchers could not visually identify publication bias. *J Clin Epidemiol* 2005;58:894-901.
168 Lau J, Ioannidis J, Terrin N, Schmid CH, Olkin I. The case of the misleading funnel plot. *BMJ* 2006;333:597-600.
169 Parekh-Bhurke S, Kwok CS, Pang C, Hooper L, Loke YK, Ryder JJ, et al. Uptake of methods to deal with publication bias in systematic reviews has increased over time, but there is still much scope for improvement. *J Clin Epidemiol* 2011;64:349-57.
170 Turner EH, Matthews AM, Linardatos E, Tell RA, Rosenthal R. Selective publication of antidepressant trials and its influence on apparent efficacy. *N Engl J Med* 2008;358:252-60.
171 Turner EH, Knoepflmacher D, Shapley L. Publication bias in antipsychotic trials: An analysis of efficacy comparing the published literature to the us food and drug administration database. *PLoS Med* 2012;9:e1001189.
172 Williamson P, Gamble C, Altman D, Hutton J. Outcome selection bias in meta-analysis. *Stat Methods Med Res* 2005;14:515-24.
173 Chan AW, Altman DG. Identifying outcome reporting bias in randomised trials on PubMed: review of publications and survey of authors. *BMJ* 2005;330:753.
174 Williamson PR, Gamble C. Application and investigation of a bound for outcome reporting bias. *Trials* 2007;8:9.
175 Tharyan P, Kirubakaran R, Jabez P. The use of trial protocols to assess risk of bias due to selective reporting in cochrane systematic reviews: a cross-sectional survey. 18th Cochrane Colloquium and 10th Campbell Colloquium; Oct 18-22; Keystone, Colorado; 2010.
176 Hart B, Lundh A, Bero L. Effect of reporting bias on meta-analyses of drug trials: reanalysis of meta-analyses. *BMJ* 2012;344:d7202.
177 Guyatt G, Oxman AD, Akl EA, Kunz R, Vist G, Brozek J, et al. GRADE guidelines: 1. Introduction-GRADE evidence profiles and summary of findings tables. *J Clin Epidemiol* 2011;64:383-94.
178 Guyatt G, Oxman AD, Sultan S, Brozek J, Glasziou P, Alonso-Coello P, et al. GRADE guidelines. 11: Making an overall rating of confidence in effect estimates for a single outcome and for all outcomes. *J Clin Epidemiol* 2013;66:151-7.
179 Antman EM, Lau J, Kupelnick B, Mosteller F, Chalmers TC. A comparison of results of meta-analyses of randomized control trials and recommendations of clinical experts. treatments for myocardial infarction. *JAMA* 1992;268:240-8.
180 Oxman AD, Guyatt GH. The science of reviewing research. *Ann N Y Acad Sci* 1993;703:125.

For peer review only - http://bmjopen.bmj.com/site/about/guidelines.xhtml

181 Dickersin K. The existence of publication bias and risk factors for its occurrence. *JAMA* 1990;263:1385-9.
182 Begg CB. Publication bias: A problem in interpreting medical data. *J R Stat Soc A* 1988;151:419-63.
183 Hopewell S, Loudon K, Clarke MJ, Oxman AD, Dickersin K. Publication bias in clinical trials due to statistical significance or direction of trial results. *Cochrane Database Syst Rev* 2009;(1):MR000006.
184 Dickersin K, Chalmers I. Recognizing, investigating and dealing with incomplete and biased reporting of clinical research: from Francis Bacon to the WHO. *J R Soc Med* 2011;104:532-8.
185 Dickersin K. Publication bias: recognizing the problem, understanding its origins and scope, and preventing harm. In: Rothstein HR, Sutton AJ, Borenstein M, eds. *Publication bias in meta-analysis—Prevention, assessment and adjustments* . John Wiley & Sons, 2005:11-33.
186 Williamson PR, Gamble C, Altman DG, Hutton JL. Outcome selection bias in meta-analysis. *Stat Methods Med Res* 2005;14:515-24.

Cite this as: *BMJ* 2015;350:g7647

This is an Open Access article distributed in accordance with the Creative Commons Attribution Non Commercial (CC BY-NC 4.0) license, which permits others to distribute, remix, adapt, build upon this work non-commercially, and license their derivative works on different terms, provided the original work is properly cited and the use is non-commercial. See: http://creativecommons.org/licenses/by-nc/4.0/

For peer review only

Tables

Table 1| Proposed stakeholders, actions, and potential benefits for supporting adherence to PRISMA-P

Stakeholder	Proposed action	Potential benefits
Funders	Promote or mandate adherence to PRISMA-P or use PRISMA-P as a template for systematic review proposals for grant applications	Improved quality, completeness, and consistency of systematic review proposals<br>Standardized protocol content will improve peer review efficiency and investigator understanding of requirements
Systematic reviewers, groups, or organizations	Use or adhere to PRISMA-P during protocol development	Improved quality, completeness, and consistency of protocol content<br>Enables reviewers to anticipate and avoid future changes to review methods (that is, outcomes)<br>Increased awareness of minimum content for protocol reporting<br>Improved completeness of reporting of completed reviews
PROSPERO (and other review registries)	Encourage the development of PRISMA-P based protocols	Improved quality of registry entries<br>Improved consistency across registry entries, protocols, and systematic reviews
Practice guideline developers	Use PRISMA-P to gauge the completeness of protocols and facilitate detection of selective reporting when considering reviews for guideline inclusion	Enables easy comparison across protocols, registry entries, and completed systematic reviews
Policymakers	Advocate use of PRISMA-P by those funding and conducting systematic reviews	May yield better quality, more complete, and more consistent reviews to inform decision making
Journal editors	Encourage compliance with PRISMA-P for authors submitting protocols for publication<br>Offer PRISMA-P as a template to assist in protocol writing for publication	Improved quality, completeness, and consistency of protocols over those published in journals not endorsing PRISMA-P<br>Increased efficiency in protocol peer and author understanding of journal requirements<br>Improved transparency of reviews and interpretation by readers
Educators	Use PRISMA-P as a training tool<br>Encourage adherence in students submitting protocols for coursework	Simplified teaching and grading of protocols<br>Improved quality, completeness, and consistency of protocol content
Students	Develop protocols for coursework or research using PRISMA-P	Improved understanding of the minimum protocol content<br>Well trained systematic reviewers entering the workforce

For peer review only

No commercial reuse: See rights and reprints http://www.bmj.com/permissions Subscribe: http://www.bmj.com/subscribe

Table 2| PRISMA-P (preferred reporting items for systematic review and meta-analysis protocols) 2015 checklist: recommended items to address in a systematic review protocol

Section and topic	Item No	Checklist item
**Administrative information**		
Title:		
Identification	1a	Identify the report as a protocol of a systematic review
Update	1b	If the protocol is for an update of a previous systematic review, identify as such
Registration	2	If registered, provide the name of the registry (such as PROSPERO) and registration number
Authors:		
Contact	3a	Provide name, institutional affiliation, e-mail address of all protocol authors; provide physical mailing address of corresponding author
Contributions	3b	Describe contributions of protocol authors and identify the guarantor of the review
Amendments	4	If the protocol represents an amendment of a previously completed or published protocol, identify as such and list changes; otherwise, state plan for documenting important protocol amendments
Support:		
Sources	5a	Indicate sources of financial or other support for the review
Sponsor	5b	Provide name for the review funder and/or sponsor
Role of sponsor or funder	5c	Describe roles of funder(s), sponsor(s), and/or institution(s), if any, in developing the protocol
**Introduction**		
Rationale	6	Describe the rationale for the review in the context of what is already known
Objectives	7	Provide an explicit statement of the question(s) the review will address with reference to participants, interventions, comparators, and outcomes (PICO)
**Methods**		
Eligibility criteria	8	Specify the study characteristics (such as PICO, study design, setting, time frame) and report characteristics (such as years considered, language, publication status) to be used as criteria for eligibility for the review
Information sources	9	Describe all intended information sources (such as electronic databases, contact with study authors, trial registers or other grey literature sources) with planned dates of coverage
Search strategy	10	Present draft of search strategy to be used for at least one electronic database, including planned limits, such that it could be repeated
Study records:		
Data management	11a	Describe the mechanism(s) that will be used to manage records and data throughout the review
Selection process	11b	State the process that will be used for selecting studies (such as two independent reviewers) through each phase of the review (that is, screening, eligibility and inclusion in meta-analysis)
Data collection process	11c	Describe planned method of extracting data from reports (such as piloting forms, done independently, in duplicate), any processes for obtaining and confirming data from investigators
Data items	12	List and define all variables for which data will be sought (such as PICO items, funding sources), any pre-planned data assumptions and simplifications
Outcomes and prioritization	13	List and define all outcomes for which data will be sought, including prioritization of main and additional outcomes, with rationale
Risk of bias in individual studies	14	Describe anticipated methods for assessing risk of bias of individual studies, including whether this will be done at the outcome or study level, or both; state how this information will be used in data synthesis
Data synthesis	15a	Describe criteria under which study data will be quantitatively synthesised
	15b	If data are appropriate for quantitative synthesis, describe planned summary measures, methods of handling data and methods of combining data from studies, including any planned exploration of consistency (such as I^2, Kendall's τ)
	15c	Describe any proposed additional analyses (such as sensitivity or subgroup analyses, meta-regression)
	15d	If quantitative synthesis is not appropriate, describe the type of summary planned
Meta-bias(es)	16	Specify any planned assessment of meta-bias(es) (such as publication bias across studies, selective reporting within studies)
Confidence in cumulative evidence	17	Describe how the strength of the body of evidence will be assessed (such as GRADE)

For peer review only

For peer review only - http://bmjopen.bmj.com/site/about/guidelines.xhtml

Table 3| AHRQ process for dealing with protocol amendments. Changes made to the protocol should not be incorporated throughout the various sections of the protocol. Instead, protocol amendments should be noted only in section VII of the protocol, preferably in a tabular format (see example below), and the date of the amendment noted at the top of the protocol (from http://effectivehealthcare.ahrq.gov/index.cfm/search-for-guides-reviews-and-reports/?productid=1724&pageaction=displayproduct)

Date	Section	Original protocol	Revised protocol	Rationale
This should be the effective date of the change in protocol	Specify where the change would be found in the protocol	Describe language of the original protocol	Describe the change in protocol	Justify why the change will improve the report. If necessary, describe why the change does not introduce bias. Do not use justification such as, “because the AE/TOO/TEP/Peer reviewer told us to do so,” but explain what the change hopes to accomplish

For peer review only

No commercial reuse: See rights and reprints http://www.bmj.com/permissions Subscribe: http://www.bmj.com/subscribe

BMJ Open

Delayed antibiotic prescribing for respiratory tract infections: protocol of an individual patient data meta-analysis

Journal:	*BMJ Open*
Manuscript ID	bmjopen-2018-026925.R1
Article Type:	Protocol
Date Submitted by the Author:	05-Nov-2018
Complete List of Authors:	Stuart, Beth; University of Southampton, Academic Unit of Primary Care and Population Sciences, Faculty of Medicine<br>Hounkpatin, Hilda; University of Southampton, Academic Unit of Primary Care and Population Sciences, Faculty of Medicine<br>Becque, Taeko; University of Southampton, Academic Unit of Primary Care and Population Sciences, Faculty of Medicine<br>Yao, Guiqing; University of Leicester, UK, Biostatistics Research Group, Department of Health Sciences, College of Life Sciences<br>Zhu, Shihua; University of Southampton, Academic Unit of Primary Care and Population Sciences, Faculty of Medicine<br>Alonso-Coello, Pablo; Iberoamerican Cochrane Centre, CIBERESP-IIB Sant Pau<br>Altiner, Attila; University of Rostock, Office for Educational Affairs, Department of General Medicine<br>Arroll, Bruce; University of Auckland, Department of General Practice and Primary Health Care<br>Böhning, Dankmar ; University of Southampton, Southampton Statistical Sciences Research Institute<br>Bostock, Jennifer; King's College London, Divison of Health and Social Care Research<br>Bucher, Heiner; University Hospital Basel and University of Basel, Basel Institute for Clinical Epidemiology and Biostatistics (CEB)<br>de la Poza, Mariam; Institut Català de la Salut<br>Francis, Nick A.; Cardiff University, Division of Population Medicine, School of Medicine<br>Gillespie, David<br>Hay, Alastair; University of Bristol, Centre for Academic Primary Care, Population Health Sciences<br>Kenealy, Tim; University of Auckland, General Practice and Primary Health Care<br>Löffler, Christin; Rostock University Medical Center, Institute of General Practice<br>Mas-Dalmau, Gemma; Hospital de la Santa Creu i Sant Pau, Knowledge and research management unit<br>Muñoz, Laura; Agència de Qualitat i Avaluació Sanitàries de Catalunya (AQuAS), Roc Boronat, 81-95, 08005, Barcelona, Spain<br>Samuel, Kirsty; University of Leeds, ASPIRE PPI Panel, Leeds, Institute for Health Sciences<br>Moore, Michael; University of Southampton, Academic Unit of Primary

	Care and Population Sciences, Faculty of Medicine<br>Little, Paul; University of Southampton, Academic Unit of Primary Care and Population Sciences, Faculty of Medicine

**Primary Subject Heading**:	Respiratory medicine
Secondary Subject Heading:	General practice / Family practice, Health services research, Research methods
Keywords:	RESPIRATORY MEDICINE (see Thoracic Medicine), STATISTICS & RESEARCH METHODS, HEALTH ECONOMICS, GENERAL MEDICINE (see Internal Medicine)

SCHOLARONE™
Manuscripts

Delayed antibiotic prescribing for respiratory tract infections: protocol of an individual patient data meta-analysis

Beth Stuart[1*], Hilda Hounkpatin[1*], Taeko Becque[1], Guiqing Yao[2], Shihua Zhu[1], Pablo Alonso-Coello[3], Attila Altiner[4], Bruce Arroll[5], Dankmar Böhning[6], Jennifer Bostock[7], Heiner C. Bucher[8], Mariam de la Poza[9], Nick Francis[10], David Gillespie[11], Alastair Hay[12], Timothy Kenealy[13], Christin Loffler[14], Gemma Mas-Dalmau[15], Laura Muñoz[16], Kirsty Samuel[17], Michael Moore[1] and Paul Little[1]

1. Academic Unit of Primary Care and Population Sciences, Faculty of Medicine, University of Southampton, Southampton, UK
2. Biostatistics Research Group, Department of Health Sciences, College of Life Sciences, University of Leicester, Leiceister, UK
3. Iberoamerican Cochrane Centre, CIBERESP-IIB Sant Pau, Barcelona, Spain
4. Office for Educational Affairs, Department of General Medicine, University of Rostock, 18057 Rostock, Germany
5. Department of General Practice and Primary Health Care, University of Auckland, Auckland, New Zealand
6. Southampton Statistical Sciences Research Institute, University of Southampton, Southampton, SO17 1BJ, UK
7. Division of Health and Social Care Research, King's College London, London, UK
8. Basel Institute for Clinical Epidemiology and Biostatistics (CEB), University Hospital Basel and University of Basel, Switzerland
9. Institut Català de la Salut. CAP Doctor Carles Ribas, Foc 112, Barcelona, Spain
10. Division of Population Medicine, School of Medicine, Cardiff University, Cardiff, South Glamorgan, UK
11. South East Wales Trials Unit (SEWTU), Institute for Translation, Innovation, Methodology and Engagement (TIME), School of Medicine, Cardiff University, UK
12. Centre for Academic Primary Care, Population Health Sciences, Bristol Medical School, University of Bristol, Bristol, UK
13. Department of General Practice and Primary Health Care, University of Auckland, Auckland, New Zealand.
14. Institute of General Practice, Rostock University Medical Centre, Rostock, Germany
15. Knowledge and research management unit, Hospital de la Santa Creu i Sant Pau, Barcelona, Spain
16. Agència de Qualitat i Avaluació Sanitàries de Catalunya (AQuAS), Barcelona, Spain
17. ASPIRE PPI Panel, Leeds, Institute for Health Sciences, University of Leeds, Leeds, UK

Email addresses: Beth Stuart (bls1@soton.ac.uk), Hilda Hounkpatin (H.O.Hounkpatin@soton.ac.uk), Taeko Becque (T.F.Becque@soton.ac.uk), Guiqing Yao (gy38@le.ac.uk), Shihua Zhu (S.Zhu@soton.ac.uk), Pablo Alonso-Coello (palonso@santpau.cat), Attila Altiner (altiner@med.uni-rostock.de), Bruce Arroll (bruce.arroll@auckland.ac.nz), Dankmar Böhning (D.A.Bohning@soton.ac.uk), Jennifer Bostock (jennifer@jlbostock.plus.com), Heiner C. Bucher (heiner.bucher@usb.ch), Mariam de la Poza (mariamdelapoza@gmail.com), Nick Francis (FrancisNA@cardiff.ac.uk), David Gillespie (GillespieD1@cardiff.ac.uk), Alastair Hay (Alastair.Hay@bristol.ac.uk), Timothy Kenealy (t.kenealy@auckland.ac.nz), Christin Loffler (christin.loeffler@med.uni-rostock.de), Gemma Mas-Dalmau (gmasd@santpau.ca), Laura Muñoz (lmunyoz@gmail.com), Kirsty Samuel (akjsamuel@hotmail.com), Michael Moore (mvm198@soton.ac.uk) and Paul Little (P.Little@soton.ac.uk)

*Beth Stuart and Hilda Hounkpatin contributed equally to this paper.

For peer review only

**Corresponding author:
Dr Beth Stuart
Primary Care and Population Sciences Faculty of Medicine University of Southampton Aldermoor Health Centre Aldermoor Close Southampton SO16 5ST
Email: bls1@soton.ac.uk
Telephone number: +44 23 8059 1759
Fax: +44 23 8070 1125

Keywords: Respiratory medicine; Statistics and research methods; Health economics; General medicine.

Word count: 4203

For peer review only

Delayed antibiotic prescribing for respiratory tract infections: protocol of an individual patient data meta-analysis

ABSTRACT

Introduction

Delayed prescribing can be a useful strategy to reduce antibiotic prescribing, but it is not clear for whom delayed prescribing might be effective. This protocol outlines an individual patient data (IPD) meta-analysis of randomised control trials (RCTs) and observational cohort studies to explore the overall effect of delayed prescribing and identify key patient characteristics that are associated with efficacy of delayed prescribing.

Methods and analysis

A systematic search of the databases Cochrane Central Register of Controlled Trials (CENTRAL), Ovid MEDLINE, Ovid Embase, EBSCO CINAHL Plus and Web of Science was conducted to identify relevant studies from inception to October 2017. Outcomes of interest include duration of illness, severity of illness, complication, re-consultation and patient satisfaction. Study authors of eligible papers will be contacted and invited to contribute raw IPD data. IPD data will be checked against published data, harmonised and aggregated to create one large IPD database. Multilevel regression will be performed to explore interaction effects between treatment allocation and patient characteristics. The economic evaluation will be conducted based on individual patient data from the combined trial and observational studies to estimate the differences in costs and effectiveness for delayed prescribing compared to normal practice. A decision model will be developed to assess potential savings and cost-effectiveness in terms of reduced antibiotic usage of delayed prescribing and quality adjusted life years.

Ethics and dissemination

Ethical approval was obtained from the University of Southampton Faculty of Medicine Research Ethics Committee (Reference number: 30068). Findings of this study will be published in peer-reviewed academic journals as well as GP trade journals and will be presented at national and international conferences. The results will have important public health implications, shaping the way in which antibiotics are prescribed in the future and to whom delayed prescriptions are issued.

Word count: 4203

ARTICLE SUMMARY

Strengths and limitations of this study

- This study uses individual patient data (IPD) from randomised controlled trials and observational studies to investigate the clinical effectiveness and potential cost savings of a delayed antibiotic prescribing, both overall and for key subgroups of people.
- IPD provides sufficient statistical power to explore interactions between treatment groups and patient characteristics.
- The IPD will only include data from studies for which the data is available.
- Observational studies will use propensity score approach to adjust for confounding by indication on measured covariates.

For peer review only

- Findings from this study may inform GPs decisions on prescribing and increase use of appropriate delayed prescribing, which in turn can help to reduce antibiotic consumption and antimicrobial resistance.

INTRODUCTION

Antimicrobial resistance is a cause for great concern prompting calls for action at the local, national and international level to prevent "overuse, misuse and abuse" of antibiotics (1), particularly in primary care where antibiotics are most prescribed (2). About 60% of antibiotics prescribed in primary care are for respiratory tract infections (RTIs) (3). However, most infections are self-limiting, symptomatic benefit from antibiotics is modest (4-6) and patients prescribed antibiotics for RTIs are more likely to carry antibiotic resistant commensal bacteria and develop resistant infections (7).

The Standing Medical Advisory Committee report recommends that the fewest number of antibiotic courses should be prescribed for the shortest period possible (8). However, in a primary care context, it can be difficult to tell whether antibiotics are appropriate for an individual patient. It is not always clear which patients are at risk of prolonged illness or developing complications nor whether the RTI is bacterial or viral in nature. Point of care tests such as C reactive protein and clinical scores both show promise in helping to guide GP management decisions (9-12). Delayed prescribing is a useful strategy that may be used on its own or in conjunction with clinical scores and point of care testing. It allows the patient to collect a prescription to be taken if their symptoms do not start to improve within a specific duration of their initial consultation, providing a potential aid to negotiating treatment acceptable to the patient and provider whilst reducing inappropriate prescribing (13,14). Delayed prescribing is recognised as part of the toolkit available to GPs to help reduce antibiotic use, especially in the context of consultations where patients expect to receive an antibiotic prescription and is part of the NICE guidelines (3). A recent large prospective primary care cohort (MRC DESCARTE) demonstrated that delayed prescription was likely to be as effective as immediate antibiotic in reducing complications and more effective at reducing re-consultations (4).

Whilst in some situations delayed antibiotic prescribing is appropriate, for other patients it may be unsuitable and controversial (15). It is important to understand which subgroups of patients may require immediate antibiotics to avoid complications and which patients might benefit from a delayed or no prescribing strategy (4). A 2013 Cochrane review (16) of 10 trials found that a delayed prescribing strategy was not significantly different to immediate prescribing in terms of clinical outcomes for cough and cold. In patients with acute otitis media and sore throat immediate antibiotics were more effective than delayed for reducing fever, pain and malaise in some studies. However, the review noted a high level of heterogeneity between studies made combining them in a traditional meta-analysis difficult and did not allow sufficient power for the examination of subgroups or complications either in meta-analysis or in meta-regression.

Where aggregation is possible, a traditional meta-analysis or even meta-regression can be used to determine the overall main effect of delayed antibiotic prescription. Although such techniques can also be used to explore how effect varies across study characteristics, these techniques lack statistical power and are prone to confounding as patient-level characteristics are not taken into account (17). An individual patient data (IPD) meta-analysis is considered the gold standard for meta-analyses and can overcome the limitations of meta-analysis of aggregate data. In an IPD meta-analysis, original data for all participants in each study are obtained and synthesised. This allows sufficient statistical power (18) to explore interactions between treatment groups and patient characteristics. This would allow us to identify key groups in which delayed prescribing or no prescribing might be inadvisable due to longer durations of illness, greater severity of illness or higher risk of complications/re-consultation. These findings may inform GPs decisions on prescribing

For peer review only

and increase use of appropriate delayed prescribing, which in turn can help to reduce antibiotic consumption and antimicrobial resistance.

This protocol describes an IPD meta-analysis of both randomised controlled trials (RCTs) and observational cohort studies of delayed antibiotic prescribing for acute respiratory infections. Although RCTs represent the 'gold standard' design for assessing the impact of an intervention, the participants in these studies may differ systematically from the patients encountered in every day clinical practice due to the inclusion criteria. By including observational studies in a meta-analysis, with suitable techniques to control for potential confounding by indication, it may be possible to obtain better estimates of the effect of delayed prescribing in routine practice.

METHODS

Aims and objectives

This IPD study will investigate the clinical effectiveness and potential cost savings of a delayed antibiotic prescribing approach, both overall and for key subgroups of patients. The study started in October 2017 and is due to be completed in September 2020.

The objectives of this study are as follows:

- To achieve more accurate estimates of the clinical effectiveness of a delayed prescribing strategy on the primary outcome, symptom severity, by harmonising the outcomes from all trials and observational studies which have included this strategy.
- To estimate the clinical effectiveness of delayed prescribing for secondary outcomes: duration of illness, development of complications, re-consultation and patient satisfaction.
- To explore whether there are key subgroups (informed by the literature and described below) for whom delayed antibiotic prescribing may or may not be beneficial. Subgroups will be: prior duration of illness (above/below median for the condition), age (under 16 years, 16-64 years, age over 65 years), fever at baseline consultation (>37.5 C), comorbid conditions including lung comorbidity such as asthma or chronic obstructive pulmonary disease (COPD), and severity of symptoms at baseline consultation. The effectiveness of delayed antibiotic prescribing for subgroups of symptom complexes such as cough (acute bronchitis), pharyngitis, or otitis media will also be explored.
- To investigate whether symptom trajectories are influenced by antibiotic prescribing/ consumption.
- To estimate the costs of treatment of patients with respiratory tract infections (from the UK National Health Service (NHS) and Personal Social Services (PSS) perspective data) and investigate any potential cost savings and cost effectiveness in terms of antibiotic usage of delayed antibiotic prescriptions compared with antibiotic use.
- To identify priorities for future delayed prescribing research.

Study approach

A full systematic review will be conducted to identify and select eligible papers. Study authors of eligible papers will be contacted and invited to contribute raw data.

Systematic review to identify eligible papers

Eligibility criteria

Design - Randomized controlled trials or eligible observational cohort studies

For peer review only

Population – all patients attending primary, ambulatory or acute care settings with a respiratory tract infection

Intervention – delayed antibiotic prescription

Comparator – immediate antibiotic prescription or no antibiotic prescription

Outcomes – severity of illness (symptom severity), duration of illness, complication, re-consultation, patient satisfaction, costs and quality of life.

Studies on antibiotic prescribing that were not an RCT or observational cohort (for example survey or cross-sectional studies and case-control studies) were excluded. Studies of hospital inpatients were also excluded.

Search strategy for identification of studies

A systematic search of the databases Cochrane Central Register of Controlled Trials (CENTRAL), Ovid MEDLINE, Ovid Embase, EBSCO CINAHL Plus and Web of Science will be conducted to identify relevant papers published from inception to October 2017. The search strategy will be based on criteria set out by the Cochrane Collaboration in a recently published systematic review and is available in the online supplementary material appendix (19). The *International Standard Randomised Controlled Trial Number (ISRCTN) Registry, a primary clinical trial registry recognised by WHO and ICMJE that accepts and records all clinical research studies in order to to improve the publicly available information about clinical studies*, will also be searched for any relevant trials. Literature search results will be exported to EndNote and then uploaded to COVIDENCE, a web-based software that facilitates collaboration among reviewers during the study selection process. Full text articles will be uploaded to COVIDENCE. Two reviewers (HH and TB) will independently assess articles to determine eligibility for inclusion and any discrepancies will be resolved by discussion or referred to a third reviewer (BS). Reference lists of included articles will be reviewed to identify other potential papers not retrieved in the initial search. Contributing authors and content experts will be asked if they have, or are aware of, any additional studies (published or unpublished).

Risk of bias assessment and certainty of evidence assessment

Two reviewers will independently assess the risk of bias of each included study. RCTs will be assessed using the Cochrane risk of bias tool that includes items on allocation bias (random sequence generation, allocation concealment, baseline imbalance), departures from intended interventions (participant and study personnel blinding, deviations from intended interventions and analysis in groups to which they were randomized), attrition bias and appropriate methods to account for missing data, detection bias (blinding of outcome assessors), and selective outcome reporting (20,21). The ROBINS-I tool will be used to assess quality of each observational study. The ROBINS-I tool contains items on bias due to confounding, selection bias, bias due to deviations from intended intervention, bias due to missing data and selective reporting (22). We will use GRADE to rate the overall certainty (quality) of evidence that includes the evaluation of risk of bias, inconsistency, indirectness, imprecision, and publication factors (23).

Data extraction and database creation

Authors of eligible trials and observational studies will be contacted via an email, or where this is not possible by a letter, outlining the study aims. They will be invited to collaborate and share their data in a format of their choice. A data sharing agreement may be provided on request. A complete database (containing data on all available measures) rather than key variables used in the publication will be requested. If resource usage and quality of life data were collected in separate forms, such information should be provided too. This will allow us to recalculate the primary outcomes where necessary to ensure a consistent approach has been used across studies with the aim of reducing the level of between-study heterogeneity. Collaborators will be able to provide the

For peer review only - http://bmjopen.bmj.com/site/about/guidelines.xhtml

data in a format of their choice. Study data will be considered unavailable in the event that none of the authors have responded to multiple contact attempts or if all contacted study authors indicate they no longer have access to the data.

Data will be checked by comparing key variables (e.g. size of sample, descriptive statistics of demographic and outcome measures) with published data. We will aim to reproduce individual study results to ensure the current analysis is consistent with what has been done in each study. Collaborators will be contacted if important discrepancies are identified and asked for clarification. The level of missing data within each included study will be assessed and discussed with study collaborators. If less than 5% of data is found to be missing on key baseline characteristics and outcome measures, the data will be analysed on a complete cases basis. However, if there is substantial missing data, the pattern and nature of the missingness will be explored and multiple imputation by chained equations (ICE) will be used if appropriate.

A copy of the data to be included in the IPD meta-analysis will be converted for analysis in STATA version 14 or above. A database will be created containing study ID, patient ID, outcomes measures, subgroups of interest, antibiotic prescribing approach and propensity scores, where necessary. Outcome measures from the different studies will be harmonised once data from all study authors have been received. In the case that coding for an outcome in a study differs greatly from other studies, it may be possible to simplify measures to allow the study to contribute data or exclude the study from certain analyses. Any difficulties with harmonisation will be discussed with the full collaborators group to decide on the most meaningful approach. Harmonised data from the different studies will be aggregated (using STATA) into a single large IPD database, with an indicator variable to identify patients from the same trial. The IPD database will be rechecked for accuracy.

The key resource information on medication, primary care consultation, hospital inpatients, hospital day cases, outpatient visits, accident and emergency (A&E) attendance from individual studies will be recorded if such information were collected and length of usage and frequency of attendance will be adjusted to the same time periods. We will use the UK national published tariff (BNF, National reference cost and PSSRU) to cost associated resource usage.

Analysis

Study and patient level characteristics will be described for all studies that contribute IPD. Characteristics of any studies that declined or were unable to provide data will be considered in order to determine the extent to which the included studies are a representative sample. A traditional meta-analysis may be performed, using data from published papers, to test for differences between studies included in the IPD meta-analysis and those that could not provide data (24).

Heterogeneity of eligible studies will be summarised using an I^2 statistic (tested by Higgins I-squared test). A substantial statistical heterogeneity will be considered if I^2 statistic is > 50% (21). Sources of heterogeneity will be explored and reported and a random effects model will be used for all analyses.

IPD meta-analysis

IPD meta-analysis will be conducted using a one-stage approach. The one-stage approach combines all the data in a single meta-analysis based on a regression model stratified by study (25). One stage analysis is often more appropriate for exploring treatment-covariate interactions as it has increased power and is less likely to suffer from aggregation bias (26). The aggregated IPD will be examined using multilevel regressions clustering on the individual study level to take into account any

For peer review only

heterogeneity between studies. Using the aggregated IPD dataset, we will calculate the key outcome measures on a consistent basis. The model selected will be one that is appropriate to the outcome measure of interest. For example, assuming the underlying assumptions of the model are met, a linear regression will be used to model the severity of symptoms, a proportional-hazards model will be used to assess the duration of illness, or a suitable count model where time to event data is not available, and logistic regression models will be used for the complications/re-consultation outcome, side effects and patient satisfaction. All models will control for baseline severity of illness and all individuals will be included on a modified intention to treat (ITT) basis (i.e. as randomised) (27).

To explore whether there are differences in treatment effects for certain sub-groups, we will consider 5 pre-specified subgroups which have been identified as potential effect modifiers by previous research: prior duration of illness (above/below median for the condition), age (under 16 years, 16-64 years, age over 65 years), fever at baseline consultation (>37.5 C), comorbid condition including lung comorbidity such as asthma or COPD, and severity of symptoms at baseline consultation. We will also explore differences according to the diagnostic group (acute sore throat, cough/chest infection, otalgia/otitis media, upper respiratory tract infection (URTI). The analyses described above will be repeated, testing for interactions between treatment allocation and sub-group characteristic.

Symptom trajectories in patients who do not take antibiotics will also be explored. For symptoms recorded in symptom diaries, we will calculate the median and inter quartile range of the reported time until all symptoms have settled completely, in those patients who did not receive an antibiotic prescription or who received a delayed prescription but did not fill it (where this data is available). As sensitivity analyses, we will also explore duration of moderately severe of symptoms. It is possible that the use of multiple outcomes (symptom severity, development of complications, patient satisfaction) to determine clinical effectiveness may result in varying conclusions. We will report the results for each outcome, though the discussion and recommendations will be based primarily on the primary outcome– symptom severity.

Work on cross design synthesis by the Program and Methodology Division of the US General Accounting Office (1992) has provided a theoretical framework for combining database data and RCT data. Drawing on this work, the current review will apply a similar approach to the synthesis of observational cohort and RCT data (28). Synthesis of observational studies will additionally use inverse probability weighting by propensity score analysis to adjust for confounding by indication on measured covariates. This serves to balance observational studies on key covariates) so that the distribution of baseline covariates is similar between treated and untreated subjects, making observational studies more like those in a RCT (29, 30). Potential covariates to be included in the propensity score include: demographic factors (e.g.: age, sex), comorbid health conditions and signs and symptoms at baseline consultation. Observational studies after this adjustment for potential confounding variables, will be included in the main analysis if appropriate. However, sensitivity analyses will explore whether the estimates from observational cohort studies differ from those found in the RCTs. If there is evidence of substantial differences, data from observational studies and RCTs will not be pooled in a single meta-analysis and reasons for these differences will be explored narratively.

Economic analysis

The economic evaluation will be taken from the NHS and PSS perspective, as most of the studies in this area have collected such data. The NHS and PSS perspective is the most commonly used approach to economic evaluations conducted in the UK. It covers medication, primary care consultation, walking in centre, NHS telephone service, outpatient attendance, accident &

For peer review only - http://bmjopen.bmj.com/site/about/guidelines.xhtml

emergency (A&E) visit and hospital admission. Outcomes will be differences in cost, costs per antibiotic prescription avoided, and costs per quality of life gained. Individual patient resource usage data such as medication use, GP consultations, outpatient visits, A&E attendance and use of secondary care service from each individual trial or observational study will be costed using UK published data sources (British National Formulary (BNF), national reference costs and Personal Social Services Research Unit (PSSRU)). Total costs for individual patients during study periods will be calculated. We will also take a societal perspective by including the societal cost of antimicrobial resistance and time of work due to illness in our modelling and sensitivity analyses.

A decision analytic model will be developed to project the potential saving (due to reduced antibiotic usage, and reduced frequency of attending primary care consultation) and cost effectiveness in terms of reduced antibiotic usage, and quality adjusted life year gained of delayed antibiotics. The model will consider re-infection and present the results at different time lengths, up to a maximum of 5 years. The model will be an individual based micro-simulation taking into account individual risk profiles with associated risk equations. The model will simulate individual treatment pathways with defined baseline characteristics and will capture the time spent at different levels of disease severity, time to recovery and recurrence rates. The probabilities of staying or transitioning to another state will be derived from the statistical analyses. The clinical analyses based on all data will provide more robust estimates to populate the input values for the data. The costs and cost effectiveness of subgroups will be explored in the decision model.

Quality of life questionnaire will be translated into utility scores based on the UK tariff appropriate to the instruments used in each study. The different instruments (such as EQ5D, SF6D or HUI) will be recorded and weighted in the aggregated analyses. Utility scores at each measurement point will be estimated and a mean utility score for symptom severity will be calculated. For studies where no quality of life data were collected, we will apply the utility scores estimated from the other studies according to symptom severity. Quality adjusted life years (QALYs) will be calculated based on an area under the curve approach. Due to the potentially skewed distribution in costs, a generalised linear mixed model will be employed to analyse costs and QALYs, controlling for baseline patient characteristics, length of follow-up and disease severity. The data will be hierarchical, with individual patients nested within studies, with study modelled as a random effect. Incremental costs and costs effectiveness and associated confidence intervals will be estimated by bootstrapping. The economic analyses will follow the same approach as the statistical analyses with calculations undertaken using a one-stage approach.

Sensitivity analyses

All analyses will be repeated using a two-step approach. The two-stage approach involves calculating the effect for each study separately and then combining the results using traditional meta- analysis techniques. Results using the one-stage and two-stage approach will be compared and any discrepancies explored. This will also allow us to include and explore the impact of the aggregate results from trials for which we are unable to obtain data, as aggregate results can only be included in a two-step approach. Aggregated data from observational studies will not be included because in the absence of full data, it will not be possible to control for confounding by indication.

Sensitivity analysis will explore whether there are differences in inferences based upon differences in the approach taken to delayed prescribing and whether there are any differences if studies at high risk of bias are excluded. We will also consider a higher cut off for presence of fever informed by the literature.

For peer review only

Given the potential long-term problem of antibiotic resistance, a sensitivity analysis will performed to incorporate the costs of antibiotic resistance in the economic analyses. However, given the difficulty previous researchers (31, 32) have had in estimating such costs we will vary the assumptions about the likely cost to see whether the inferences are modified.

Patient and Public Involvement (PPI)

The study has two PPI team members who have been actively involved in shaping the research questions and will continue to contribute to all stages of the research. Specifically, they will contribute to data harmonisation, providing advice to ensure the result will yield useful outcomes for patients and members of the public, interpretation of study findings and what they might mean for patients and the public, dissemination at relevant conferences/meetings and how best to disseminate findings to key patient and public groups. In return for their time, PPI representatives will be reimbursed in line with INVOLVE recommendations (33) and will be recognised as co-authors on research outputs.

Ethics and dissemination

Ethical approval for the study was obtained from the University of Southampton Faculty of Medicine Research Ethics Committee (Submission ID: 30068). There will be no identifiable patient data in any of the datasets and data will be stored in a password-restricted folder on the university server. The protocol of this IPD meta-analysis has been registered with the International Prospective Register of Systematic Reviews (PROSPERO) at the National Health Service Centre for Reviews and Dissemination at the University of York (Registration number: CRD42018079400). This protocol has been prepared in accordance with the PRISMA-P guidelines (34).

The results of this work will provide the best possible evidence regarding when and for whom delayed prescribing is appropriate. The results, disseminated through academic and trade journals as well as conferences, should help GPs in their decision-making about prescribing during consultations. The results will also be shared with patients and members of the public through our Patient Public Involvement collaborations. There is potential for PPI to help address the issue of patients seeking antibiotics inappropriately, which may help reduce unnecessary prescribing and in turn, antimicrobial resistance.

DISCUSSION

IPD meta-analysis is more appropriate and has higher statistical power than traditional meta-analysis to identify which key subgroups or patient characteristics may or may not benefit from delayed prescribing. Another advantage of IPD is that some of the biases in studies can be explored more fully than in aggregate data meta-analysis. For example, it is possible to check the randomization integrity and to perform a full intention to treat analysis to minimize attrition bias, even if this was not performed for the original publication. Furthermore, with the collaboration and communication required for a successful IPD, it should be possible to obtain information from the original study authors about potential sources of bias. A further advantage of IPD meta-analysis is that it allows study outcomes to be calculated in the same way and the models adjusted for similar confounders, which can reduce heterogeneity across studies.

However, there are some challenges associated with IPD meta-analysis (35). The key risk is not obtaining all the relevant data and failure to do so can result in biased results. It may be particularly difficult to obtain data for older studies, as the data may have been lost or destroyed or the lead researcher may have retired or changed field. We therefore plan to undertake a sensitivity analysis using a two-step approach which will allow us to incorporate the published estimates from any trials for which we are unable to obtain IPD into a meta-analysis. Although IPD offers the ability to

For peer review only

conduct additional and more accurate and appropriate analyses, in many cases similar results and conclusions can be drawn from IPD and standard meta-analysis (35). It is also possible that the outcome measures may not be sufficiently similar to harmonise. An initial scoping review suggest that almost all studies have collected the key outcome measures in a similar way. However, if it is deemed impossible to perform a meta-analysis with the data, a narrative review will be undertaken instead. We hope the results of the study will help GPs to communicate more effectively with patients about the normal course of illness, which patients are likely to benefit from a delayed prescribing approach, the costs of antibiotic use for RTIs, and help the general public to feel more confident about waiting to see whether their illness settles before visiting their GP.

Acknowledgements: We would like to thank the following collaborators which assisted us by allowing us to use the data from their studies: Dr Jennifer Chao, Dr David McCormick, Professor Ngaire Kerse, Ms Jacqueline Nuttall.

Author contributions: BS is the guarantor. BS conceived the original study concept and design and obtained funding from the NIHR Research for Patient Benefit (RfPB) Programme. BS and HH wrote the first draft of the manuscript. GY and SZ contributed to the section on health economics. BS, HH, TB, GY, SZ, PA, AA, BA, DB, JB, HB, MdlP, NF, DG, AH, TK, CL, GM, LM, KS, MM, and PL contributed to the concept and design of the study and manuscript editing. BS, HH, TB, GY, SZ, PA, AA, BA, DB, JB, HB, MdlP, NF, DG, AH, TK, CL, GM, LM, KS, MM, and PL read, provided feedback and approved the final manuscript.

Funding: This work was funded by the NIHR Research for Patient Benefit (RfPB) Programme, grant number: PB-PG-0416-20005. This funding will support the collation of the individual participant data, data management and analyses. The NIHR RfPB is not involved in any other aspect of the project, such as the design of the project's protocol and analysis plan, the collection and analyses. The funder will have no input on the interpretation or publication of the study results.

Data sharing statement: Search strategies can be found in the appendix.

Conflicts of interest: none declared.

For peer review only

References

1. Ridge KW, Hand K, Sharland M, Abubakar I, Livermore DM. Infections and the rise of antimicrobial resistance. London: Department of Health; 2011.
2. Public Health England. English surveillance programme for antimicrobial utilisation and resistance report. 2016.
3. National Institute for Health and Care Excellence. Prescribing of antibiotics for self-limiting respiratory tract infections in adults and children in primary care (Clinical Guideline 69); 2008.
4. Little P, Stuart B, Hobbs FD, Butler CC, Hay AD, Delaney B, et al. Antibiotic prescription strategies for acute sore throat: a prospective observational cohort study. The Lancet Infectious Diseases. 2014;14(3):213-9.
5. Spinks A, Glasziou PP, Del Mar CB. Antibiotics for sore throat. The Cochrane Database of Systematic Reviews. 2013;5(11):CD000023.
6. Kenealy T, Arroll B. Antibiotics for the common cold and acute purulent rhinitis. The Cochrane Database of Systematic Reviews. 2013; 4(6); CD000247
7. Costelloe C, Metcalfe C, Lovering A, Mant D, Hay AD. Effect of antibiotic prescribing in primary care on antimicrobial resistance in individual patients: systematic review and meta-analysis. BMJ (Clinical research ed). 2010;340:c2096.
8. Standing Medical Advisory Committee S-GoAR. The path of least resistance. 1998.
9. Cooke J, Butler C, Hopstaken R, Dryden MS, McNulty C, Hurding S, et al. Narrative review of primary care point-of-care testing (POCT) and antibacterial use in respiratory tract infection (RTI). BMJ open respiratory research. 2015;2(1):e000086.
10. Little P, Hobbs FD, Moore M, Mant D, Williamson I, McNulty C, et al. Clinical score and rapid antigen detection test to guide antibiotic use for sore throats: randomised controlled trial of PRISM (primary care streptococcal management). British Medical Journal (Clinical research ed). 2013;347:f5806.
11. Hay AD, Redmond NM, Turnbull S, Christensen H, Thornton H, Little P, et al. Development and internal validation of a clinical rule to improve antibiotic use in children presenting to primary care with acute respiratory tract infection and cough: a prognostic cohort study. The Lancet Respiratory Medicine. 2016;4(11):902-10.
12. Hay AD, Sterne JA, Hood K, Little P, Delaney B, Hollingworth W, et al. Improving the Diagnosis and Treatment of Urinary Tract Infection in Young Children in Primary Care: Results from the DUTY Prospective Diagnostic Cohort Study. Annals of Family Medicine. 2016;14(4):325-36.
13. Little P. Delayed prescribing of antibiotics for upper respiratory tract infection. British Medical Journal (Clinical research ed). 2005;331(7512):301–2.
14. Peters S, Rowbotham S, Chisholm A, Wearden A, Moschogianis S, Cordingley L, et al. Managing self-limiting respiratory tract infections: a qualitative study of the usefulness of the delayed prescribing strategy. The British Journal of General Practice : the Journal of the Royal College of General Practitioners. 2011;61(590):e579-89.
15. Ryves R, Eyles C, Moore M, McDermott L, Little P, Leydon GM. Understanding the delayed prescribing of antibiotics for respiratory tract infection in primary care: a qualitative analysis. BMJ Open. 2016;6(11):e011882.
16. Spurling GK, Del Mar CB, Dooley L, Foxlee R, Farley R. Delayed antibiotics for respiratory infections. The Cochrane Database of Systematic Reviews. 2013; 30(4):CD004417.
17. Berlin JA, Santanna J, Schmid CH, Szczech LA, Feldman HI. Individual patient- versus group-level data meta-regressions for the investigation of treatment effect modifiers: ecological bias rears its ugly head. Statistics in Medicine. 2002;21(3):371-87.
18. Lambert PC, Sutton AJ, Abrams KR, Jones DR. A comparison of summary patient-level covariates in meta-regression with individual patient data meta-analysis. Journal of Clinical Epidemiology. 2002;55(1):86-94.
19. Spurling GK, Del Mar CB, Dooley L, Foxlee R, Farley R. Delayed antibiotic prescriptions for respiratory infections. The Cochrane Database of Systematic Reviews. 2017;9:CD004417.

For peer review only

20. Savovic J, Page M, Elbers R, Hrobjartsson A, Boutron I, Reeves B, et al. A revised tool for assessing risk of bias in randomized trials (RoB 2.0). Trials Conference: 4th international clinical trials methodology conference , ICTMC and the 38th annual meeting of the society for clinical trials United kingdom. 2017;18(Supplement 1) (no pagination).
21. Higgins JPT, Green S, editors. Assessing risk of bias in included studies: The Cohrane Collaboration; 2011.
22. Sterne JA, Hernan MA, Reeves BC, Savovic J, Berkman ND, Viswanathan M, et al. ROBINS-I: a tool for assessing risk of bias in non-randomised studies of interventions. British Medical Journal (Clinical research ed). 2016;355:i4919.
23. Guyatt GH, Oxman AD, Akl EA, Vist G, Brozek J, Norris S, et al. GRADE guidelines: 1. Introduction-GRADE evidence profiles and summary of findings tables. Journal of Clinical Epidemiology. 2011;64(4): 383-94.
24. Riley RD, Simmonds MC, Look MP. Evidence synthesis combining individual patient data and aggregate data: a systematic review identified current practice and possible methods. Journal of Clinical Epidemiology. 2007;60(5):431-9.
25. Riley RD, Lambert PC, Abo-Zaid G. Meta-analysis of individual participant data: rationale, conduct, and reporting. British Medical Journal (Clinical research ed). 2010;340:c221.
26. Stewart GB, Altman DG, Askie LM, Duley L, Simmonds MC, Stewart LA. Statistical analysis of individual participant data meta-analyses: a comparison of methods and recommendations for practice. PloS One. 2012;7(10):e46042.
27. Akl EA, Kahale LA, Agoritsas T, Brignardello-Petersen R, Busse JW, Carrasco-Labra A, et al. Handling trial participants with missing outcome data when conducting a meta-analysis: a systematic survey of proposed approaches. Systematic Reviews. 2015;4:98.
28. *General Accounting Office. Cross-design synthesis: A new strategy for medical effectiveness research. March 1992. Washington DC*
29. Rosenbaum PR, Rubin DB. The central role of the propensity score in observational studies for causal effects. Biometrika. 1983;70:41-55.
30. D'Agostino RB, Jr. Propensity score methods for bias reduction in the comparison of a treatment to a non-randomized control group. Statistics in Medicine. 1998;17(19):2265-81.
31. Smith R, Coast J. The true cost of antimicrobial resistance. British Medical Journal. 2013;346.
32. Oppong R, Smith RD, Little P, Verheij T, Butler CC, Goossens H, et al. Cost effectiveness of amoxicillin for lower respiratory tract infections in primary care: an economic evaluation accounting for the cost of antimicrobial resistance. The British Journal of General Practice : the Journal of the Royal College of General Practitioners. 2016;66(650):e633-9.
33. Payment- What rates should be offered for involveent in research? National Health Research Involve website. http://www.invo.org.uk/posttypefaq/payment-what-rates-should-be-offered-for-involvement-in-research. Published 2013. Accessed September 11, 2018.
34. Shamseer L, Moher D, Clarke M, Ghersi D, Liberati A, Petticrew M, et al. Preferred reporting items for systematic review and meta-analysis protocols (PRISMA-P) 2015: elaboration and explanation. British Medical Journal (Clinical research ed). 2015;350:g7647.
35. Tudur Smith C, Marcucci M, Nolan SJ, Iorio A, Sudell M, Riley R, et al. Individual participant data meta-analyses compared with meta-analyses based on aggregate data. The Cochrane Database Systematic Reviews. 2016;9: MR000007.

For peer review only

Supplementary Online Appendix 1. CENTRAL, MEDLINE, Embase, CINAHL, and Web of Science search strategies for randomised controlled trials (November 2017)

Cochrane Central Register of Controlled Trials (CENTRAL)
#1 MeSH descriptor: [Respiratory Tract Infections] explode all trees
#2 ((upper next respiratory next tract next infection*) or URTI):ti,ab,kw
#3 MeSH descriptor: [Otitis Media] explode all trees
#4 (otitis next media):ti,ab,kw
#5 MeSH descriptor: [Pharyngitis] explode all trees
#6 pharyngitis:ti,ab,kw
#7 MeSH descriptor: [Tonsillitis] explode all trees
#8 tonsillitis:ti,ab,kw
#9 MeSH descriptor: [Common Cold] explode all trees
#10 (common next cold*):ti,ab,kw
#11 MeSH descriptor: [Bronchitis] explode all trees
#12 bronchitis:ti,ab,kw
#13 MeSH descriptor: [Sinusitis] explode all trees
#14 sinusitis:ti,ab,kw
#15 (sore next throat*):ti,ab,kw
#16 #1 or #2 or #3 or #4 or #5 or #6 or #7 or #8 or #9 or #10 or #11 or #12 or #13 or #14 or #15
#17 MeSH descriptor: [Anti-Bacterial Agents] explode all trees
#18 antibiotic*:ti,ab,kw
#19 #17 or #18
#20 (delay* near/15 prescri*):ti,ab,kw
#21 #16 and #19 and #20

Ovid MEDLINE
1 exp Respiratory Tract Infections/
2 (upper respiratory tract infection$ or urti).mp.
3 exp Otitis Media/
4 otitis media.mp.
5 exp Pharyngitis/
6 pharyngitis.mp.
7 exp Tonsillitis/
8 tonsillitis.mp.
9 exp Common Cold/
10 common cold.mp.
11 exp Bronchitis/
12 bronchitis.mp.
13 exp Sinusitis/
14 sinusitis.mp.
15 sore throat$.mp.
16 or/1-15
17 exp Anti-Bacterial Agents/
18 antibiotic$.mp.
19 or/17-18
20 (delay$ adj15 prescri$).mp.
21 16 and 19 and 20
22 (2013* or 2014* or 2015* or 2016* or 2017*).ed.
23 21 and 22

Ovid Embase
1 exp Respiratory Tract Infection/
2 exp Upper Respiratory Tract Infection/
3 (upper respiratory tract infection$ or urti).mp.
4 exp Otitis Media/
5 otitis media.mp.

For peer review only

6 exp Pharyngitis/
7 pharyngitis.mp.
8 exp Tonsillitis/
9 tonsillitis.mp.
10 exp Common Cold/
11 common cold.mp.
12 exp Bronchitis/
13 bronchitis.mp.
14 exp Sinusitis/
15 sinusitis.mp.
16 sore throat$.mp.
17 or/1-16
18 exp antibiotic agent/
19 antibiotic$.mp.
20 or/18-19
21 (delay$ adj15 prescri$).mp.
22 17 and 20 and 21
23 (2013* or 2014* or 2015* or 2016* or 2017*).em.
24 22 and 23

EBSCO CINAHL Plus
S15 S10 and S13 and S14
S14 TI delay* N15 prescri* or AB delay* N15 prescri*
S13 S11 or S12
S12 TI antibiotic* or AB antibiotic*
S11 (MH "Antibiotics+")
S10 S1 or S2 or S3 or S4 or S5 or S6 or S7 or S8 or S9
S9 TI (otitis media or pharyngitis or tonsillitis or common cold* or bronchitis or sinusitis or sore throat*) or AB (otitis media or pharyngitis or tonsillitis or common cold* or bronchitis or sinusitis or sore throat*)
S8 (MH "Sinusitis+")
S7 (MH "Bronchitis+")
S6 (MH "Common Cold")
S5 (MH "Tonsillitis+")
S4 (MH "Pharyngitis")
S3 (MH "Otitis Media+")
S2 TI (upper respiratory tract infection* or urti) or AB (upper respiratory tract infection* or urti)
S1 (MH "Respiratory Tract Infections+")

Web of Science
#15#14 AND #11 AND #10
#14#13 OR #12
#13TS=prescri*
#12TS=delay*
#11TS=antibiotic*
#10#9 OR #8 OR #7 OR #6 OR #5 OR #4 OR #3 OR #2 OR #1
#9TS="sore throat*"
#8TS=sinusitis
#7TS=bronchitis
#6TS="common cold"
#5TS=tonsillitis
#4TS=pharyngitis
#3TS="otitis media"
#2TS=urti
#1TS="Respiratory Tract Infection*"

For peer review only

Supplementary Online Appendix 2. CENTRAL, MEDLINE, Embase, CINAHL, and Web of Science search strategies for observational studies (November 2017)

Cochrane Central Register of Controlled Trials (CENTRAL)
#1 MeSH descriptor: [Respiratory Tract Infections] explode all trees
#2 ((upper next respiratory next tract next infection*) or URTI):ti,ab,kw
#3 MeSH descriptor: [Otitis Media] explode all trees
#4 (otitis next media):ti,ab,kw
#5 MeSH descriptor: [Pharyngitis] explode all trees
#6 pharyngitis:ti,ab,kw
#7 MeSH descriptor: [Tonsillitis] explode all trees
#8 tonsillitis:ti,ab,kw
#9 MeSH descriptor: [Common Cold] explode all trees
#10 (common next cold*):ti,ab,kw
#11 MeSH descriptor: [Bronchitis] explode all trees
#12 bronchitis:ti,ab,kw
#13 MeSH descriptor: [Sinusitis] explode all trees
#14 sinusitis:ti,ab,kw
#15 (sore next throat*):ti,ab,kw
#16 #1 or #2 or #3 or #4 or #5 or #6 or #7 or #8 or #9 or #10 or #11 or #12 or #13 or #14 or #15
#17 MeSH descriptor: [Anti-Bacterial Agents] explode all trees
#18 antibiotic*:ti,ab,kw
#19 #17 or #18
#20 (delay* near/15 prescri*):ti,ab,kw
#21 #16 and #19 and #20
#22 Epidemiologic Studies/
#23 exp Case-Control Studies/
#24 exp Cohort Studies/
#25 Cross-Sectional Studies/
#26 (epidemiologic adj (study or studies)).ab,ti.
#27 case control.ab,ti.
#28 (cohort adj (study or studies)).ab,ti.
#29 cross sectional.ab,ti.
#30 cohort analy$.ab,ti.
#31 (follow up adj (study or studies)).ab,ti.
#32 longitudinal.ab,ti.
#33 retrospective$.ab,ti.
#34 prospective$.ab,ti.
#35 (observ$ adj3 (study or studies)).ab,ti.
#36 adverse effect.ab,ti.
#37 #22 or #23 or #24 or #25 or #26 or #27 or #28 or #29 or #30 or #31 or #32 or #33 or #34 or #35 or #36
#38 #21 and #37

Ovid MEDLINE
1 exp Respiratory Tract Infections/
2 (upper respiratory tract infection$ or urti).mp.
3 exp Otitis Media/
4 otitis media.mp.
5 exp Pharyngitis/
6 pharyngitis.mp.
7 exp Tonsillitis/
8 tonsillitis.mp.
9 exp Common Cold/
10 common cold.mp.
11 exp Bronchitis/
12 bronchitis.mp.

For peer review only

13 exp Sinusitis/
14 sinusitis.mp.
15 sore throat$.mp.
16 or/1-15
17 exp Anti-Bacterial Agents/
18 antibiotic$.mp.
19 or/17-18
20 (delay$ adj15 prescri$).mp.
21 16 and 19 and 20
22 (2013* or 2014* or 2015* or 2016* or 2017*).ed.
23 21 and 22
24 Epidemiologic studies/
25 Exp case control studies/
26 Exp cohort studies/
27 Cross-sectional studies/
28 Case control.tw.
29 (cohort adj (study or studies or analys*)).tw.
30 Cohort analy$.tw.
31 ((Follow up or observational or uncontrolled or non randomi#ed or nonrandomi#ed or epidemiologic*) adj (study or studies)).tw
32 (longitudinal or retrospective or prospective or cross sectional) adj (study or studies or review or analys* or cohort*)).tw.
33 Longitudinal.tw.
34 Retrospective*.tw.
35 Prospective*.tw
36 Cross sectional.tw.
37 Follow-Up Studies/
38 correlational study.mp
39 Or/24-38
40 23 and 39

Ovid Embase
1 exp Respiratory Tract Infection/
2 exp Upper Respiratory Tract Infection/
3 (upper respiratory tract infection$ or urti).mp.
4 exp Otitis Media/
5 otitis media.mp.
6 exp Pharyngitis/
7 pharyngitis.mp.
8 exp Tonsillitis/
9 tonsillitis.mp.
10 exp Common Cold/
11 common cold.mp.
12 exp Bronchitis/
13 bronchitis.mp.
14 exp Sinusitis/
15 sinusitis.mp.
16 sore throat$.mp.
17 or/1-16
18 exp antibiotic agent/
19 antibiotic$.mp.
20 or/18-19
21 (delay$ adj15 prescri$).mp.
22 17 and 20 and 21
23 (2013* or 2014* or 2015* or 2016* or 2017*).em.
24 22 and 23

For peer review only

25 Clinical study/
26 exp Case control study/
27 Family study/
28 Longitudinal study/
29 Retrospective study/
30 Prospective study/
31 Randomized controlled trials/
32 30 not 31
33 Cohort analysis/
34 (Cohort adj (study or studies)).mp.
35 (Case control adj (study or studies)).tw.
36 ((Follow up or observational or uncontrolled or non randomi#ed or nonrandomi#ed or epidemiologic*) adj (study or studies)).tw
37 (longitudinal or retrospective or prospective or cross sectional) adj (study or studies or review or analys* or cohort*)).tw.
38 Follow-up/
39 38 and 33
40 correlational study.mp
41 Or/25-30,32-37,39-40
42 24 and 41

EBSCO CINAHL Plus
S24 S15 and S23
S23 or/S16-S22
S22 (observational adj (study or studies)).tw.
S21 (cohort adj (study or studies)).tw.
S20 Cross sectional studies/
S19 Nonconcurrent prospective studies/
S18 Correlational studies/
S17 Exp case control studies/
S16 Prospective studies/
S15 S10 and S13 and S14
S14 TI delay* N15 prescri* or AB delay* N15 prescri*
S13 S11 or S12
S12 TI antibiotic* or AB antibiotic*
S11 (MH “Antibiotics+”)
S10 S1 or S2 or S3 or S4 or S5 or S6 or S7 or S8 or S9
S9 TI (otitis media or pharyngitis or tonsillitis or common cold* or bronchitis or sinusitis or sore throat*) or AB (otitis media or pharyngitis or tonsillitis or common cold* or bronchitis or sinusitis or sore throat*)
S8 (MH “Sinusitis+”)
S7 (MH “Bronchitis+”)
S6 (MH “Common Cold”)
S5 (MH “Tonsillitis+”)
S4 (MH “Pharyngitis”)
S3 (MH “Otitis Media+”)
S2 TI (upper respiratory tract infection* or urti) or AB (upper respiratory tract infection* or urti)
S1 (MH “Respiratory Tract Infections+”)

Web of Science
#15#14 AND #11 AND #10
#14#13 OR #12
#13TS=prescri*
#12TS=delay*
#11TS=antibiotic*
#10#9 OR #8 OR #7 OR #6 OR #5 OR #4 OR #3 OR #2 OR #1
#9TS=“sore throat*”

For peer review only

#8TS=sinusitis
#7TS=bronchitis
#6TS="common cold"
#5TS=tonsillitis
#4TS=pharyngitis
#3TS="otitis media"
#2TS=urti
#1TS="Respiratory Tract Infection*"

For peer review only

Table 2. PRISMA-P (preferred reporting items for systematic review and meta-analysis protocols) 2015 checklist: recommended items to address in a systematic review protocol

Section and topic	*Item No*	*Checklist item*	*Page, line number where this can be found on current manuscript*
**Administrative information**			
Title:			
Identification	1a	Identify the report as a protocol of a systematic review	page 3, lines 6-7; page 5, lines 133-134
Update	1b	If the protocol is for an update of a previous systematic review, identify as such	page 6; lines 151-153
Registration	2	If registered, provide the name of the registry (such as PROSPERO) and registration number	page 10; lines 358-361
Authors:			
Contact	3a	Provide name, institutional affiliation, e-mail address of all protocol authors; provide physical mailing address of corresponding author	pages 1-2
Contributions	3b	Describe contributions of protocol authors and identify the guarantor of the review	page 11, lines 405-409
Amendments	4	If the protocol represents an amendment of a previously completed or published protocol, identify as such and list changes; otherwise, state plan for documenting important protocol amendments	N/A
Support:			
Sources	5a	Indicate sources of financial or other support for the review	page 11, lines 411-415
Sponsor	5b	Provide name for the review funder and/or sponsor	page 11, line 411
Role of sponsor or funder	5c	Describe roles of funder(s), sponsor(s), and/or institution(s), if any, in developing the protocol	page 11, lines 413-415
Introduction			
Rationale	6	Describe the rationale for the review in the context of what is already known	page 4, lines 83-95
Objectives	7	Provide an explicit statement of the question(s) the review will address with reference to participants, interventions, comparators, and outcomes (PICO)	page 5, lines 107-143
**Methods**			

For peer review only

Eligibility criteria	8	Specify the study characteristics (such as PICO, study design, setting, time frame) and report characteristics (such as years considered, language, publication status) to be used as criteria for eligibility for the review)	page 5, lines 136-149
Information sources	9	Describe all intended information sources (such as electronic databases, contact with study authors, trial registers or other grey literature sources) with planned dates of coverage	page 6, lines 148-151 & lines 153-157, 161-162
Search strategy	10	Present draft of search strategy to be used for at least one electronic database, including planned limits, such that it could be repeated	Appendix
Study records:			
Data management	11a	Describe the mechanism(s) that will be used to manage records and data throughout the review	page 6, lines 157-159
Selection process	11b	State the process that will be used for selecting studies (such as two independent reviewers) through each phase of the review (that is, screening, eligibility and inclusion in meta-analysis)	page 6, lines 159-161, 167
Data collection process	11c	Describe planned method of extracting data from reports (such as piloting forms, done independently, in duplicate), any processes for obtaining and confirming data from investigators	page 6, lines 179-195
Data items	12	List and define all variables for which data will be sought (such as PICO items, funding sources), any pre-planned data assumptions and simplifications	page 6, lines 182-185
Outcomes and prioritization	13	List and define all outcomes for which data will be sought, including prioritization of main and additional outcomes, with rationale	page 6, lines 182-185
Risk of bias in individual studies	14	Describe anticipated methods for assessing risk of bias of individual studies, including whether this will be done at the outcome or study level, or both; state how this information will be used in data synthesis	page 6, lines 167-177; page 9 lines 335-337
Data synthesis	15a	Describe criteria under which study data will be quantitatively synthesised	page 7, lines 203-205
	15b	If data are appropriate for quantitative synthesis, describe planned summary measures, methods of handling data and methods of combining data from studies, including any planned exploration of consistency (such as I2, Kendall's τ)	page 7; lines 227-230

For peer review only

	15c	Describe any proposed additional analyses (such as sensitivity or subgroup analyses, meta-regression)	page 9, lines 325-343
	15d	If quantitative synthesis is not appropriate, describe the type of summary planned	page 8, lines 280-282; page 11, lines 394-396
Meta-bias(es)	16	Specify any planned assessment of meta-bias(es) (such as publication bias across studies, selective reporting within studies)	page 7; lines 220-225
Confidence in cumulative evidence	17	Describe how the strength of the body of evidence will be assessed (such as GRADE)	page 6, lines 175-177

For peer review only

Section and topic	*Item No*	*Checklist item*	*Page, line number where this can be found on current manuscript*
**Administrative information**			
Title:			
Identification	1a	Identify the report as a protocol of a systematic review	page 3, lines 6-7; page 5, lines 133-134
Update	1b	If the protocol is for an update of a previous systematic review, identify as such	page 6; lines 151-153
Registration	2	If registered, provide the name of the registry (such as PROSPERO) and registration number	page 10; lines 358-361
Authors:			
Contact	3a	Provide name, institutional affiliation, e-mail address of all protocol authors; provide physical mailing address of corresponding author	pages 1-2
Contributions	3b	Describe contributions of protocol authors and identify the guarantor of the review	page 11, lines 405-409
Amendments	4	If the protocol represents an amendment of a previously completed or published protocol, identify as such and list changes; otherwise, state plan for documenting important protocol amendments	N/A
Support:			
Sources	5a	Indicate sources of financial or other support for the review	page 11, lines 411-415
Sponsor	5b	Provide name for the review funder and/or sponsor	page 11, line 411
Role of sponsor or funder	5c	Describe roles of funder(s), sponsor(s), and/or institution(s), if any, in developing the protocol	page 11, lines 413-415
Introduction			
Rationale	6	Describe the rationale for the review in the context of what is already known	page 4, lines 83-95
Objectives	7	Provide an explicit statement of the question(s) the review will address with reference to participants, interventions, comparators, and outcomes (PICO)	page 5, lines 107-143
**Methods**			
Eligibility criteria	8	Specify the study characteristics (such as PICO, study design, setting, time frame) and report characteristics (such as years considered, language, publication status) to be used as criteria for eligibility for the review)	page 5, lines 136-149

For peer review only

Information sources	9	Describe all intended information sources (such as electronic databases, contact with study authors, trial registers or other grey literature sources) with planned dates of coverage	page 6, lines 148-151 & lines 153-157, 161-162
Search strategy	10	Present draft of search strategy to be used for at least one electronic database, including planned limits, such that it could be repeated	Appendix
Study records:			
Data management	11a	Describe the mechanism(s) that will be used to manage records and data throughout the review	page 6, lines 157-159
Selection process	11b	State the process that will be used for selecting studies (such as two independent reviewers) through each phase of the review (that is, screening, eligibility and inclusion in meta-analysis)	page 6, lines 159-161, 167
Data collection process	11c	Describe planned method of extracting data from reports (such as piloting forms, done independently, in duplicate), any processes for obtaining and confirming data from investigators	page 6, lines 179-195
Data items	12	List and define all variables for which data will be sought (such as PICO items, funding sources), any pre-planned data assumptions and simplifications	page 6, lines 182-185
Outcomes and prioritization	13	List and define all outcomes for which data will be sought, including prioritization of main and additional outcomes, with rationale	page 6, lines 182-185
Risk of bias in individual studies	14	Describe anticipated methods for assessing risk of bias of individual studies, including whether this will be done at the outcome or study level, or both; state how this information will be used in data synthesis	page 6, lines 167-177; page 9 lines 335-337
Data synthesis	15a	Describe criteria under which study data will be quantitatively synthesised	page 7, lines 203-205
	15b	If data are appropriate for quantitative synthesis, describe planned summary measures, methods of handling data and methods of combining data from studies, including any planned exploration of consistency (such as I2, Kendall's τ)	page 7; lines 227-230
	15c	Describe any proposed additional analyses (such as sensitivity or subgroup analyses, meta-regression)	page 9, lines 325-343
	15d	If quantitative synthesis is not appropriate, describe the type of summary planned	page 8, lines 280-282; page 11, lines 394-396

For peer review only

Meta-bias(es)	16	Specify any planned assessment of meta-bias(es) (such as publication bias across studies, selective reporting within studies)	page 7; lines 220-225
Confidence in cumulative evidence	17	Describe how the strength of the body of evidence will be assessed (such as GRADE)	page 6, lines 175-177

For peer review only
